# Pyrazolyl-pyrimidones inhibit the function of human solute carrier protein SLC11A2 (hDMT1) by metal chelation†
†Electronic supplementary information (ESI) available: Methods for bioassays, details of kinetic studies, SMARTS pattern analysis, chemical synthesis procedures and compound characterization, X-ray deposition information, HPLC purity table, NMR spectra of final compounds, and SMILES list of final compounds with activities and of compounds extracted from ChEMBL. CCDC 1976759, 1976832 and 1976862 For ESI and crystallographic data in CIF or other electronic format see DOI: 10.1039/d0md00085j


**DOI:** 10.1039/d0md00085j

**Published:** 2020-06-02

**Authors:** Marion Poirier, Jonai Pujol-Giménez, Cristina Manatschal, Sven Bühlmann, Ahmed Embaby, Sacha Javor, Matthias A. Hediger, Jean-Louis Reymond

**Affiliations:** a Department of Chemistry and Biochemistry , University of Bern , Freiestrasse 3 , 3012 Bern , Switzerland . Email: jean-louis.reymond@dcb.unibe.ch; b Institute of Biochemistry and Molecular Medicine , University of Bern , Bühlstrasse 28 , 3012 Bern , Switzerland; c Membrane Transport Discovery Lab , Department of Nephrology and Hypertension , Inselspital , University of Bern Kinderklinik , Freiburgstrasse 15 , 3010 Bern , Switzerland . Email: matthias.hediger@ibmm.unibe.ch; d Department of Biomedical Research , University of Bern , Murtenstrasse 35 , 3008 Bern , Switzerland; e Department of Biochemistry , University of Zürich , Winterthurerstrasse 190 , Zürich , Switzerland

## Abstract

Solute carrier proteins (SLCs) control fluxes of ions and molecules across biological membranes and represent an emerging class of drug targets. SLC11A2 (hDMT1) mediates intestinal iron uptake and its inhibition might be used to treat iron overload diseases such as hereditary hemochromatosis. Here we report a micromolar (IC_50_ = 1.1 μM) pyrazolyl-pyrimidone inhibitor of radiolabeled iron uptake in hDMT1 overexpressing HEK293 cells acting by a non-competitive mechanism, which however does not affect the electrophysiological properties of the transporter. Isothermal titration calorimetry, competition with calcein, induced precipitation of radioactive iron and cross inhibition of the unrelated iron transporter SLC39A8 (hZIP8) indicate that inhibition is mediated by metal chelation. Mapping the chemical space of thousands of pyrazolo-pyrimidones and similar 2,2′-diazabiaryls in ChEMBL suggests that their reported activities might partly reflect metal chelation. Such metal chelating groups are not listed in pan-assay interference compounds (PAINS) but should be checked when addressing SLCs.

## Abstract 2

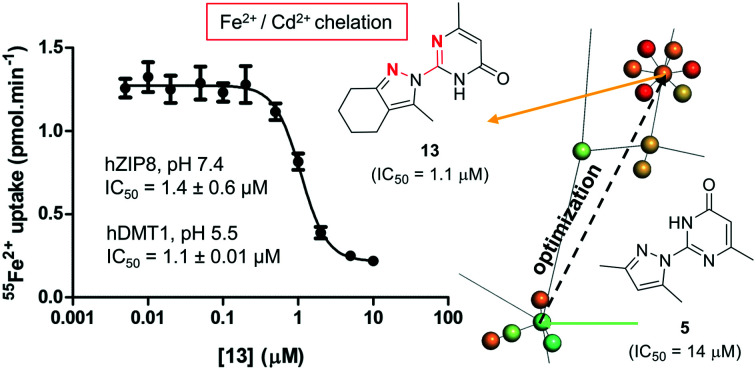
Activity optimization of a pyrazolyl-pyrimidone against the iron transporter SLC11A2 (hDMT1) and mechanistic studies revealed that this class of inhibitors act by metal chelation.

## Introduction

Solute carrier proteins (SLCs) control fluxes of ions and other molecules across biological membranes and represent an emerging class of drug targets.^1^–^3^ Here, we investigate inhibitors of the human H^+^-coupled transporter of ferrous iron (Fe^2+^), SLC11A2 (hDMT1). This transporter is expressed in the intestinal brush border membrane, where it acts as the key mediator of dietary iron uptake. hDMT1 is also linked to pathologies such as hereditary hemochromatosis, β-thalassemia, Parkinson's disease and Alzheimer's disease, highlighting that its pharmacological inhibition may be beneficial to treat human diseases.^4^–^9^ While metal chelators have been classically used to treat metal intoxication^10^ and neurodegenerative diseases,^11^,^12^ a recently reported highly potent and specific inhibitor of ferroportin (SLC40), a different iron transporter acting on the same pathway as hDMT1, has been shown to have clinical efficacy against β-thalassemia.^13^

**Fig. 1:**
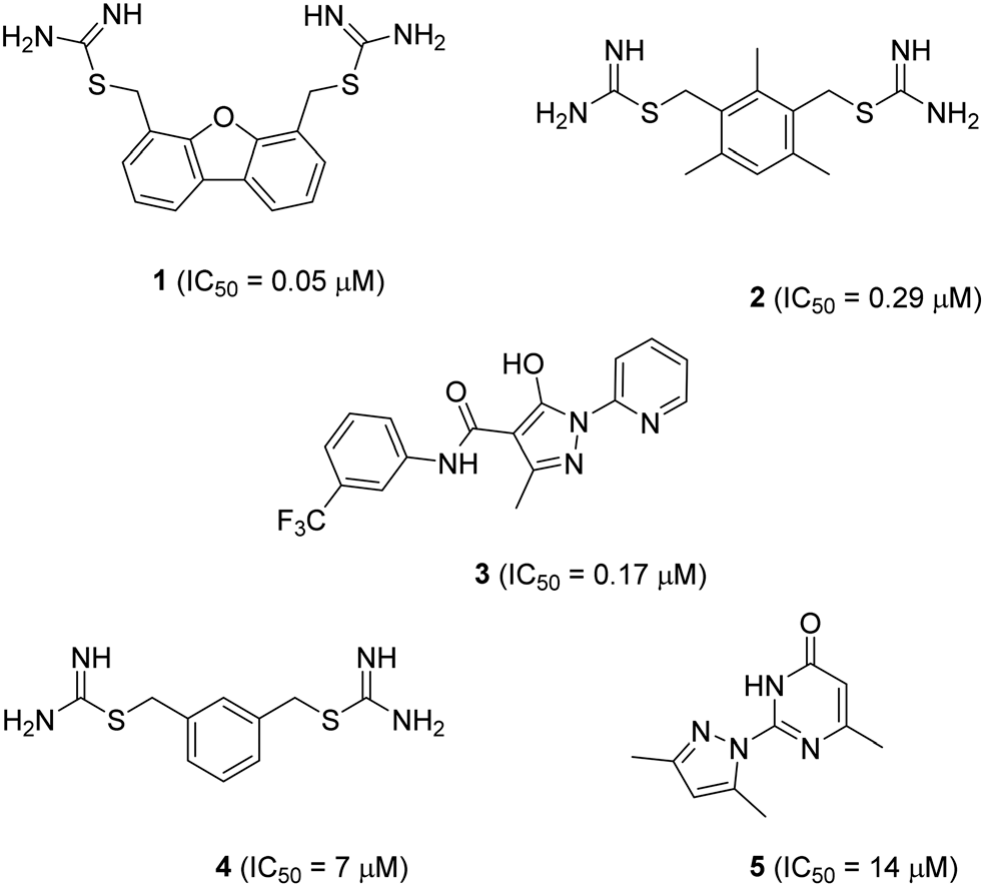
Structure and reported activity of DMT1 inhibitors. Data for inhibition of Fe^2+^ uptake into transfected HEK cells measured by calcein fluorescence assay (**1–3**) from ref. 14 and 15 or radioactive ^55^Fe^2+^ uptake assay (**4** and **5**) from ref. 17.

Two families of small molecule hDMT1 inhibitors have been reported in the literature as the results of high-throughput screening campaigns, namely bis-cationic isothioureas such as dibenzofurans **1** and mesitylene **2**,^14^ as well as pyrazolyl-pyridine **3** (Fig. 1).^15^ In our own investigations on hDMT1, we used the inhibitors mentioned above as seeds for a ligand-based virtual screening campaign guided by 3D-shape and pharmacophore similarity^16^ and discovered bis-isothiourea **4** and pyrazolyl-pyrimidone **5** as two additional hDMT1 inhibitors.^17^ Kinetic studies, an X-ray structure of a related brominated bis-isothiourea inhibitor in complex with a bacterial analog of the transporter and mutational studies recently showed that bis-isothiourea-based compounds act as competitive inhibitors of hDMT1.^18^ On the other hand, pyrazolyl-pyrimidone **5**, whose small size and better drug-like properties made it an attractive candidate compound, acted as a non-competitive inhibitor. Here we set out to investigate the pyrazolyl series (**3** and **5**) closer and understand its mechanism of action.

## Results and discussion

### 1. Optimization of pyrazolyl-pyrimidone **5**

**Scheme 1:**
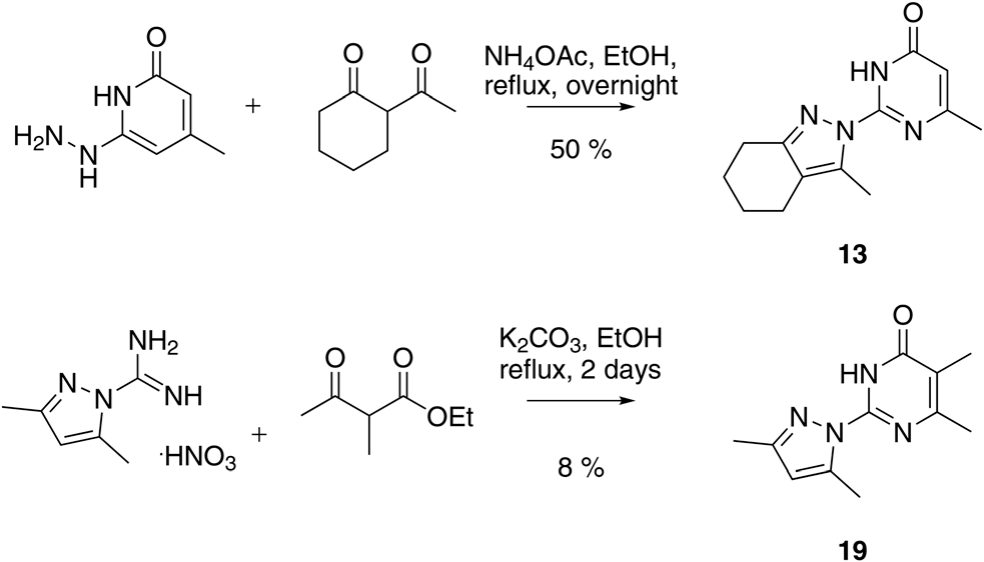
Representative synthesis of pyrazolyl-pyrimidones at the example of **13** and **19**.

**Fig. 2:**
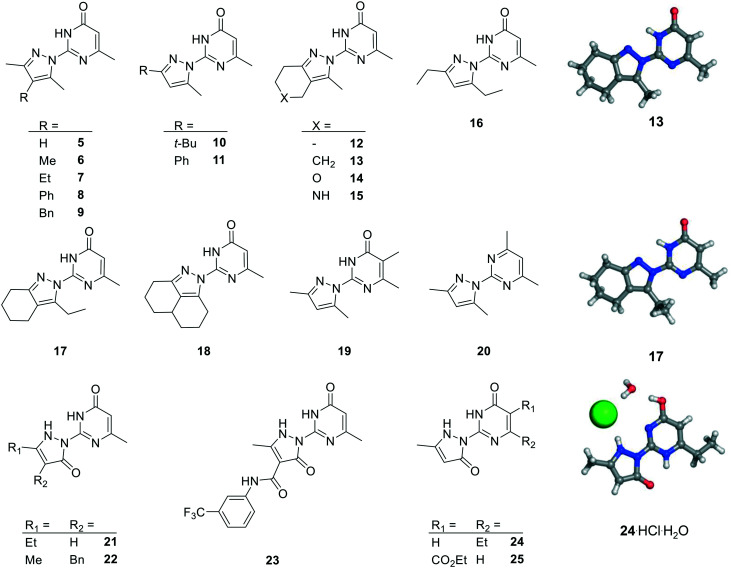
Chemical structures and X-ray crystallography of analogs of pyrazole **5**. Carbon is in gray, nitrogen in blue, oxygen in red and chlorine in green.

We prepared twenty new analogs (**6–25**) of pyrazolyl-pyrimidone **5** by condensing aminoguanidines with 1,3-diketones and keto-esters to form pyrazoles **6–20** and pyrazolones **21–25** using known chemistry (Scheme 1).^14^,^15^,^19^,^20^ For pyrazoles the synthesis gave only one product from symmetrical diketones (**5–9**, **16**, **18–20**). With non-symmetrical diketones synthesis also mostly yielded a single product. X-ray crystal structures of **13** and **17** showed that the product from non-symmetrical diketones was the isomer with the larger pyrazole substituent pointing away from the aminopyridone. By analogy, the same arrangement was assigned to the other pyrazoles. The synthesis of pyrazolones **21–25** only gave a single product whose structures were confirmed by an X-ray crystal structure of **24** as hydrochloride salt (Fig. 2).

We measured hDMT1 activity using a radioactive iron uptake assay in HEK293 cells stably overexpressing hDMT1 as described previously.^17^ Dibenzofuran inhibitor **1** showed only micromolar potency in this assay, which is 5-fold weaker than the originally reported value based on a calcein assay, while the mesitylene inhibitor **2** showed a comparable value to the originally reported inhibition (Table 1). For the present study, we selected dibenzofuran inhibitor **1** as positive control. However, we were unable to determine an IC_50_ value for the known pyrazolyl-pyridine **3** due to a lack of inhibition at high compound concentration.

**Table 1:** Activity of DMT1 inhibitors as measured by inhibition of radioactive iron uptake in HEK293 cells

Compound	IC_50_^*a*^ (μM)	Ligand efficiency
**1**	1.76 ± 0.06	0.35
**2**	0.35 ± 0.04	0.25
**3**	≫10 μM^*b*^	
**5**	13.1 ± 0.7	0.46
**6**	10.0 ± 0.4	0.44
**7**	4.2 ± 0.4	0.40
**13**	1.1 ± 0.01	0.45
**16**	3.8 ± 0.2	0.45
**17**	2.26 ± 0.3	0.42
**18**	0.94 ± 0.2	0.42
**19**	12.5 ± 0.3	0.43

^*a*^IC_50_ values were calculated using the radiolabeled iron uptake assay in HEK293 cells (source: American Type Culture Collection, catalog no. CRL-1573) stably overexpressing hDMT1. Absorbed radioactive iron (1 μM) was measured after 15 min of incubation in the presence of the indicated compound at different concentrations at extracellular pH 5.5.

^*b*^Partial inhibition was detected at 10 μM, however apparent uptake increased at higher concentrations. Compounds **8–12**, **14–15**, **20–25** showed less than 20% inhibition at 10 μM in this assay.

Activity screening of the twenty synthesized analogs of **5** for inhibition of hDMT1 revealed that seven pyrazoles (**6**, **7**, **13**, **16–19**) showed similar or improved inhibition compared to **5**. On the other hand, pyrazoles **8–12**, **14–15** and **20** as well as pyrazolones **21–25** did not show significant inhibition of iron uptake in our assay. Across all compounds tested, pyrazole **13** stood out as one of the most potent compounds with IC_50_ = 1.1 ± 0.01 μM, representing a 10-fold improvement over our initial inhibitor **5** while preserving the original ligand efficiency. Note that the bis-cyclohexane analog **18** was slightly more potent, however at the cost of higher hydrophobicity.

### 2. Characterization of pyrazolyl-pyrimidone **13**

Closer characterization of pyrazolyl-pyrimidone **13** in the radioactive ^55^Fe^2+^ uptake assay showed that this compound was a non-competitive inhibitor with similar inhibition constants *K*_i_ = 2.0 μM ([Fe^2+^] → 0) and *K*_ii_ = 1.7 μM (saturating iron), reproducing the behavior of the previously reported less potent pyrazolyl-pyrimidone **5**. By comparison, isothiourea **1** showed a competitive inhibition with inhibition constant *K*_i_ = 1.4 μM ([Fe^2+^] → 0), *K*_ii_ = 15.6 μM (saturating iron) determined by Dixon plot analysis,^21^ similarly to the previously reported less potent isothiourea **4** (Fig. S1†).

**Fig. 3:**
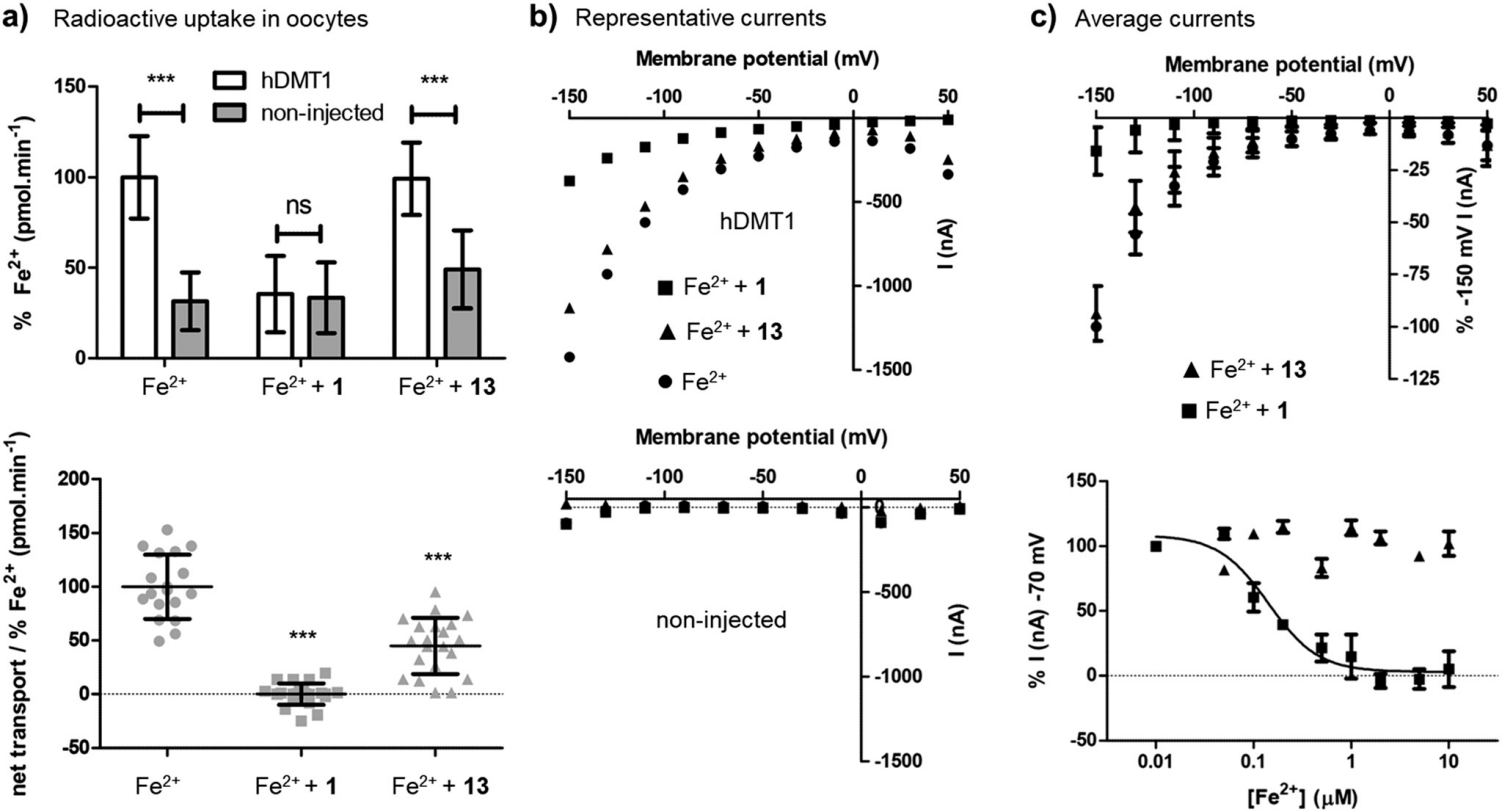
Characterization of Fe^2+^ transport inhibition by **1** and **13** in *X. laevis* oocytes (collected from frogs under license number BE60/18 conceded by the Veterinärdienst des Kantons BE, Sekretariat Tierversuche). (a) Upper panel: Average ^55^Fe^2+^-uptake (20 μM) by hDMT1-expressing and non-injected oocytes in the absence and the presence of the indicated compounds (10 μM). Data was normalized to ^55^Fe^2+^-uptake (pmol min^–1^) in the absence of compounds represented as mean ± SD (16–29 oocytes). Lower panel: Net uptake of ^55^Fe^2+^ (20 μM) by hDMT1 in the absence and the presence of the indicated compounds (10 μM). For each batch of oocytes, uptake values were corrected for unspecific iron uptake in non-injected oocytes. Data were normalized to the mean iron uptake by hDMT1 WT (pmol min^–1^) and represented as mean ± SD (14–34 oocytes). (b and c) Electrophysiology. Oocytes were held at –50 mV (*V*_h_) and a voltage-step protocol was applied (*V*_m_ = –150 mV to +50 mV) before and after the addition of Fe^2+^ (20 μM) in the absence or the presence of the indicated compounds (10 μM). For each trace, recorded currents were corrected for the background currents observed without substrate. (b) Representative traces recorded with hDMT1 injected oocytes (upper panel) and non-injected oocytes (lower panel). (c) Upper panel: Average current traces of the response of hDMT1 injected oocytes to the indicated compounds. Data was normalized to Fe^2+^ (20 μM) evoked currents at –150 mV and represented as mean ± SD (8 to 13 oocytes). Lower panel: Average dose–response (0.01–10 μM) of the inhibition of the indicated compounds over the Fe^2+^ (20 μM) evoked currents at –70 mV. Data was normalized to Fe^2+^ (20 μM) + compound (0.01 μM) evoked currents and represented as mean ± SD (4 oocytes). Kinetic parameters were obtained by fitting experimental results to a 4-parameter sigmoidal curve (black line). Compound **13** did not fit to the equation while for compound **1** the obtained IC_50_ was 0.14 μM. All the experiments were performed with oocytes from at least 3 different oocyte batches. Statistical differences were assessed using *T*-test or Mann–Whitney *U* test (Fe^2+^-uptake by hDMT1 WT *vs.* non-injected oocytes or *vs.* compound); *p* > 0.05 = ns; *p* < 0.001 = ***.

On the other hand, the uptake of radioactive iron in *Xenopus* oocytes overexpressing hDMT1 was totally inhibited by the bis-thiourea inhibitor **1**, while pyrazolyl-pyrimidone **13** only inhibited 50% of the uptake (Fig. 3a). Electrophysiological recordings in this system furthermore showed that only inhibitor **1** blocked hDMT1-induced current with a potency comparable to the iron uptake experiment (IC_50_ = 0.14 μM), but that pyrazolyl-pyrimidone **13** had no measurable effect on the recorded currents (Fig. 3b/c).

Taken together, these experiments showed that **13** was indeed a more potent version of the non-competitive inhibitor **5**. However, in contrast to the well-behaved bis-isothiourea **1**, the inhibitory effect of **13** was only detected in iron uptake experiments and was not observed in electrophysiological recordings. A similar effect had been reported for the literature inhibitor **3**.^15^

### 3. Chemical evidence for metal complex formation

At this stage we suspected that pyrazolyl-pyrimidone **13** might inhibit hDMT1 indirectly by chelating iron, although this possibility was judged unlikely for the related inhibitor **3** by lack of conclusive evidence.^15^ These inhibitors contain an N:N(:C)–C:N substructure which is potentially capable of forming a 5-membered ring chelate with metal ions *via* the two terminal sp^2^ nitrogen atoms, although this substructure is present in all the synthesized analogs including those that did not show any inhibition. The formation of such 5-membered ring chelate has been recently established by X-ray crystallography of a ruthenium complex containing both a pyrazolyl-pyridine and a pyrazolyl-pyridone ligand and investigated as C–H oxidation catalyst.^22^ Unfortunately, UV-vis titration of **13** could not be used to provide any evidence for metal complexation since the UV-vis spectrum did not significantly change upon titration with Fe^2+^ and Cd^2+^. Attempts to crystallize the iron complex of **13** as well as investigation by ^1^H-NMR and mass spectrometry similarly did not yield any indication of complex formation.

**Fig. 4:**
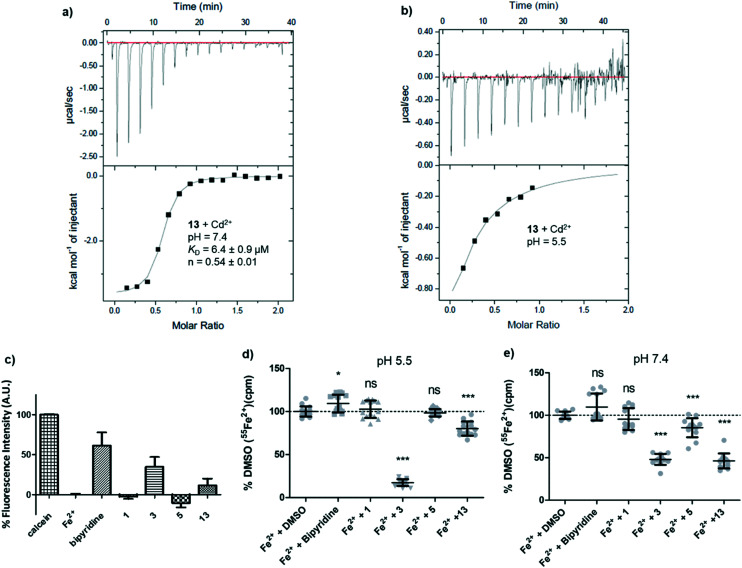
Chemical evidence of divalent metal complexation by hDMT1 inhibitors. (a and b) Isothermal titration calorimetry of pyrazole **13** (0.4 mM) with Cd^2+^ (5 mM) in aqueous buffer pH 7.4 and pH 5.5, 25 °C. (c) Calcein quenching assay in aqueous buffer pH 5.5 (2 μM Fe^2+^, 10 μM ligand, 1 μM calcein). Fe^2+^ (2 μM), ascorbic acid (200 μM) and compounds (20 μM) were incubated for 5 min before the addition of calcein (1 μM) in Krebs buffer at pH 5.5. Fluorescence was measured after 5 min incubation. (d and e) Removal of radioactive ^55^Fe^2+^ from buffers upon incubation with ligands and centrifugation. ^55^Fe^2+^ (1 μM) precipitation upon interaction with the indicated compounds (10 μM). ^55^Fe^2+^ and the indicated compounds were incubated for 15 min, then, an aliquot of the solution supernatant was taken for determination of the remaining ^55^Fe^2+^. Data was normalized to the values obtained for the Fe^2+^ + DMSO (0.1%) solution and represented as mean ± SD (*N* = 15; 2 independent experiments). Statistical differences were assessed using *T*-test or Mann–Whitney *U* test (Fe^2+^+ DMSO *vs.* each compound); *p* > 0.05 = ns; *p* < 0.05 = *; *p* < 0.001 = ***.

A first indication that pyrazolyl-pyrimidone **13** might interact with divalent metal ions was obtained by isothermal titration calorimetry (ITC) with Cd^2+^, which we used as a redox stable analog of Fe^2+^ less prone to hydroxide formation at pH = 7.4 and which is also transported by DMT1. At a pH of 7.4, the ITC data indicated an apparent dissociation constant of *K*_D_ = 6.4 μM with *n* = 0.54, consistent with the formation of a 2:1 ligand–metal complex (Fig. 4a). At pH 5.5 under which the hDMT1 transport experiment is carried out, the ITC data could not be interpreted with certainty. Upon titration of Cd^2+^ into a solution of **13**, the exothermic signal seems to saturate after the first injections, but later results in a noisy signal, indicating precipitation (Fig. 4b). The less potent pyrazolyl-pyrimidone DMT1 inhibitor **5** also interacted with Cd^2+^ at pH 7.4, although binding was much weaker (*K*_D_ ∼ 250 μM, *n* = 0.74).

A calcein competition assay to detect Fe^2+^ chelation at pH 5.5 also indicated a significant level of iron chelation by **13**, although the effect was weaker than with the positive control bipyridine or the literature inhibitor **3**, and pyrazolyl-pyrimidone **5** did not show any iron chelation in this assay (Fig. 4c). Tracking the radioactivity of ^55^Fe^2+^ in solutions after incubation with the various inhibitors indicated that pyrazolyl-pyrimidone **13** as well as the literature inhibitor **3** induced significant precipitation of Fe^2+^ at pH 7.4, while bis-isothiourea **1**, bipyridine or pyrazolyl-pyrimidone **5** had no effect. On the other hand, at pH 5.5, we only observed precipitation with the literature inhibitor **3** corresponding to the pH of the assay with hDMT1 (Fig. 4d and e). Such precipitation might indicate chelation of iron to form an insoluble complex. Taken together, these data pointed to potential iron chelating abilities of our inhibitors, however the evidence was inconclusive and did not match well with the observed inhibitory potencies.

### 4. Cross-inhibition of human SLC11A2 (hDMT1) and SLC39A8 (hZIP8)

Complex formation with Fe^2+^ should result in non-selective inhibition of different metal transporters. To test this hypothesis, we investigated whether human SLC39A8 (hZIP8), a transporter of Zn^2+^ and Fe^2+^ unrelated to SLC11 family, might also be inhibited by our hDMT1 inhibitors. Both SLCs can be tested by inhibition of radioactive iron uptake into HEK293 cells expressing the transporter, at pH 5.5 for hDMT1 and at pH 7.4 for hZIP8.

**Fig. 5:**
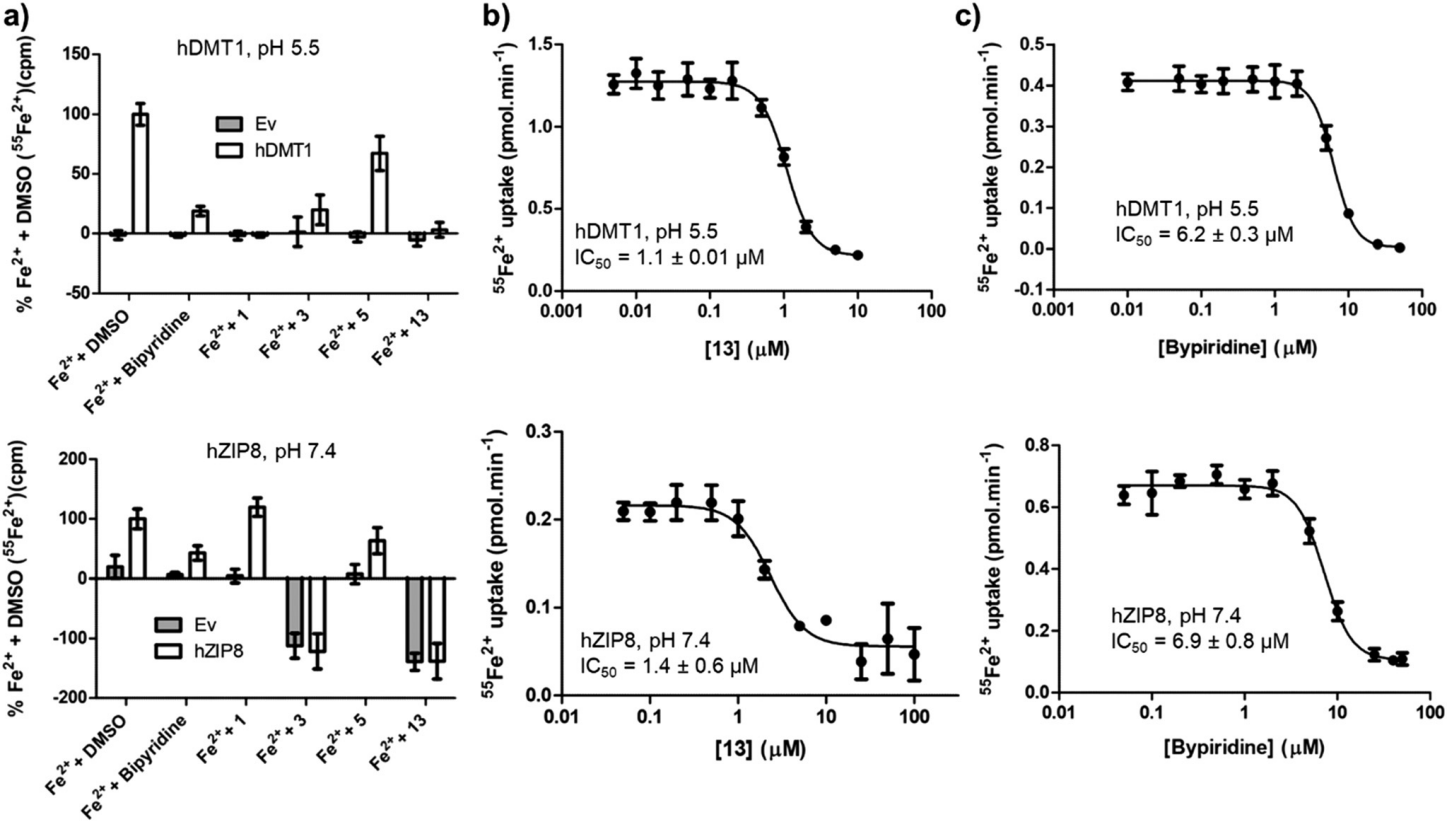
Cross reactivity of hDMT1 inhibitors over the functional activity of the divalent metal transporter hZIP8. (a) ^55^Fe^2+^ uptake (1 μM) by HEK293T cells (source: American Type Culture Collection, catalog no. CRL-3216) transiently transfected with the empty vector (Ev), hDMT1 or hZIP8. Uptake assay was performed at the optimal pH for the functional activity of each transporter (pH 5.5 and pH 7.4 respectively). The indicated compounds (10 μM) were pre-incubated for 5 min, and then, for 15 min with both compound and ^55^Fe^2+^. Measured ^55^Fe^2+^ was corrected by subtracting the background iron uptake measured in non-transfected cells. The data was normalized to the Fe^2+^ uptake measured in the presence of DMSO (0.1%) and represented as mean ± SD (*N* = 5–16; obtained from 2 independent experiments). IC_50_ determination for pyrazolyl-pyrimidone **13** (b) and bipyridine (c) in HEK293T cells transiently transfected with hDMT1 (left panels) or hZIP8 (right panels). ^55^Fe^2+^ uptake (1 μM) was measured in the presence of the indicated compound concentration ranges, and the measurements were corrected by subtracting the background iron uptake measured in non-transfected cells. IC_50_ values were calculated by fitting the data to a sigmoidal 4-parameter equation. Representative experiments are depicted, while IC_50_ values were calculated as mean ± SD of 3 different experiments.

Indeed, activity screening showed that bipyridine, a well-known iron chelator, non-specifically inhibited both transporters. Similarly, the literature pyrazolyl-pyridine **3**, our optimized pyrazolyl-pyrimidone **13**, and to a lesser extent pyrazolyl-pyrimidone **5**, all inhibited both transporters, although the inhibition by **3** was not consistently observed at higher concentrations, probably due to adsorption of the complex to the cell material and assay plate, resulting in apparent uptake. On the other hand, bis-isothiourea **1**, which inhibits hDMT1 by competing in the iron binding site, only inhibited hDMT1 and had no measurable activity on hZIP8 (Fig. 5a). A precise determination of the inhibitory effects with pyrazolyl-pyrimidone **13** and with the known iron chelator bipyridine showed that these compounds had comparable IC_50_ values on both transporters (**13**: IC_50_ ∼ 1.1 μM, bipyridine: IC_50_ ∼ 6.5 μM, Fig. 5b/c).

### 5. Mechanistic model

**Fig. 6:**
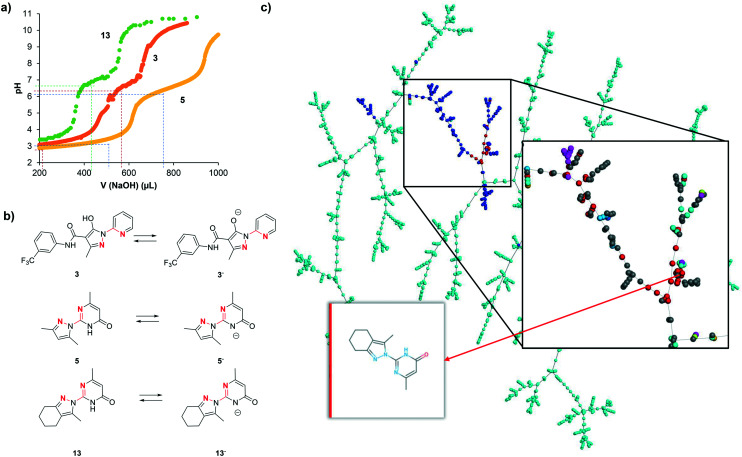
(a) Acid–base titration of **3**, **5**, **13** and neutral and basic forms of **3**, **5**, **13**. (20 μmol of compound in 10 mL (10% DMSO, ∼2 mM), **3** and **5** were acidified with HCl (50 μmol)). (b) Acid-base equilibria for inhibitors **3**, **5** and **13** highlighting potentially metal chelating nitrogen atoms in red. (c) TMAP of our inhibitors combined with 169 pyrazolyl-pyrimidones and 1717 pyrazolyl-pyridines found in ChEMBL, color coded by source: cyan = pyrazolyl-pyridines from ChEMBL, blue = pyrazolyl-pyrimidones from ChEMBL, red = this work. Insert: Close-up view around pyrazolyl-pyrimidones color-coded by target class: red = transporter, cyan = enzyme, magenta = unclassified protein, grey = unclassified target. The interactive TMAP including further color-codes (target classes, QED, *etc.*) is accessible at: ; http://tm.gdb.tools/dmt1_inhibitors/pyrimidones_pyridines/

We interpret the non-selective inhibition of bipyridine and **13** on both hDMT1 and hZIP8 as an indication that inhibition is caused by chelation of iron without specific interactions with the transporters. Metal chelation most likely involves a five-membered ring chelate with the two cyclic nitrogen atoms within the N:N(:C)–C:N substructures, similar to the well-known chelation by bipyridine forming a tight 3:1 complex with Fe^2+^. Acid–base titration of **3**, **5** and **13** shows in each case two ionizable groups (**3**: p*K*_a_1 = 3.3, p*K*_a_2 = 6.7; **5**: p*K*_a_1 = 3.6, p*K*_a_2 = 6.5; **13**: p*K*_a_1 = 3.3, p*K*_a_2 = 7.0, Fig. 6a). The transition at neutral pH (p*K*_a_2) can be attributed to the pyrazole hydroxyl group for **3** and to the pyrimidone NH group for **5** and **13**, resulting in a transition from the neutral form below pH 7 to a mono-anionic form above pH 7 (Fig. 6a). Both forms possess an N:N(:C)–C:N substructure and should have metal chelating abilities, the neutral form existing at pH 5.5 where hDMT1 transport is measured, and the anionic form existing at pH 7.4 where hZIP8 transport is measured (Fig. 6b).

However, while all pyrazolyl-pyrimidones and pyrazolyl-pyridines tested here should have comparable metal chelating abilities, only a few of them actually showed activity in the iron uptake assays with hDMT1 and hZIP8. We therefore postulate that inhibition only occurs either if metal complexation is tight, as for the reference chelator bipyridine, or if metal complexation results in the formation of an insoluble complex, as observed using radioactive ^55^Fe^2+^ with **3** and **13**. Note that, while iron uptake into hDMT1 expressing cells was partially reduced with the literature inhibitor **3**, we did not observe inhibition at higher concentrations (Table 1), probably because complexed iron was in this case absorbed on the cell material and assay plate, and resulted in apparent iron uptake. This is in line with the Fe^2+^ precipitation experiments at pH 5.5, where we only observe high precipitation of iron in the presence of compound **3**. The lack of inhibition by most of the compounds tested might therefore either reflect partial iron chelation or formation of an iron complex that partially absorbs on the cells, resulting in apparent iron uptake and no net inhibition.

### 6. Pyrazolyl-pyrimidones and pyrazolyl-pyridines in chemical space

To better understand our compound series, we surveyed the ChEMBL database, which lists 1.82 million bioactive molecules and their annotated targets,^23^ and found that 0.86% of these (15 585 molecules) contain a potentially metal chelating 2,2′-diazabiaryl group, including 1717 pyrazolyl-pyridines related to **3** and 169 pyrazolyl-pyrimidones related to **5** and **13** and annotated with bioactivities (Table 2). Among the 9.2 million screening compounds available in stock from commercial providers as listed in the ZINC database,^24^ we similarly found 0.87% (80 153 molecules) 2,2′-diazabiaryls, however with relatively fewer pyrazolyl-pyridines (4673 molecules) but more abundant pyrazolyl-pyrimidones (1960 molecules), which suggests that such 2,2′-diazabiraryl compounds are relatively easy to synthesize and drug-like. The abundance of 2,2′-diazabiaryls was significantly lower in theoretically enumerated chemical space databases such as FDB17,^25^ GDBMedChem^26^ and GDBChEMBL,^27^ reflecting the fact that these databases contain a much higher fraction of non-aromatic molecules.

**Table 2:** 2,2′-diazabiaryls in compound databases^*a*^

Database	ChEMBL24	ZINCinStock	FDB17	GDBMedChem	GDBChEMBL
Number of molecules	1 820 035	9 238 092	10 101 204	9 994 112	9 978 095
2,2′-Diazabiaryls	15 585	80 153	13 880	6241	2629
% of database	0.86%	0.87%	0.14%	0.06%	0.03%
Pyrazolyl-pyridines	1717	4673	110	72	65
% of 2,2′-diazabiaryls	11%	5.8%	0.8%	1.2%	2.5%
Pyrazolyl-pyrimidones	169	1960	0	26	0
% of 2,2′-diazabiaryls	1.1%	2.4%	0.0%	0.4%	0.0%

^*a*^Compounds were extracted using the corresponding SMARTS patterns. A TMAP of the 15 585 2,2′-diazabiaryls from ChEMBL24 is available at http://tm.gdb.tools/dmt1_inhibitors/diazabiaryls/. See also Fig. 6c–f and Table S1† for SMARTS patterns.

We recently developed interactive chemical space visualization tools that provide useful insights into polypharmacology and structure–activity relationships.^28^,^29^ To obtain a closer insight into the selected 2,2′-diazabiaryls, we created a tree-map (TMAP)^30^ representing pyrazolyl-pyridines and pyrazolyl-pyrimidones from ChEMBL (Fig. 6c). The TMAP can be interactively analyzed in-browser by loading its layout using the program Faerun.^31^ In this map each compound is represented by a point that can be color-coded according to selected properties, and the molecular structure is shown upon mouse-over by the program Smilesdrawer.^32^ The compounds are connected in a tree topology according to their structural similarities as measured by the MHFP6 molecular fingerprint.^33^ The TMAP illustrates the diversity of pyrazolyl-pyrimidones and pyrazolyl-pyridines in ChEMBL. The pyrazolyl-pyrimidones investigated here belong to the more drug-like molecules in the series, as can be appreciated by color-coding for drug-likeness using the quantitative estimate of drug-likeness (QED).^34^

The activities reported for these molecules in ChEMBL cover a diversity of target types, spanning from transporters and ion channels to enzymes and uncharacterized targets. We found 45 2,2′-diazabiaryl including 18 pyrazolyl-pyrimidones closely related to our compounds and reported with transporter activity in ChEMBL (Table S2†). The ChEMBL record indicates, among various targets, inhibition of glucose and hexose transporters identified in antiparasitic screening campaigns against *Leishmania mexicana*, tuberculosis, and *Plasmodium falciparum*.^35^ However, the screens performed were not transport assays but simple cytotoxicity tests. Considering that metal chelation is known to be a mechanism of toxicity against these parasites,^36^,^37^ one can speculate that the reported transporter inhibitory activity of these pyrazolyl-pyrimidones might in fact reflect toxicity as a result of metal chelation. Further activities reported for pyrazolyl-pyrimidones and pyrazolyl-pyridines might similarly be the consequence of metal chelation rather than the attributed activity, including a recently reported activity of **3** and **5** against cancer cells.^38^ Note that potentially metal chelating 2,2′-diazabiaryls such as pyrazolyl-pyrimidones are not listed in PAINS (pan-assay interference compounds).^39^–^41^ Indeed a systematic checking of our compounds with the PAINS filter from RDkit does not flag any molecule in the series.

## Conclusion

To date the pharmacology of divalent metal transporters is extremely limited. In view of the increasing importance of SLCs as drug targets, we investigated the inhibition of hDMT1 mediated iron transport by two previously reported inhibitors, namely pyrazolyl-pyridine **3** and pyrazolyl-pyrimidone **5**. Optimization of **5** uncovered analog **13** showing potent inhibition of hDMT1 (IC_50_ = 1.1 μM). However, this inhibitor did not modulate hDMT1-induced currents in *Xenopus* oocytes and exerted cross-inhibition of the unrelated iron transporter hZIP8.

Our combined experiments suggest that this non-selective inhibition reflects chelation of Fe^2+^ in the assay medium by these molecules to form an insoluble precipitate. Although metal chelation is often exploited as a mechanism of drug action,^10^–^12^ metal chelation is in principle an assay interference. This effect might occur across thousands of bioactive molecules reported in ChEMBL that share the same potentially chelating substructure and explain some of their reported bioactivities. Pyrazolyl-pyrimidones and related potentially metal chelating 2,2′-diazabiaryls are not considered in the list of pan-assay interference compounds (PAINS), however these should be checked whenever screening SLC targets.

## Conflicts of interest

There are no conflicts to declare.

## Supplementary Material

Supplementary information

Supplementary information

Crystal structure data
