# Supplementary material for: Pyrazolyl-pyrimidones inhibit the function of human solute carrier protein SLC11A2 (hDMT1) by metal chelation
Source: RSC Med Chem. 2020 Jun 2;11(9):1023–31. doi: 10.1039/d0md00085j (PMC7649969; doi:10.1039/d0md00085j)
Supplement: Supplementary file 1 [file MD-011-D0MD00085J-s001.pdf]

Electronic Supplementary Material (ESI) for RSC Medicinal Chemistry.
This journal is © The Royal Society of Chemistry 2020

SUPPORTING INFORMATION

Pyrazolyl-pyrimidones Inhibit the Function of Human Solute Carrier Protein SLC11A2 (hDMT1) by Metal Chelation

Marion Poirier,[a)#] Jonai Pujol-Giménez,[b), d), e)#] Cristina Manatschal,[c)] Sven Bühlmann,[a)] Ahmed Embaby,[a)] Sacha Javor,[a)] Matthias A. Hediger*[b), d), e)] and Jean-Louis Reymond*[a)]

[a)] Department of Chemistry and Biochemistry,University of Berne, Freiestrasse 3, 3012 Bern, Switzerland

[b)] Institute of Biochemistry and Molecular Medicine, University of Bern, Bühlstrasse 28, 3012 Bern, Switzerland

[c)] Department of Biochemistry, University of Zürich, Winterthurerstrasse 190, Zürich, Switzerland

[d)] Membrane Transport Discovery Lab, Department of Nephrology and Hypertension, Inselspital, University of Bern Kinderklinik, Freiburgstrasse 15, 3010 Bern, Switzerland

[e)] Department of Biomedical Research, University of Bern, Murtenstrasse 35, 3008 Bern, Switzerland

e-mail: matthias.hediger@ibmm.unibe.ch, jean-louis.reymond@dcb.unibe.ch

[#] These authors contributed equally to the work

Table of Contents:

Assays 2
Kinetic Studies 7
Cheminformatics 9
Substructure search 10
Chemistry 12
Xray deposition 29
HPLC purity 30
Reference 31
^{1}H-NMR and ^{13}C-NMR Spectra 32

Assays

Chemicals and reagents were purchased from Merck, except where indicated otherwise.

Cell Culture. Cellular experiments where conducted either with a HEK293 cell line (ATCC) stably expressing DsRED-hDMT1 plasmid or HEK293T cells (ATCC) transiently transfected with DsRED-hZIP8 or DsRED-hDMT1 plasmids. The cell line stably expressing hDMT1 was generated as described in previous works[1,2]. In both cases, cells were cultured in Dulbecco´s modified Eagle´s medium (DMEM) supplemented with 10 % foetal bovine serum (FBS), 10 mM HEPES and 1 mM Na-pyruvate under standard cell culture conditions (37 °C, 95 % humidity and air containing 5 % CO_2). When culturing the hDMT1 overexpressing cell line, to maintain stable the expression of the exogenous plasmid, culture media was also supplemented with 500 mg/mL geneticin (Life Technologies).

***Xenopus laevis* oocytes.** Human DMT1 encoding sequence containing modified Pol1 (PJMB08) vector was linearized with Nhe I (NEB). Complementary RNA (cRNA) was synthetized *in vitro* using the mMESSAGE mMACHINE T7 kit (Thermo Fisher Scientific). Defolliculated stage V-VI oocytes were obtained by laparotomy and ovariectomy as previously described.[3,4] Oocytes were microinjected with 20 ng of cRNA and maintained in modified Barth´s medium (88 mM NaCl, 1 mM KCl, 2.4 mM $NaHCO_3$, 0.82 mM $MgSO_4$, 0.66 mM $NaNO_3$, 0.75 mM $CaCl_2$, 5 mM HEPES and 5 mM MES, pH 7.5) supplemented with antibiotics. Experiments were performed 3-5 days after the injection.

Radiolabelled iron uptake assay. Cells were seeded in clear bottom, white-well, poly-D-lysine coated 96-well plates (Corning). HEK293 cells stably over-expressing hDMT1 were seeded 24 hours before the experiment at a density of 50,000 cells/well. HEK293T cells were seeded at 30,000 cells/well 48 hours before the experiment, and 24 hours after were transfected

using Lipofectamine 2000 (Life technologies) as described in the manufacturer's protocol. Before the uptake assay, culture media was aspirated and the cells were washed with the uptake solution (140 mM NaCl, 2.5 mM KCl, 1 mM $CaCl_2$, 1 mM $MgCl_2$, 1.2 mM K_2HPO_4, 10 mM glucose, 5 mM HEPES, 5 mM MES, pH 7.4). To determine the iron uptake, the cells were incubated for 15 min at room temperature (RT) with uptake solution (pH 5.5) supplemented with 100 µM Ascorbic acid, and the indicated amount of non-radioactive Ferrous iron (Fe^{2+}) and radiolabelled $^{55}Fe^{2+}$ (American Radiolabelled) (0.5 µCi/mL). To stop the uptake process, the solution containing the radiotracer was removed and cells were washed with ice-cold uptake solution (pH 7.5). To determine the iron accumulated within the cells during the incubation time, the scintillation cocktail Mycrosinth 20 (PerkinElmer) was added to each well, and the cells were incubated during 1 hour at RT under constant agitation. Radioactivity was measured using a TopCount Microplate Scintillation Counter (PerkinElmer). Counts per minute (cpm) determined by scintillation counting were transformed into influx rates using the following equation.

$$\text{Influx rate}= \frac{\text{counts/well (cpm)} \times \text{[substrate] (pM)}}{\text{total counts (cpm/L)} \times \text{uptake time (min)}}$$

To assess the inhibitory effect of the compounds under study, prior to the incubation with the iron supplemented uptake solution, cells were pre incubated for 5 minutes with the indicated compounds at the specified concentrations.

When using as expression system *Xenopus laevis* oocytes, groups of 10-15 hDMT1-expressing oocytes were used per experimental condition. First, oocytes were placed in transport medium (100 mM NaCl, 2 mM KCl, 1 mM $CaCl_2$, 1 mM MgCl, 5 mM and 5 mM MES; pH 5.5) containing 10 µM of the compounds under study, and were incubated for 5 min at RT. Then, the medium was completely replaced by transport medium containing 10 µM compound, 100 µM ascorbic Acid, 1 µM non-radioactive Fe^{2+} and 0.5 µCi/mL $^{55}Fe^{2+}$. After 10 min incubation at RT, oocytes were rinsed with ice-cold transport medium (pH 7.5) containing

250 µM non-radioactive Fe^{2+} and 100 µM Ascorbic acid. Next, oocytes were solubilized with SDS 10% and $^{55}Fe^{2+}$ content was determined by scintillation counting. To determine the total activity of the hDMT1-expressing oocytes, uptake of $^{55}Fe^{2+}$ was determined also in the absence of any compound. To stablish the background transport activity of the oocytes, all the experiments were conducted in parallel with non-injected oocytes.

Electrophysiology methods. The Two-electrode voltage-clamp (TEVC) method was used to measure the currents induced by the transport activity through the plasma membrane of hDMT1-expressing oocytes. As previously described by our group,[3–5] oocytes were placed in a chamber continuously perfused with transport solution (100 mM NaCl, 2 mM KCl, 1 mM CaCl2, 1 mM MgCl, 5 mM and 5 mM MES; pH 7.5), and were impaled with two glass microelectrodes filled with 3 mM KCl. Then, the membrane voltage was held at -50 mV (V_h), and the current (nA) required to keep V_h constant was recorded along the experiments. These currents were measured with an OC-725 amplifier (Warner Instruments), low-pass filtered at 500 Hz, digitized at 5 kHz with a Digidata 1440 data acquisition system and captured using pClamp 10 software (Axon Instruments).

Isothermal Titration Calorimetry. ITC experiments were performed with a MicroCal ITC200 system (GE Healthcare). The titrations of $CdCl_2$ to **13** were performed at 25 °C in Hepes buffer (20 mM, NaCl 150 mM) at pH 7.5 or in MES buffer (20 mM, NaCl 150 mM) at pH 5.5 with a stirring set at 750 rpm. Compounds were prepared as a stock solution in DMSO (10 mM). Final solutions at 0.4 mM were obtained by dissolving the stock solution in Hepes buffer at pH 7.5 or in MES buffer at pH 5.5. Cadmium solutions were obtained by dissolving $CdCl_2$ in Hepes buffer at pH 7.5 or in MES buffer at pH 5.5. Sample cell was filled with compound solution and cadmium solution was installed in the syringe. After 60 seconds of delay, titration with cadmium solutions started. The first injection contained 0.4 µL of cadmium solution then aliquots of 2 µL were added every 150 seconds. Control titrations using Hepes or MES buffer

containing corresponding amounts of DMSO in the cell and the same $CdCl_2$ solutions in the syringe, resulted in very small unspecific signals of less than 0.5 µcal sec^{-1} that did not saturate. Data were analyzed using the Origin ITC analysis package and errors on the reported K_D values represent fitting errors. The data were fit using a model assuming one set of binding sites.

Iron precipitation assay. Polypropylene tubes containing uptake solution (140 mM NaCl, 2.5 mM KCl, 1 mM $CaCl_2$, 1 mM $MgCl_2$, 1.2 mM K_2HPO_4, 10 mM glucose, 5 mM HEPES, 5 mM MES, pH 7.4) supplemented with 1 µM Ferrous iron (Fe^{2+}), 100 µM Ascorbic Acid, radiolabelled $^{55}Fe^{2+}$ (0.5 µCi/mL) and 10 µM of the different compounds under study were incubated for 15 minutes at RT. Then, 10 µL aliquots from each tube were transferred in quadruplicate to a clear bottom, white-well, poly-D-lysine coated 96-well plate (Corning), and $^{55}Fe^{2+}$ content was determined by scintillation counting as above described.

Calcein quenching assay. Calcein (20 mM) was dissolved in 1 M NaOH and further dilutions were carried out in uptake solution (140 mM NaCl, 2.5 mM KCl, 1 mM $CaCl_2$, 1 mM $MgCl_2$, 1.2 mM K_2HPO_4, 10 mM glucose, 5 mM HEPES, 5 mM MES, pH 7.4). Stock solutions of compounds were prepared at 10 mM in MeOH and further dilutions were done in uptake solution. Uptake solution supplemented with 4 µM Fe^{2+} and 400 µM Ascorbic acid was pre-incubated 5 min at 37 °C in the absence or presence of the indicated compounds at 40 µM before centrifugation (4000 rpm, 3 minutes). All the determinations were then performed in quadruplicates in clear bottom, black-well, 96-well plates (Corning). Next, an equal volume of uptake solution containing 2 µM Calcein was added and the plate was incubated for 5 minutes at 37°C in the dark. Fluorescence was then measured on Tecan Infinite M1000 Pro Plate Reader.

Statistics. Except when representative experiments are presented, results are shown as Mean ± Standard deviation (SD). Parametric distribution of the averaged values was assessed by

Kolmogorov-Smirnov (< 50 values) and Shapiro-Wilk tests (>50 values). When parametrically distributed, means were compared using the unpaired T-test, while for not parametric distributions, samples were compared by Mann-Whitney test. Statistic test were performed using the IBM SPSS statistics 20 software. Statistical significance was set at $p < 0.05$.

Titration with NaOH. Compounds **3** and **5** (20 µmol, 6.5-7.5 mg) were diluted in 8.5 mL Milli-Q water, 1 mL DMSO and 0.5 mL HCl 0.1 M (Final concentration of 2 mM). Compound **13** (HCl salt, 200 µL, 0.1 M in DMSO) was dissolved in 0.8 mL DMSO and 9.8 mL Milli-Q water. Then, a solution of 0.1 M NaOH was added by steps of 2 µL with a Dosimat plus (Metrohm, Zofingen, Switzerland) and pH was measured using a 692 pH/ion meter (Metrohm).

Kinetic Studies

Analysis of kinetic data – Dose-response experiments were analysed by fitting the experimental data to a 4-parameter sigmoidal equation:

$$\text{Influx rate} = \frac{\text{Bottom} + (\text{Top-Bottom})}{(1 + 10^{((\text{LogIC50-[inhibitor]})\times \text{Slp})})}$$

Where the influx rate corresponds to the measured $^{55}Fe^{2+}$ uptake (pmol·min^{-1}), [inhibitor] refers to the concentration of compound used, IC_{50} is the concentration at half-maximal effect, top and bottom are the upper and lower plateaus of the curve, and Slp is the slope factor of the curve.

hDMT1-mediated Fe^{2+} transport rate (v) data were fit to Michaelis-Menten equation.

$$v = \frac{\text{Vmax} \times [S]}{(\text{Km} + S)}$$

Were V_{max} is the limiting rate, K_m is the substrate concentration for half-saturation and [S] is substrate concentration.

To study the inhibitory mode of the compounds under study, the kinetic parameters obtained with the Michaelis-Menten equation in the presence of the compounds, were further analysed using the Lineweaver-Burk double reciprocal plot.

$$\frac{1}{v} = \frac{1}{\text{Vmax}} + \frac{\text{Km}}{\text{Vmax}} \times \frac{1}{[s]}$$

The inhibition constants K_i and K_{ii} (See kinetic scheme below) were calculated by determining the X-intercept from the equations generated by the linear regressions of the Dixon and the reciprocal limiting rate ($1/V_{max}$) vs [I] plots.

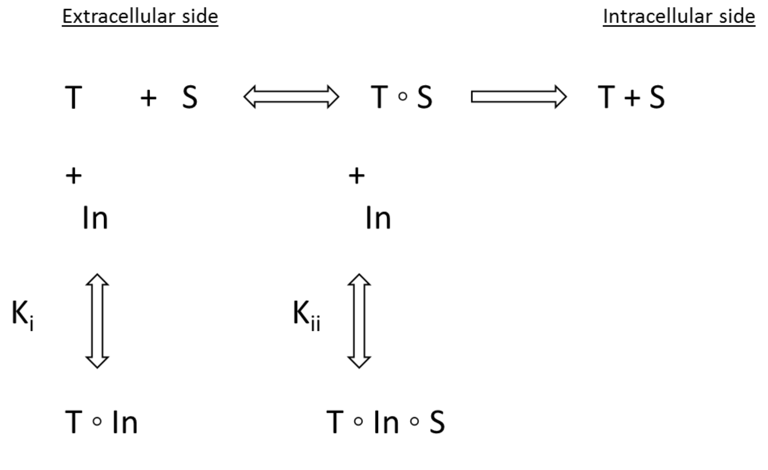

According to the proposed kinetic scheme inhibitor (In) can bind the either the empty transporter (T) or the substrate (S) / transporter complex, being the equilibrium constants for

both reactions K_i and K_{ii} respectively. All the data fitting was performed using the GraphPad Prism 5 software.

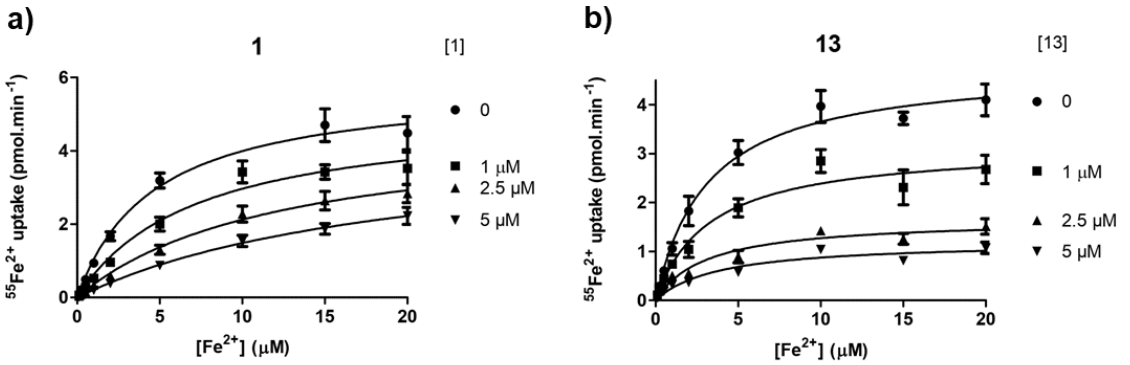

[1] (μM)	K_m (μM)	V_{max}
0	4.4 ± 1.2	6.1 ± 0.5
1	7.1 ± 1.7	5 ± 0.5
2.5	12.1 ± 1.7	4.6 ± 0.3
5	19.6 ± 1.6	4.4 ± 0.2

[13] (μM)	K_m (μM)	V_{max}
0	3.1 ± 0.4	4.7 ± 0.18
1	3.2 ± 0.8	3.1 ± 0.26
2.5	3.0 ± 0.8	1.6 ± 0.14
5	3.8 ± 1.3	1.2 ± 0.13

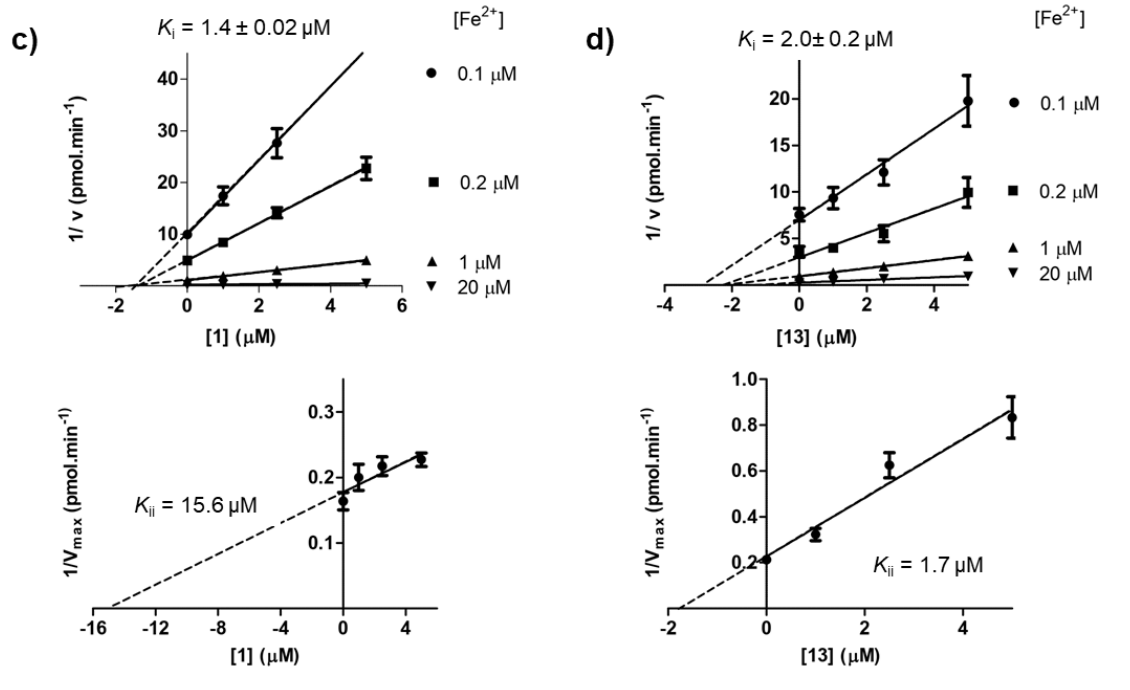

Figure S1. Inhibition of iron uptake by isothiourea **1** and pyrazole **13** in HEK293 cells stably overexpressing hDMT1. (a) Iron transport kinetics inhibition by **1 (a)** and **13 (b)**. Effect of the indicated concentrations of **1** and **13** on hDMT1-mediated Fe^{2+} uptake kinetics (upper panels). Summary of the effect of the inhibitors over the kinetic properties of the hDMT1- mediated Fe^{2+} uptake calculated with the Hill equation (lower panels). hDMT1 transport activity inhibition constants for **1** (**c**) and **13** (**d**). Dixon plot (upper panels), reciprocal velocity (1/v) plotted against the inhibitory concentration at the indicated Fe^{2+} concentrations. Linear dependence of the reciprocal limiting rate ($1/V_{max}$) on the indicated inhibitor concentrations (lower panels).

Cheminformatics

ChEMBL compound selection. ChEMBL24 was filtered for compounds containing substructures that match the SMARTS patterns listed in Table S1. The work was done using rdkit (2019.03.4).

TMAP generation. To create graphical representations of the discussed chemical space, we used TMAP as available at http://tmap.gdb.tools. All structures were encoded as 512 dimensional MHFP6 arrays. This was accomplished by first using rdkit (2019.03.4) to extract circular substructures up to radius 3 followed by employment of the MinHash functionality of the TMAP python module. Generation of the MHFP based LSH forest, as well as the resulting kNN based minimum spanning tree were also accomplished by using the TMAP module (parameters: $k = 50$, $k_c = 50$). The final TMAP was rendered using Faerun.

Substructure search

Databases were searched for 2,2'-diazabiaryls by using a series of SMARTS as follows:

Table S1: SMARTS searches in databases.

	**ChEMBL 24**	**ZINC_inStock**	**FDB17**	**GDBMedChem**	**GDBChEMBL**
**Total Cpds**	1,820,035	9,238,092	10,101,204	9,994,112	9,978,095
[cr5,cr6]:[cr5,cr6](:[nr5])!@[cr5](:[nr5]):[cr5,cr6]	366	2,913	6,005	1,750	133
[cr5,cr6]:[cr5,cr6](:[nr6])!@[cr5](:[nr5]):[cr5,cr6]	3,125	8,749	2,423	1,707	326
[cr5,cr6]:[cr5,cr6](:[nr6])!@[cr5](:[nr5]):[nr5,nr6]	2,591	11,735	257	256	337
[cr5,cr6]:[cr5,cr6](:[nr6])!@[cr6](:[nr6]):[cr5,cr6]	1,332	3,074	140	300	119
[nr5,nr6]:[cr5,cr6](:[nr6])!@[cr6](:[nr6]):[cr5,cr6]	498	3,596	9	97	59
[nr5,nr6]:[cr5,cr6](:[nr6])!@[cr6](:[nr6]):[nr5,nr6]	71	39	0	6	12
[nr5,nr6]:[cr5,cr6](:[nr6])!@[cr5](:[nr5]):[nr5,nr6]	44	578	7	31	92
[cr5,cr6]:[nr5,nr6](:[nr5])!@[cr5](:[nr5]):[cr5,cr6]	0	0	0	0	0
[nr5,nr6]:[cr5,cr6](:[nr5])!@[cr5](:[nr5]):[nr5,nr6]	54	832	104	35	69
[cr5,cr6]:[nr5,nr6](:[nr6])!@[cr5](:[nr5]):[cr5,cr6]	0	2	68	35	0
[cr5,cr6]:[nr5,nr6](:[nr6])!@[cr6](:[nr6]):[cr5,cr6]	0	0	0	0	0
[cr5,cr6]:[cr5,cr6](:[nr5])!@[nr5](:[nr5]):[cr5,cr6]	0	82	3	0	0
[cr5,cr6]:[cr5,cr6](:[nr6])!@[nr5](:[nr5]):[cr5,cr6]	2,559	15,701	224	237	350
[cr5,cr6]:[cr5,cr6](:[nr6])!@[nr6](:[nr6]):[cr5,cr6]	0	0	0	0	0
[cr5,cr6]:[nr5,nr6](:[nr5])!@[cr6](:[nr6]):[nr5,nr6]	967	7,600	13	21	95
[cr5,cr6]:[cr5,cr6](:[nr5])!@[cr6](:[nr6]):[nr5,nr6]	106	285	178	247	109
[cr5,cr6]:[nr5,nr6](:[nr5])!@[cr5](:[nr5]):[nr5,nr6]	135	1,591	237	41	11
[cr5,cr6]:[cr5,cr6](:[nr6])!@[nr5](:[nr5]):[nr5,nr6]	50	130	12	43	110
[cr5,cr6]:[nr5,nr6](:[nr5])!@[cr5](:[nr5]):[sr5]	413	4,108	113	10	19
[cr5,cr6]:[cr5,cr6](:[nr5])!@[cr5](:[nr5]):[sr5]	337	2,545	661	180	99
[cr5,cr6]:[nr5,nr6](:[nr6])!@[cr5](:[nr5]):[sr5]	8	32	4	2	3
[cr5,cr6]:[cr5,cr6](:[nr6])!@[cr5](:[nr5]):[sr5]	456	2,962	201	138	108
[nr5,nr6]:[cr5,cr6](:[nr6])!@[cr5](:[nr5]):[sr5]	33	755	13	19	45
[nr5,nr6]:[cr5,cr6](:[nr5])!@[cr5](:[nr5]):[sr5]	221	176	77	35	103
[sr5]:[cr5](:[nr5])!@[cr5](:[nr5]):[sr5]	80	140	0	14	17
[nr5,nr6]:[cr5,cr6](:[nr5])!@[cr5](:[nr5]):[or5]	35	98	72	46	50
[cr5,cr6]:[cr5,cr6](:[nr5])!@[cr5](:[nr5]):[or5]	723	7,156	556	245	73
[cr5,cr6]:[nr5,nr6](:[nr5])!@[cr5](:[nr5]):[or5]	3	139	87	21	0
[cr5,cr6]:[cr5,cr6](:[nr6])!@[cr5](:[nr5]):[or5]	325	2,946	177	151	78
[or5]:[cr5](:[nr5])!@[cr5](:[nr5]):[or5]	4	128	0	21	3
[nr5,nr6]:[cr5,cr6](:[nr5])!@[nr5](:[nr5]):[nr5,nr6]	25	7	10	5	14
[cr5,cr6]:[cr5,cr6](:[nr5])!@[nr5](:[nr5]):[nr5,nr6]	4	21	220	66	64
Multiple SMARTS patterns	1,020	2,033	2,009	482	131
**Total 2,2'-diazabiaryls**	15,585	80,153	13,880	6,241	2,629
**Pyrazolyl-pyridines:**					
c1:c:c:n:n:1!@c2:n:c:c:c:c:2	1717	4673	110	72	65
**Pyrazolyl-pyrimidones:**					
c1:c:c:n:n:1!@c2:n:c(=O):c:c:n:2	169	1960	0	26	0

Table S2: 2,2'-diazabiaryls from ChEMBL reported with transporter inhibition activity.

SMILES	CHEMBL_ID
Cc1cc(C)n(n1)c2nnc(N\N=C/c3cccnc3)nn2	CHEMBL390525
Cc1ccc(Nc2cc(C)nn2c3nc(C)cc(O)n3)cc1	CHEMBL586135
CCC(C(=O)Nc1cc(C)nn1C2=NC(=C(CC)C(=O)N2)C)c3ccccc3	CHEMBL588727
CC1=CC(=O)NC(=N1)n2nc(C)cc2NC(=O)C3(CCCC3)c4ccc(Cl)cc4	CHEMBL587830
Cc1cc(C)n(n1)c2nc(Nc3cccc(c3)C(F)(F)F)nc(n2)n4nc(C)cc4C	CHEMBL529042
CCC1=C(C)N=C(NC1=O)n2nc(C)cc2NC(=O)CC(c3ccccc3)c4ccccc4	CHEMBL586274
CC1=CC(=O)NC(=N1)n2nc(C)cc2NC(=O)C(c3ccccc3)c4ccccc4	CHEMBL529312
CCC1=C(C)N=C(NC1=O)n2nc(C)cc2NC(=O)c3ccc(OC)cc3OC	CHEMBL586186
CCCC1=CC(=O)NC(=N1)n2nc(C)cc2NC(=O)C(CC)c3ccccc3	CHEMBL524606
Cc1cc(cc(n1)n2ncc(c2N)c3ccc(cc3)C(=O)NCCNCc4ccccc4)C(F)(F)F.OC(=O)C(F)(F)F	CHEMBL530774
CCOc1cc(C=NNc2nc(C)cc(n2)n3nc(C)cc3C)ccc1O	CHEMBL530893
CCC1=CC(=O)NC(=N1)n2nc(C)cc2NC(=O)CC(c3ccccc3)c4ccccc4	CHEMBL532439
CCOc1ccc2ccccc2c1C(=O)Nc3cc(C)nn3C4=NC(=CC(=O)N4)C	CHEMBL534210
CN(C1CCCCC1)C(=O)c2nn(c3ccccn3)c4nccc(c5cccs5)c24	CHEMBL532232
CCOC(=O)c1nc([nH]c1n2nc(C)cc2C)c3ccccc3	CHEMBL533806
CCC1=C(C)N=C(NC1=O)n2nc(C)cc2NC(=O)c3cccc4ccccc34	CHEMBL588248
CC1=CC(=O)NC(=N1)n2nc(C)cc2NC(=O)CC(c3ccccc3)c4ccccc4	CHEMBL546912
Nc1nc(nc(n1)n2nc(cc2N)c3cccc(c3)C(F)(F)F)c4ccccn4.OC(=O)C(F)(F)F	CHEMBL547445
CCC1=C(C)N=C(NC1=O)n2nc(C)cc2NC(=O)C3c4ccccc4Oc5ccccc35	CHEMBL582197
CCC1=C(C)N=C(NC1=O)n2nc(C)cc2NC(=O)CCc3ccccc3	CHEMBL580305
Cc1cc(cc(n1)n2ncc(c2N)c3ccc(cc3)C(=O)NCc4ccccc4N)C(F)(F)F.OC(=O)C(F)(F)F	CHEMBL580809
CCCC1=CC(=O)NC(=N1)n2nc(C)cc2NC(=O)CC(c3ccccc3)c4ccccc4	CHEMBL549250
CCCC1=CC(=O)NC(=N1)n2nc(C)cc2NC(=O)c3oc4ccccc4c3	CHEMBL537715
CC1=C(C)C(=O)NC(=N1)n2nc(C)cc2NC(=O)c3cc(Br)ccc3Cl	CHEMBL534821
CN(C)CCC(c1ccc(Cl)c(Cl)c1)n2nnc(n2)n3nccn3	CHEMBL1223579
CNCCC(c1ccc(Cl)c(Cl)c1)n2nnc(n2)n3nccn3	CHEMBL1223580
CNCC[C@H](c1ccc(Cl)c(Cl)c1)n2nnc(n2)n3nccn3	CHEMBL1223581
CNCC[C@@H](c1ccc(Cl)c(Cl)c1)n2nnc(n2)n3nccn3	CHEMBL1224383
Cc1cc(NC(=O)c2cccc(c2)C(F)(F)F)n(n1)c3nc(cs3)c4ccc5OCOc5c4	CHEMBL2028054
Cc1cccc(c1)n2ncc3c(O)nc(nc23)n4nc(C)cc4NC(=O)c5oc6ccccc6c5	CHEMBL2028064
CC1=C(Cc2ccc(cc2)C(C)(C)C)C(=O)N(N1)c3nc(C)cc(C)n3	CHEMBL3144824
COc1ccc(cc1)C2=C(C)NN(C2=O)c3ccccn3	CHEMBL3145263
CCN(CC)c1ccc(cc1)C2=C(C)NN(C2=O)c3ccccn3	CHEMBL3145264
CC1=C(C(=O)N(N1)c2ccccn2)c3ccc(cc3)C#N	CHEMBL3145265
CC1=C(C(=O)N(N1)c2ccccn2)c3ccc(cc3)[N+](=O)[O-]	CHEMBL3145266
CC1=C(C(=O)N(N1)c2ccccn2)c3ccc(cc3)C(F)(F)F	CHEMBL3145267
CC1=C(C(=O)N(N1)c2ccccn2)c3cccc(c3)C(F)(F)F	CHEMBL3145268
CCOC(=O)c1ccc(cc1)C2=C(C)NN(C2=O)c3ccccn3	CHEMBL3145269
CC1=C(C(=O)N(N1)c2ccccn2)c3ccccc3	CHEMBL3145270
CC1=C(C(=O)N(N1)c2ccccn2)c3ccc(N)cc3	CHEMBL3145272
CC1=C(C(=O)N(N1)c2ccccn2)c3ccc(F)cc3	CHEMBL3145273
CC1=C(C(=O)Nc2cccc(c2)C(F)(F)F)C(=O)N(N1)c3ccccn3	CHEMBL3145276
COc1ccccc1N2CCN(CC2)c3ncnc4c3cnn4c5ccccn5	CHEMBL3780349
COc1ccccc1N2CCN(CC2)c3ncnc4c3cnn4c5ncccc5F	CHEMBL3781535
Oc1cc(nn1c2ccccn2)c3ccccc3	CHEMBL3828444

Chemistry

General remarks. All reagents were purchased from commercial sources and were used without further purification. Flash chromatography purifications were performed with silica Gel 60 (Sigma, 0.040–0.063 nm, 230–400 mesh ASTM). Low resolution mass spectra were obtained by electron spray ionization (ESI-MS) in the positive mode on a Thermo Scientific LCQ Fleet. High resolution mass spectra were obtained by electron spray ionization (HR-ESI-MS) in the positive mode recorded on a Thermo Scientific LTQ Orbitrap XL. ^{1}H and ^{13}C-NMR spectra were measured on a Bruker Avance 300 spectrometer (at 300 MHz and 75 MHz, respectively) or on a Bruker Avance II 400 spectrometer (at 400 MHz and 100 MHz, respectively). ^{1}H and ^{13}C chemical shifts are given in ppm (δ) relative to the solvent proton impurity signals, and resonance multiplicities are reported as s (singlet), d (doublet), t (triplet), q (quartet), p (pentet), and m (multiplet); br = broad peak. Compound purities were assessed by analytical reversed phase HPLC (RP-HPLC) at a detection wavelength of 214 nm. Analytical RP-HPLC was performed on a Dionex Ultimate 3000 RSLC System (DAD-3000 RS Photodiode Array Detector) using a Dionex Acclaim RSLC 120 column (C18, 3.0 x 50 mm, particle size 2.2 μm, 120 Å pore size) and a flow rate of 1.2 mL min^{-1}. Data were recorded and processed with Dionex Chromeleon Management System (version 6.8) and Xcalibur (version 2.2, Thermo Scientific). Eluents for analytical RP-HPLC were as follows: (A) milliQ-deionized water containing 0.05 % TFA, (D) HPLC-grade acetonitrile/milliQ-deionized water (9:1) containing 0.05 % TFA. Conditions for analytical RP-HPLC were as follows: in 2.2 min from 100 % A to 10 0% D, then staying on 100 % D (method A) in 7.5 min from 10 0% A to 100 % D, then staying on 100 % D (method B). GC analyses were carried out on a *Macherey-Nagel* Optima delta-3-0.25 μm capillary column (20 m, 0.25 mm); carrier gas: He 1.4 mL/min; injector: 220 °C split mode; detector: FID 280 °C, H2 35 mL/min, air 350 mL/min. Preparative RP-HPLC were performed with a Waters Prep LC System Controller with a Dr. Maisch GmbH

Reprospher column (C18-DE, 100×30 mm, particle size 5 µm, pore size 100 Å, flow rate 40 mL/min). Compounds were detected by UV absorption at 214 nm using Waters 2489 UV/Vis detector. The elution solutions were: (A) MilliQ deionized water containing 0.1% TFA; (D) MilliQ deionized water / acetonitrile (10 / 90) containing 0.1 % TFA.

Procedure A:[6,7]

1,3–diketone or 1,3 keto-ester (1.1 eq) was added in a suspension of 2-hydrazin-6-methyl-4(3*H*)-pyrimidinone (1.0 eq) in ethanol (5.0 mL). The mixture was stirred at 80 °C for the time indicated for each compound (2.5 h – overnight). The solution was cooled down to room temperature and the solvent was evaporated under reduced pressure. The procedure was used for pyrazoles **5-20** and pyrazolones **21-25**.

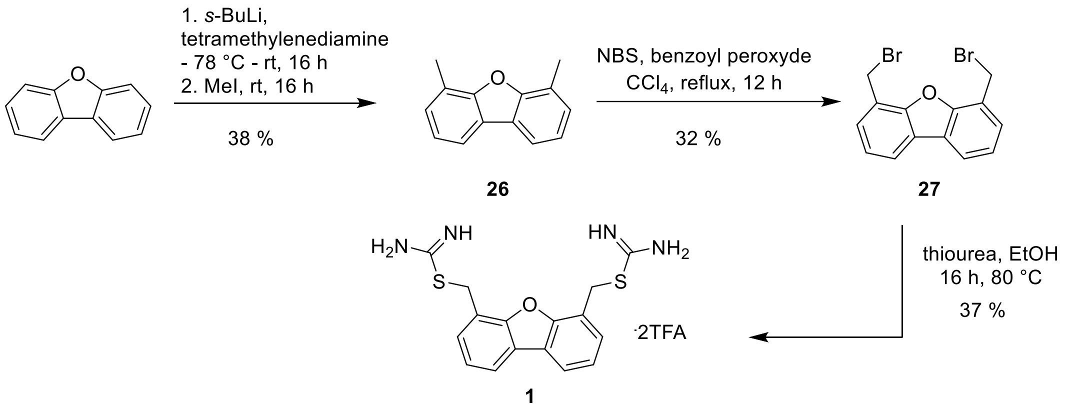

Scheme S1: Synthesis of compound **1**.

Dibenzo[b,d]furan-4,6-diylbis(methylene) dicarbamimidothioate, TFA salt (1):[8] Compound **27** (43 mg, 0.12 mmol, 1.0 eq) and thiourea (20 mg, 0.26 mmol, 2.1 eq) were suspended in ethanol (2 mL) and stirred at 80 °C for 18 h. Then, the solution was cooled down to room temperature. Ethanol was evaporated under reduced pressure until around 1 mL, which was kept, and hexane was added. The precipitate was filtered, washed with diethyl ether / ethyl acetate (50 / 50) and dried *in vacuo*. The residue was purified by preparative HPLC (gradient

from 95/5 A/D to 60/40 A/D in 30 min, t_R = 25 % D) to afford **1** (26 mg, 0.045 mmol, 37 %) as a white powder. RP-UPLC: t_R = 2.79 min (method B). ^{1}H NMR (400 MHz, DMSO): δ 9.33 (s, 6H), 8.23 – 8.16 (3H, m), 7.81 – 7.63 (3H, m), 4.93 (4H, s). ^{13}C NMR (100 MHz, DMSO): δ 168.6, 154.2, 128.6 (CH), 123.3, 123.2, 123.1, 119.0, 30.0 (CH_2). HR-MS calcd for $C_{16}H_{17}N_4OS_2$: *m/z* 345.0838, *m/z* found 345.0824.

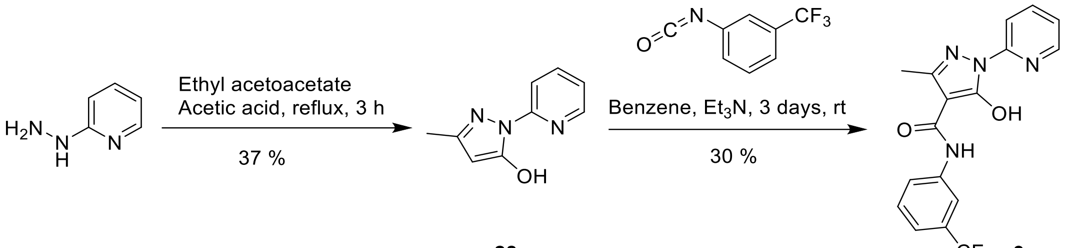

Scheme S2: Synthesis of compound **3**.

5-Methyl-3-oxo-2-(pyridin-2-yl)-*N*-(3-(trifluoromethyl)phenyl)-2,3-dihydro-1*H*-pyrazole-4-carboxamide (3):[9] Compound **28** (232 mg, 1.32 mmol, 1.0 eq), 1-isocyanato-3-(trifluoromethyl)benzene (0.19 ml, 1.3 mmol, 1.0 eq) and triethylamine (0.18 ml, 1.3 mmol, 1.0 eq) were dissolved in benzene (10 mL). The mixture was stirred at room temperature for 3 days. The solvent was evaporated under reduced pressure. The residue was dissolved in A and D. The addition of water gave a yellow precipitate which was filtered and purified by flash column chromatography on silica gel (gradient DCM / MeOH) to afford **3** (157 mg, 1.3 mmol, 32 %) as a yellow solid. ^{1}H NMR (400 MHz, DMSO): δ 10.73 (s, 1H), 8.50 – 8.49 (1H, m), 8.42 – 8.40 (1H, d, J = 8.4 Hz), 8.27 (1H, s), 8.07 – 8.03 (1H, td, J = 7.6, 2 Hz), 7.69 – 7.67 (1H, d, J = 8.0 Hz), 7.57 – 7.53 (1H, t, J = 8.0 Hz), 7.39 – 7.34 (2H, m), 2.56 (3H, s). ^{13}C NMR (100 MHz, DMSO): δ 161.7, 161.2, 151.2, 147.1, 146.8 (CH_{ar}), 140.0, 139.7 (CH_{ar}), 130.1 (CH_{ar}), 130.0 (q, J_{CF} = 24 Hz), 124.0 (q, J_{CF} = 273 Hz, CF_3), 122.4 (CH_{ar}), 121.0 (CH_{ar}), 119.1 (q, J_{CF} = 4 Hz, CH_{ar}), 115.0 (q, J_{CF} = 4 Hz, CH_{ar}), 112.0 (CH_{ar}), 96.6, 12.5 (CH_3). HR-MS calcd for $C_{17}H_{13}F_3N_4NaO_2$: *m/z* 385.0888, *m/z* found 385.0883.

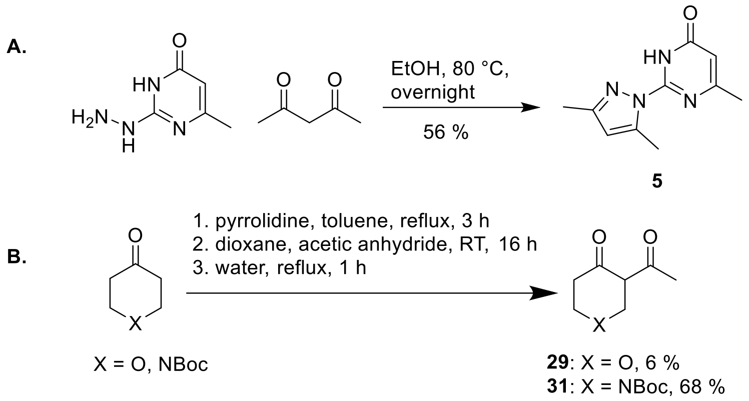

Scheme S3: A. Synthesis of compound **5** and analogues **6 - 18. B.** Synthesis of intermediate 1,3-diketones **29** and **31**.

2-(3,5-Dimethyl-1*H*-pyrazol-1-yl)-6-methylpyrimidin-4(3*H*)-one, TFA salt (5): Following general procedure A, using acetylacetone (40 µL, 0.39 mmol, 1.1 eq), after overnight stirring, pyrazole **5** (63 mg, 0.20 mmol, 56 %) was isolated as a white powder after purification by preparative HPLC (gradient from 80/20 A/D to 60/40 A/D in 18 min, t_R = 57 % D). RP-UPLC: t_R = 1.71 min (method A). ^{1}H NMR (300 MHz, MeOD): δ 6.13 (1H, s), 6.11 (1H, s), 2.63 (3H, s), 2.31 (3H, s), 2.25 (3H, s). ^{13}C NMR (75 MHz, MeOD): δ 167.8, 165.3, 153.6, 150.3, 145.3, 112.4 (CH), 108.3 (CH), 22.8 (CH_3), 15.1 (CH_3), 13.5 (CH_3). HR-MS calcd for $C_{10}H_{13}ON_4$: *m/z* 205.1084, *m/z* found 205.1080.

6-Methyl-2-(3,4,5-trimethyl-1*H*-pyrazol-1-yl)pyrimidin-4(3*H*)-one, TFA salt (6): Following procedure A, using 3-methylpentane-2,4-dione (42 µL, 0.36 mmol, 1.0 eq), after overnight stirring, pyrazole **6** (63 mg, 0.20 mmol, 57 %) was isolated as a white powder after purification by preparative HPLC (gradient from 70/30 A/D to 50/50 A/D in 18 min, t_R = 39 % D). RP-UPLC: t_R = 2.00 min (method A). ^{1}H NMR (300 MHz, $CDCl_3$): δ 6.08 (1H, s), 2.59 (3H, s), 2.28 (3H, s), 2.20 (3H, s), 1.94 (3H, s). ^{13}C NMR (100 MHz, $CDCl_3$): δ 165.6, 162.9, 152.2, 147.8, 139.6, 118.1, 108.4 (CH), 24.1 (CH_3), 13.3 (CH_3), 12.3 (CH_3), 8.0 (CH_3). HR-MS calcd for $C_{11}H_{15}N_4O$: *m/z* 219.1240, *m/z* found 219.1234.

2-(4-Ethyl-3,5-dimethyl-1*H*-pyrazol-1-yl)-6-methylpyrimidin-4(3*H*)-one, TFA salt (7): Following procedure A, using 3-ethylpentane-2,4-dione (74 µL, 0.55 mmol, 1.1 eq), after 2.5 h of stirring, pyrazole **7** (40 mg, 0.12 mmol, 24 %) was isolated as a white powder after purification by preparative HPLC (gradient from 65/35 A/D to 25/75 A/D in 18 min, t_R = 50 % D). RP-UPLC: t_R = 2.06 min (method A). ^{1}H NMR (300 MHz, DMSO): δ 6.38 (1H, s), 2.50 (3H, s), 2.41 – 2.33 (2H, q, *J* = 7.5 Hz), 2.23 (3H, s), 2.19 (3H, s), 1.05 – 1.00 (3H, t, *J* =7.8 Hz). ^{13}C NMR (75 MHz, DMSO): δ 174.0, 149.8, 138.0, 122.7, 117.6, 107.0, 22.8 (CH_2), 15.7 (CH_3), 14.7 (CH_3), 12.3 (CH_3), 11.8 (CH_3). HR-MS calcd for $C_{12}H_{17}N_4O$: *m/z* 233.1397, *m/z* found 233.1405.

2-(3,5-Dimethyl-4-phenyl-1*H*-pyrazol-1-yl)-6-methylpyrimidin-4(3*H*)-one (8): Following procedure A, 3-benzylpentane-2,4-dione (129 mg, 0.73 mmol, 1.1 eq) and ammonium acetate (102 mg, 1.3 mmol, 2.1 eq) were stirred overnight to afford pyrazole **8** (84 mg, 0.30 mmol, 46 %) as a white solid after filtration, washing with cold ethanol and drying *in vacuo*. RP-UPLC: t_R = 2.29 min (method A). ^{1}H NMR (300 MHz, $CDCl_3$): δ 7.40 – 7.15 (5H, m), 6.02 (1H, s) 2.57 (3H, s), 2.22 (3H, s), 2.16 (3H, s). ^{13}C NMR (100 MHz, $CDCl_3$): δ 165.4, 162.1, 151.0, 147.6, 140.0, 131.9, 129.9 (CH_{ar}), 128.8 (CH_{ar}), 127.6 (CH_{ar}), 125.3, 109.1 (CH), 24.2 (CH_3), 14.1 (CH_3), 12.9 (CH_3). HR-MS calcd for $C_{16}H_{17}N_4O$: *m/z* 281.1402, *m/z* found 281.1397.

2-(4-Benzyl-3,5-dimethyl-1*H*-pyrazol-1-yl)-6-methylpyrimidin-4(3*H*)-one, TFA salt (9): Following procedure A, 3-benzylpentane-2,4-dione (0.14 mL, 0.79 mmol, 1.1 eq) and ammonium acetate (127 mg, 1.7 mmol, 2.3 eq) were stirred overnight to afford pyrazole **9** (15 mg, 0.037 mmol, 5 %) as a white powder after purification by column chromatography on silica gel (gradient Hexane / Ethyl acetate) and by preparative HPLC (gradient from 65/35 A/D to 30/70 A/D in 18 min, t_R = 61 % D). RP-UPLC: t_r = 2.45 min (method A). ^{1}H NMR (300 MHz, $CDCl_3$): δ 7.26 – 7.04 (5H, m), 6.05 (1H, s), 3.72 (2H, s), 2.61 (3H, s), 2.24 (3H, s), 2.08 (3H, s). ^{13}C NMR (100 MHz, $CDCl_3$): δ 165.5, 162.5, 152.3, 147.7, 140.4, 139.3, 128.7 (CH_{ar}), 128.1

(CH_{ar}), 126.5 (CH_{ar}), 121.2, 108.7 (CH), 29.0 (CH_2), 24.1 (CH_3), 13.5 (CH_3), 12.5 (CH_3). HR-MS calcd for $C_{17}H_{19}N_4O$: *m/z* 295.1559, *m/z* found 295.1546.

2-(3-(*tert*-Butyl)-5-methyl-1*H*-pyrazol-1-yl)-6-methylpyrimidin-4(3*H*)-one, TFA salt (10): Following procedure A, using 5,5-dimethylhexane-2,4-dione (87 µL, 0.56 mmol, 1.1 eq), after 6.5 h of stirring, pyrazole **10** (57 mg, 0.23 mmol, 45 %) was isolated as a white powder after purification by preparative HPLC (gradient from 65/35 A/D to 25/75 A/D in 18 min, t_R = 52 % D). RP-UPLC: t_R = 2.13 min (method A). ^{1}H NMR (300 MHz, DMSO): δ 6.35 (1H, s), 6.14 (1H, s), 2.27 (3H, s), 2.017 (3H, s), 1.30 (9H, s). ^{13}C NMR (100 MHz, $CDCl_3$): δ 164.5, 162.2, 157.1, 151.2, 147.2, 110.3 (CH), 109.5 (CH), 33.5 (CH_3), 23.6 (CH_3), 13.7 (CH_3). HR-MS calcd for $C_{13}H_{19}N_4O$: *m/z* 247.1559, *m/z* found 247.1560.

6-Methyl-2-(5-methyl-3-phenyl-1*H*-pyrazol-1-yl)pyrimidin-4(3*H*)-one, TFA salt (11): Following procedure A, using 1-phenylbutane-1,3-dione (82 mg, 0.50 mmol, 1.0 eq), after 5 h of stirring, pyrazole **11** (20 mg, 0.075 mmol, 15 %) was isolated as a white powder after purification by preparative HPLC (gradient from 65/35 A/D to 20/80 A/D in 18 min, t_R = 50 % D). RP-UPLC: t_R = 1.97 min (method A). ^{1}H NMR (300 MHz, DMSO): δ 7.40 – 7.26 (5H, m), 6.51 (1H, s), 6.30 (1H, s), 2.28 (3H, s), 2.04 (3H, s). ^{13}C NMR (100 MHz, $CDCl_3$): δ 165.1, 162.2, 152.3, 146.7, 146.5, 130.9, 129.7 (CH_{ar}), 128.8 (CH_{ar}), 127.6 (CH_{ar}), 113.0 (CH), 109.4 (CH), 23.5 (CH_3), 13.8 (CH_3). HR-MS calcd for $C_{15}H_{15}N_4O$: *m/z* 267.1246, *m/z* found 267.1244.

6-Methyl-2-(3-methyl-5,6-dihydrocyclopenta[c]pyrazol-2(4*H*)-yl)pyrimidin-4(3*H*)-one, TFA salt (12): Following procedure A, 2-acetylcyclopentanone (70 µL, 0.58 mmol, 1.1 eq) and ammonium acetate (99 mg, 1.3 mmol, 2.4 eq) were stirred overnight to afford pyrazole **12** (150 mg, 0.43 mmol, 82 %) as a white powder after purification by preparative HPLC (gradient from 80/20 A/D to 45/55 A/D in 18 min, t_R = 6 % D). RP-UPLC: t_R = 1.34 min (method A). ^{1}H NMR (300 MHz, $CDCl_3$): δ 10.02 (1H, s), 5.93 (1H, s), 3.33 (1H, s), 2.53 – 2.49 (1H, m), 2.44

– 2.23 (3H, m), 2.19 – 1.90 (5H, m), 1.91 – 1.60 (2H, m), 1.53 – 1.34 (1H, m). ^{13}C NMR (100 MHz, $CDCl_3$): δ 182.4, 171.7, 163.5, 156.5, 149.4, 103.8, 100.4 (CH), 61.8 (CH or CH_3), 39.8 (CH_2), 29.3 (CH_2), 25.3 (CH_2), 19.0 (CH_3), 14.7 (CH_3). HR-MS calcd for $C_{12}H_{15}N_4O$: *m/z* 231.1240, *m/z* found 231.1237.

6-Methyl-2-(3-methyl-4,5,6,7-tetrahydro-2*H*-indazol-2-yl)pyrimidin-4(3*H*)-one and 6-methyl-2-(3-methyl-4,5,6,7-tetrahydro-1*H*-indazol-1-yl)pyrimidin-4(3*H*)-one, TFA salt (13): Following procedure A, 2-acetylcyclohexanone (67 µL, 0.51 mmol, 1.0 eq) and ammonium acetate (86 mg, 1.1 mmol, 2.2 eq) were stirred overnight to afford pyrazole **13** (63 mg, 0.26 mmol, 50 %) as a white powder and a brown mixture of **13** and **13a** (46 mg, 0.19 mmol, 37 %) after purification by preparative HPLC (gradient from 65/35 A/D to 30/70 A/D in 18 min, t_R = 55 % D). RP-UPLC: **13** t_R = 2.13 min (method A), **13a** t_R = 2.17 min (method A). ^{1}H NMR (300 MHz, $CDCl_3$): **13** δ 10.10 (1H, s), 6.09 (1H, s), 2.65 – 2.61 (2H, t, *J* = 6.0 Hz), 2.55 (3H, s), 2.46 – 2.42 (2H, t, *J* = 6.3 Hz), 2.28 (3H, s), 1.82 – 1.70 (4H, m); **13a** δ 9.84 (1H, s), 6.08 (1H, s), 3.08 – 3.04 (2H, t, *J* = 5.7 Hz), 2.37 – 2.33 (2H, t, *J* = 5.2 Hz), 2.27 (3H, s), 2.15 (3H, s), 1.80 – 1.68 (4H, m). ^{13}C NMR (100 MHz, $CDCl_3$): **13** δ 165.39, 163.51, 153.47, 147.96, 138.46, 119.75, 108.07 (CH), 23.87 (CH_3), 23.45 (CH_2), 22.86 (CH_2), 22.80 (CH_2), 20.17 (CH_2), 13.19 (CH_3). HR-MS calcd for $C_{13}H_{17}N_4O$: *m/z* 245.1402, *m/z* found 245.1391.

6-Methyl-2-(3-methyl-6,7-dihydropyrano[4,3-c]pyrazol-1(4*H*)-yl)pyrimidin-4(3*H*)-one and 6-methyl-2-(3-methyl-6,7-dihydropyrano[4,3-c]pyrazol-2(4*H*)-yl)pyrimidin-4(3*H*)-one, TFA salt (14): Following procedure A, **29** (90 mg, 0.63 mmol, 1.0 eq) and ammonium acetate (106 mg, 1.4 mmol, 2.2 eq) were stirred overnight to afford **14** as a mixture of two isomers (49 mg, 0.14 mmol, 21 %) as a brown powder after purification by preparative HPLC (gradient from 85/15 A/D to 55/45 A/D in 18 min, t_R = 30 % D). RP-UPLC: t_R = 1.60 min (method A). ^{1}H NMR (300 MHz, $CDCl_3$): **isomer A** δ 6.15 (1H, s), 4.65 (2H, s), 3.95 (2H, t, *J* = 5.7 Hz), 2.81 (2H, t, *J* = 5.7 Hz), 2.57 (3H, s), 2.31 (3H, s); **isomer B** δ: 6.13 (1H, s), 4.59

(2H, s), 3.94 – 3.89 (2H, m), 3.22 (2H, t, *J* = 5.6 Hz), 2.29 (3H, s), 2.20 (3H, s). ^{13}C NMR (100 MHz, $CDCl_3$): **isomer A** δ 165.3, 162.6, 149.8, 147.8, 136.6, 117.8, 109.0 (CH), 65.1 (CH_2), 62.8 (CH_2), 24.3 (CH_2), 24.1 (CH_3), 13.6 (CH_3); **isomer B** δ: 148.6, 147.1, 139.2, 119.2, 108.8 (CH), 64.0 (CH_2), 62.7 (CH_2), 26.7 (CH_2), 13.3 (CH_3), 12.3 (CH_3). HR-MS calcd for $C_{12}H_{15}N_4O_2$: *m/z* 247.1190, *m/z* found 247.1190.

6-Methyl-2-(3-methyl-4,5,6,7-tetrahydro-2*H*-pyrazolo[4,3-c]pyridin-2-yl)pyrimidin-4(3*H*)-one, TFA salt (15): Compound **30** (50 mg, 0.15 mmol, 1.0 eq) was dissolved in DCM (2 mL). TFA (0.11 mL, 1.5 mmol, 10 eq) was added. The solution was stirred at room temperature for 1 h. DCM was evaporated in *vacuo*. The residue was dissolved in DCM, stirred and concentrated twice. The residue was purified by preparative HPLC (gradient from 100 % A to 85/15 A/D in 20 min, t_R = 9 % D) to afford pyrazole **15** (39 mg, 0.08 mmol, 53 %) as a brown powder. RP-UPLC: t_R = 1.15 min (method A). ^{1}H NMR (300 MHz, MeOD): δ 6.19 (1H, s), 4.28 (2H, s), 3.58 (2H, t, *J* = 6.3 Hz), 3.09 (2H, t, *J* = 6.3 Hz), 2.62 (3H, s), 2.34 (3H, s). ^{13}C NMR (100 MHz, MeOD): δ 168.1, 165.7, 151.0, 147.9, 140.7, 112.7, 108.7 (CH), 43.2 (CH_2), 40.8 (CH_2), 22.9 (CH_3), 21.6 (CH_2), 13.5 (CH_3). HR-MS calcd for $C_{12}H_{16}N_5O$: *m/z* 246.1349, *m/z* found 246.1349.

2-(3,5-Diethyl-1*H*-pyrazol-1-yl)-6-methylpyrimidin-4(3*H*)-one, TFA salt (16): Following procedure A, using heptane-3,5-dione (80 µL, 0.60 mmol, 1.0 eq), after 3 h of stirring, pyrazole **16** (90 mg, 0.26 mmol, 43 %) was isolated as a white powder after purification by preparative HPLC (gradient from 60/40 A/D to 25/75 A/D in 18 min, t_R = 53 % D).

RP-UPLC: t_R = 2.17 min (method A). ^{1}H NMR (300 MHz, $CDCl_3$): δ 6.04 (1H, s), 6.01 (1H, s), 3.09 (2H, q, *J* = 7.4 Hz), 2.57 (2H, q, *J* = 7.6 Hz), 2.22 (3H, s), 1.27 – 1.18 (6H, m). ^{13}C NMR (75 MHz, $CDCl_3$): δ 165.1, 162.0, 157.5, 149.9, 147.4, 108.8 (CH), 108.4 (CH), 24.0 (CH3), 22.2 (CH_2), 21.6 (CH_2), 12.7 (CH_3), 12.6 (CH_3). HR-MS calcd for $C_{12}H_{17}N_4O$: *m/z* 233.1402, *m/z* found 233.1397.

2-(3-Ethyl-4,5,6,7-tetrahydro-2*H*-indazol-2-yl)-6-methylpyrimidin-4(3*H*)-one (17): Following procedure A, compound **33** (95 %, 82 mg, 0.51 mmol, 1.0 eq) and ammonium acetate (78 mg, 1.0 mmol, 2.0 eq) were stirred overnight to afford pyrazole **17** (90 mg, 0.26 mmol, 43 %) as a brown solid after precipitation in a mixture of A/D (1/1), washing with A and drying *in vacuo*. RP-UPLC: t_R = 2.33 min (method A). ^{1}H NMR (400 MHz, DMSO): δ 11.92 (1H, s), 6.13 (1H, s), 2.99 (2H, q, *J* = 7.3 Hz), 2.60 (2H, t, *J* = 5.8 Hz), 2.46 (2H, t, *J* = 5.9 Hz), 2.23 (3H, s), 1.79 – 1.66 (4H, m), 1.14 (3H, t, *J* = 7.4 Hz). ^{13}C NMR (100 MHz, DMSO): δ 164.9, 163.4, 151.6, 149.7, 142.7, 117.7, 107.3 (CH), 23.3 (CH_3), 23.0 (CH_2), 22.5 (CH_2), 22.5 (CH_2), 19.5 (CH_2), 19.0 (CH_2), 13.0 (CH_3). HR-MS calcd for $C_{14}H_{19}N_4O$: *m/z* 259.1553, *m/z* found 259.1548.

2-(4,5,5a,6,7,8-Hexahydrobenzo[cd]indazol-1(3*H*)-yl)-6-methylpyrimidin-4(3*H*)-one (18): Following procedure A, decalin-1,8-dione (83 mg, 0.50 mmol, 1.0 eq) and ammonium acetate (80 mg, 1.0 mmol, 2.0 eq) were stirred overnight to afford pyrazole **18** (54 mg, 0.2 mmol, 40 %) as a brown solid after filtration, washing with ethanol and drying *in vacuo*. RP-UPLC: t_R = 2.37 min (method A). ^{1}H NMR (300 MHz, $CDCl_3$): δ 6.07 (1H, s), 3.32 (1H, dd, *J* = 18.8, 6.3 Hz), 2.98 – 2.79 (2H, m), 2.68 – 2.43 (2H, m), 2.27 (3H, s), 2.17 – 2.06 (2H, m), 2.04 – 1.91 (2H, m), 1.87 – 1.64 (2H, m), 1.11 – 0.90 (2H, m). ^{13}C NMR (100 MHz, $CDCl_3$): δ 165.7, 162.9, 152.1, 148.1, 140.7, 124.8, 108.3 (CH), 32.9 (CH), 29.7 (CH_2), 29.4 (CH_2), 25.9 (CH_2), 24.1 (CH_3), 23.2 (CH_2), 22.7 (CH_2), 22.7 (CH_2). HR-MS calcd for $C_{15}H_{19}N_4O$: *m/z* 271.1553, *m/z* found 271.1551.

2-(3,5-Dimethyl-1*H*-pyrazol-1-yl)-5,6-dimethylpyrimidin-4(3*H*)-one, TFA salt (19): 3,5-Dimethylpyrazole-1-carboxamidine, nitric acid (112 mg, 0.56 mmol, 1.0 eq), ethyl 2-methyl-3-oxo-butanoate (80 µL, 0.56 mmol, 1.0 eq) and potassium carbonate (81 mg, 0.59 mmol, 1.1 eq) were dissolved in ethanol (5 mL). The mixture was stirred at 80 °C for two days. The mixture was filtered, and ethanol was evaporated under reduced pressure. The residue was

purified by preparative HPLC (gradient from 90/10 A/D to 40/60 A/D in 18 min, t_R = 45 % D) to afford pyrazolone **19** (15 mg, 0.045 mmol, 8 %) as a white powder. RP-UPLC: t_R = 1.88 min (method A). ^{1}H NMR (300 MHz, $CDCl_3$): δ 6.00 (1H, s), 2.65 (3H, s), 2.29 (6H, s), 2.23 (3H, s), 2.05 (3H, s). ^{13}C NMR (75 MHz, $CDCl_3$): δ 162.3, 159.7, 151.5, 144.8, 143.3, 116.8, 111.2 (CH), 22.1 (CH_3), 15.1 (CH_3), 13.7 (CH_3), 11.0 (CH_3). HR-MS calcd for $C_{11}H_{15}N_4O$: *m/z* 219.1240, *m/z* found 219.1237.

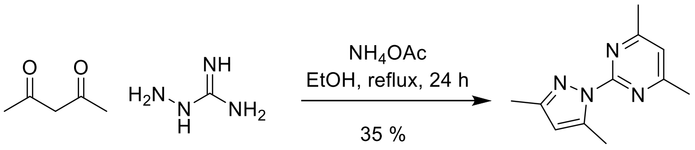

20

Scheme S4: Synthesis of **20**.

2-(3,5-Dimethyl-1*H*-pyrazol-1-yl)-4,6-dimethylpyrimidine, TFA salt (20): 1-Aminoguanidine (134 mg, 0.55 mmol, 1.0 eq) was suspended in ethanol (5 mL). Ammonium acetate (168 mg, 2.2 mmol, 4.0 eq) and pentane-2,4-dione (0.22 mL, 2.2 mmol, 4.0 eq) were added. The mixture was stirred at 80 °C overnight. Ethanol (5 mL), ammonium acetate (80 mg, 1.0 mmol, 1.9 eq) and pentane-2,4-dione (0.11 mL, 1.1 mmol, 2.0 eq) were added. The mixture was stirred at 80 °C overnight. Ethanol (5 mL) and ammonium acetate (85 mg, 1.1 mmol, 2.0 eq) were added. The mixture was stirred at 80 °C overnight. Ethanol was evaporated under reduced pressure, and the residue was purified by preparative HPLC (gradient from 70/30 A/D to 40/60 A/D in 18 min, t_R = 38 % D) to afford pyrazole **20** (59 mg, 0.19 mmol, 35 %) as a yellow oil. RP-UPLC: t_R = 1.82 min (method A). ^{1}H NMR (300 MHz, DMSO): δ 7.18 (1H, s), 6.11 (1H, s), 2.53 (3H, s), 2.45 (6H, s), 2.19 (3H, s). ^{13}C NMR (75 MHz, DMSO): δ 168.4, 156.6, 149.2, 141.8, 117.5 (CH), 109.4 (CH), 23.6 (CH_3), 14.5 (CH_3), 13.5 (CH_3). HR-MS calcd for $C_{11}H_{15}N_4$: *m/z* 203.1297, *m/z* found 203.1289.

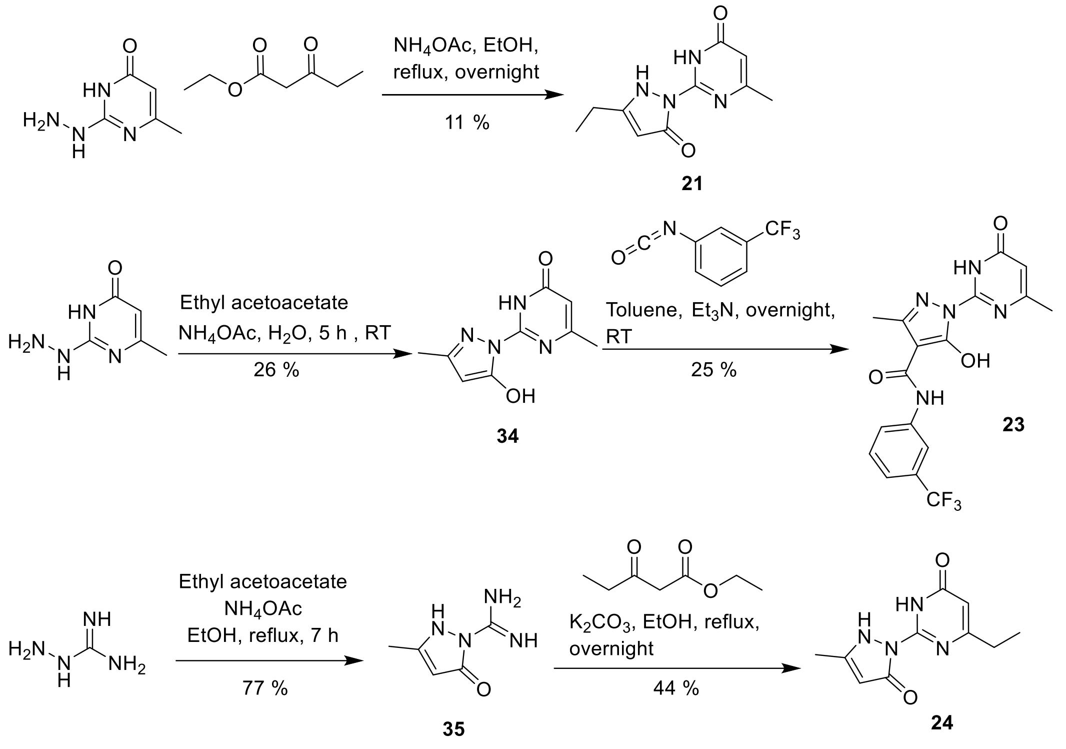

Scheme S5: Synthesis of compounds **21-25**.

2-(3-Ethyl-5-oxo-2,5-dihydro-1*H*-pyrazol-1-yl)-6-methylpyrimidin-4(3*H*)-one, TFA salt (21): Following procedure A, using ethyl 3-oxopentanoate (51 µL, 0.36 mmol, 1.0 eq), after overnight stirring, pyrazolone **21** (13 mg, 0.040 mmol, 11 %) was isolated as a white powder after purification by preparative HPLC (gradient from 85/15 A/D to 65/35 A/D in 18 min, t_R = 20 % D). RP-UPLC: t_R = 1.46 min (method A). ^{1}H NMR (300 MHz, $CDCl_3$): δ 10.55 (2H, s), 6.03 (1H, s), 5.40 (1H, s), 2.57 (2H, q, *J* = 7.6 Hz), 2.26 (3H, s), 1.23 (3H, t, *J* = 7.6 Hz). ^{13}C NMR (100 MHz, $CDCl_3$): δ 162.3, 161.8, 159.9, 148.5, 108.7 (CH), 89.7, 23.3 (CH_3), 22.0 (CH_2), 12.3 (CH_3). HR-MS calcd for $C_{10}H_{13}N_4O_2$: *m/z* 221.1033, *m/z* found 221.1030.

2-(4-Benzyl-3-methyl-5-oxo-2,5-dihydro-1*H*-pyrazol-1-yl)-6-methylpyrimidin-4(3*H*)-one, TFA salt (22): Following procedure A, ethyl 2-benzyl-3-oxo-butanoate (0.12 mL, 0.55 mmol, 1.1 eq) and ammonium acetate (80 mg, 1.0 mmol, 2.1 eq) were stirred overnight to afford pyrazolone **22 (**149 mg, 0.36 mmol, 72 %) as a pink powder after purification by preparative HPLC (gradient from 70/30 A/D to 40/60 A/D in 18 min, t_R = 41 % D). RP-UPLC: t_R = 1.83

min (method A). ^{1}H NMR (300 MHz, DMSO): δ 7.35 – 7.05 (5H, m), 6.02 (1H, s), 3.55 (2H, s), 2.23 (3H, s), 2.17 (3H, s). ^{13}C NMR (75 MHz, DMSO): δ 163.3, 163.1, 161.3, 151.2, 147.2, 140.2, 128.3 (CH_{ar}), 128.0 (CH_{ar}), 125.9 (CH), 107.6 (CH), 102.4, 26.7 (CH_2), 23.1 (CH_3), 10.9 (CH_3). HR-MS calcd for $C_{16}H_{17}N_4O_2$: *m/z* 297.1352, *m/z* found 297.1335.

5-Hydroxy-3-methyl-1-(4-methyl-6-oxo-1,6-dihydropyrimidin-2-yl)-*N*-(3-(trifluoromethyl)phenyl)-1*H*-pyrazole-4-carboxamide (23):[9] Compound **34** (185 mg, 0.90 mmol, 1.0 eq) was suspended in toluene (5 mL) before the addition of triethyl amine (242 µL, 1.8 mmol, 2.0 eq) and 1-isocyanato-3-(trifluoromethyl)benzene (126 µL, 0.90 mmol, 1.0 eq). The mixture was stirred at room temperature overnight. The precipitate was filtered and dissolved in water / acetonitrile. Acetonitrile was evaporated under reduced pressure and the aqueous phase was acidified with HCl 1M. The precipitate was filtered to afford pyrazolone **23** (85 mg, 0.22 mmol, 25 %) as a yellow solid. ^{1}H NMR (400 MHz, DMSO): δ 10.09 (1H, s), 8.27 (1H, d, *J* = 2.0 Hz), 7.68 (1H, dd, *J* = 8.1, 2.1 Hz), 7.53 (1H, t, *J* = 7.9 Hz), 7.36 (1H, d, *J* = 7.7 Hz), 6.18 (1H, d, *J* = 1.1 Hz), 2.47 (3H, s), 2.31 (3H, s). ^{13}C NMR (100 MHz, DMSO): δ 164.5, 161.6, 161.0, 158.6, 156.2, 147.7, 139.9, 130.0 (CH), 129.6 (d, *J* = 31.4 Hz), 125.5, 122.8, 122.4 (CH), 118.9 (CH), 114.9 (d, *J* = 4 Hz, CH), 107.0 (CH), 94.3, 21.0 (CH_3), 14.2 (CH_3). HR-MS calcd for $C_{17}H_{15}F_3N_5O$: *m/z* 394.1122, *m/z* found 394.1112.

6-Ethyl-2-(3-methyl-5-oxo-2,5-dihydro-1*H*-pyrazol-1-yl)pyrimidin-4(3*H*)-one, TFA salt (24): Compound **35** (70 mg, 0.50 mmol, 1.0 eq), ethyl 3-oxopentanoate (70 µL, 0.50 mmol, 1.0 eq) and potassium carbonate (81 mg, 0.59 mmol, 1.2 eq) were stirred in ethanol (5 mL) at 80 °C overnight. Ethanol was evaporated under reduced pressure, and the residue was purified by preparative HPLC (gradient from 90/10 A/D to 70/30 AD, t_R = 19 % D) to afford pyrazolone **24** (73 mg, 0.22 mmol, 44%) a white powder. RP-UPLC: t_R = 1.49 min (method A). ^{1}H NMR (400 MHz, DMSO): δ 8.49 (1H, s), 5.29 (1H, s), 4.23 (2H, q, *J* = 7.1 Hz), 2.22 (3H, s), 1.26 (3H, t, *J* = 7.1 Hz). ^{13}C NMR (100 MHz, MeOD): δ 169.9, 166.3, 165.8, 155.0, 148.7, 107.0

(CH), 92.6, 92.3, 92.0, 30.6 (CH_2), 12.5 (CH_3), 12.3 (CH_3). HR-MS calcd for $C_{10}H_{13}N_4O_2$: *m/z* 221.1033, *m/z* found 221.1029.

Ethyl 2-(3-methyl-5-oxo-2,5-dihydro-1*H*-pyrazol-1-yl)-6-oxo-1,6-dihydropyrimidine-5-carboxylate, TFA salt (25): Compound **35** (70 mg, 0.50 mmol, 1.4 eq), diethyl 2-(ethoxymethylene)propanedioate (71 µL, 0.35 mmol, 1.0 eq) and potassium carbonate (83 mg, 0.60 mmol, 1.7 eq) were stirred in ethanol (5 mL) at 80 °C for 7.5 h. The precipitate was filtered, washed with ethanol and purified by preparative HPLC (gradient from 90/10 A/D to 70/30 AD, t_R = 18 % D) to afford pyrazolone **25** (76 mg, 0.20 mmol, 57 %) as an off-white powder. RP-UPLC: t_R = 1.49 min (method A). ^{1}H NMR (400 MHz, DMSO): δ 8.48 (1H, s), 5.29 (1H, s), 4.22 (2H, q, *J* = 7.1 Hz), 2.21 (3H, s), 1.26 (3H, t, *J* = 7.1 Hz). ^{13}C NMR (100 MHz, DMSO): δ 164.5, 163.2, 154.8, 149.0, 112.0, 90.9 (CH), 60.3 (CH_2), 14.2 (CH_3), 12.3 (CH_3). HR-MS calcd for $C_{10}H_{13}N_4O_4$: *m/z* 265.0931, *m/z* found 265.0929.

4,6-Dimethyldibenzo[b,d]furan (26)[8]. In a three-neck flask, dibenzofuran (2.0 g, 11.9 mmol, 1.0 eq) was dissolved in dry diethyl ether (100 mL) under argon atmosphere. The temperature was cooled down to – 78 °C before the addition of TMEDA (4.3 mL, 29.5 mmol, 2.6 eq). *sec* BuLi (21.2 mL, 1.4 M, 29.7 mmol, 2.5 eq) was added dropwise. The mixture was allowed to warm to room temperature and was stirred 16 hours. Iodomethane (3.80 mL, 61.0 mmol, 5.0 eq) was added. The reaction mixture was stirred at room temperature for 20 hours and NH_4Cl *sat* (70 mL) was added. The product was extracted with diethyl ether (3 × 100 mL). The organic layers were combined, dried over Na_2SO_4, filtered and concentrated. The residue was recrystallized from methanol to afford **26** (846 mg, 4.3 mmol, 36 %) as a white solid. ^{1}H NMR (300 MHz, $CDCl_3$): δ 7.77 (2H, dd, *J* = 6.4, 2.6 Hz), 7.25 – 7.20 (2H, m), 2.63 (6H, s). ^{13}C NMR (75 MHz, $CDCl_3$): δ 155.1, 128.0, 124.2, 122.6, 122.0, 118.2, 15.4.

4,6-Bis(bromomethyl)dibenzo[b,d]furan (27)[8]**.** Compound **26** (74 mg, 0.38 mmol, 1.0 eq) and NBS (152 mg, 0.85 mmol, 2.2 eq) were dissolved in CCl_4 (28 mL) under argon. Benzoyl peroxide (1 spatula) was added. The mixture was stirred at 60 °C overnight before the addition of benzoyl peroxide (1 spatula). The mixture was stirred at 60 °C during 2.5 h and then cooled down to 0 °C. The white precipitate was filtered, and the solvent was evaporated under reduced pressure. The residue was recrystallized from Hexanes / Ethyl acetate (50:50) to afford **27** (43 mg, 0.12 mmol, 32 %) as an off-white solid. ^{1}H NMR (400 MHz, $CDCl_3$): δ 7.91 (2H, d, *J* = 7.5 Hz), 7.52 (2H, d, *J* = 7.4 H), 7.35 (2H, t, *J* = 7.6 Hz), 4.91 (4H, s). ^{13}C NMR (100 MHz, $CDCl_3$): δ 154.0, 128.5, 124.7, 123.5, 122.3, 121.4, 26.8.

3-Methyl-1-(pyridin-2-yl)-1*H*-pyrazol-5-ol (28).[10] 2-Pyridylhydrazine (119 mg, 1.1 mmol, 1.0 eq) and pentane-2,4-dione (110 µL, 1.1 mmol, 1.0 eq) were dissolved in acetic acid (6 mL). The solution was stirred at reflux for 3 hours. The solvent was removed by co-evaporation with toluene. The residue was dissolved in water and the product was extracted with ethyl acetate. The organic layer was washed with sat. $NaHCO_3$ and brine, and then dried over Na_2SO_4 and concentrated. The residue was purified by column chromatography on silica gel (gradient DCM/MeOH) to afford **28** (55 mg, 1.1 mmol, 29 %) as an off-white solid. RP-UPLC: t_R = 1.56 min (method A). ^{1}H NMR (300 MHz, $CDCl_3$): δ 8.25 (1H, d, *J* = 4.3 Hz), 7.97 (1H, d, *J* = 8.5 Hz), 7.87 (1H, td, *J* = 8.6, 8.0, 1.8 Hz), 7.14 (1H, ddd, *J* = 7.2, 5.2, 1.0 Hz), 5.44 (1H, s), 2.28 (3H, s). ^{13}C NMR (100 MHz, $CDCl_3$): δ 157.6, 153.9, 151.8, 145.2, 140.2, 119.9, 112.2, 89.1, 14.5. HR-MS calcd. for $C_9H_{10}N_3O$: *m/z* 176.0818, *m/z* found 176.0819.

1-(4-hydroxy-5,6-dihydro-2*H*-pyran-3-yl)ethan-1-one (29).[11]A mixture of tetrahydropyran-4-one (1.0 g, 10 mmol, 1.0 eq) and pyrrolidine (1.7 mL, 20 mmol, 2.0 eq) in dry toluene (5 mL) was heated at 92 °C for 3 h. The mixture was cooled down to room temperature and dried *in vacuo*. Dioxane (3 mL) and acetic anhydride (2.2 mL, 23 mmol, 2.3 eq) were added. The mixture was stirred at room temperature overnight. Water (1.2 mL) was added and the solution

was stirred at reflux for one hour. The solution was cooled down to room temperature and concentrated. The oily residue was dissolved in water and extracted twice with EtOAc. The combined organic layers were washed with 5 % HCl, dried over Na_2SO_4 and concentrated. The residue was purified by column chromatography on silica gel (gradient EtOAc/Hexane) to afford **29** (90 mg, 0.63 mmol, 6 %) as a yellow oil. RP-UPLC: t_R = 1.31 min (method A). ^{1}H NMR (300 MHz, $CDCl_3$): δ 15.5 (1H, s), 4.40 (2H, t, *J* = 1.0 Hz), 3.87 (2H, t, *J* = 5.8 Hz), 2.46 (2H, t, *J* = 5.8 Hz), 2.03 (3H, s). ^{13}C NMR (100 MHz, $CDCl_3$): δ 194.4, 180.3, 106.6, 64.6, 64.1, 31.3, 24.0. HR-MS calcd. for $C_7H_{11}O_3$: *m/z* 143.0703, *m/z* found 143.0703.

***tert*-Butyl 3-methyl-2-(4-methyl-6-oxo-1,6-dihydropyrimidin-2-yl)-2,4,6,7-tetrahydro-5*H*-pyrazolo[4,3-c]pyridine-5-carboxylate (30).** Compound **31** (60 %, 208 mg, 0.5 mmol, 1.0 eq), 2-hydrazino-4-methyl-1H-pyrimidin-6-one (71 mg, 0.5 mmol, 1.0 eq) and ammonium acetate (90 mg, 1.2 mmol, 2.3 eq) were dissolved in ethanol (5 mL). The mixture was stirred at 80 °C overnight. The solution was cooled down to room temperature and the solvent was evaporated under reduced pressure. The residue was purified by column chromatography (gradient DCM/MeOH), and then dissolved in a mixture of A and D (1/1). The off-white precipitate was filtered to afford **30** (50 mg, 0.15 mmol, 28 %).^{1}H NMR (300 MHz, $CDCl_3$) δ 6.11 (1H, s), 4.41 (2H, s), 3.70 (2H, t, *J* = 5.6 Hz), 2.75 (2H, t, *J* = 5.8 Hz), 2.61 (3H, s), 2.29 (3H, s), 1.50 (9H, s).

***tert*-Butyl 5-acetyl-4-hydroxy-3,6-dihydropyridine-1(2*H*)-carboxylate (31).**[11] A mixture of compound **32** (1.2 g, 5.9 mmol, 1.0 eq) and pyrrolidine (1.0 mL, 12 mmol, 2.0 eq) in dry toluene (5 mL) was heated at 92 °C for 3 h. The mixture was cooled down to room temperature and dried *in vacuo*. Dioxane (3 mL) and acetic anhydride (1.25 mL, 13.2 mmol, 2.2 eq) were added. The mixture was stirred overnight at room temperature. Water (1.2 mL) was added and the solution was stirred at reflux for one hour. The solution was cooled down to room temperature

and concentrated. The oily residue was dissolved in water and extracted twice with EtOAc. The combined organic layers were washed with 5 % HCl, dried over Na_2SO_4 and concentrated. The residue was purified by column chromatography on silica gel (gradient EtOAc/Hexane) to afford **31** (966 mg, 3.3 mmol, 56 %, purity LCMS 83 %) as a yellow liquid. RP-UPLC: t_R = 1.94 min, 2.23 min (method A). ^{1}H NMR (400 MHz, MeOD): δ 4.20 (2H, s), 3.59 (2H, t, *J* = 6.0 Hz), 2.42 (2H, tt, *J* = 6.0, 1.0 Hz), 2.14 (3H, s), 1.48 (9H, s). ^{13}C NMR (100 MHz, MeOD): δ 210.0, 197.9, 180.6, 156.3, 106.4, 81.7, 42.4 (CH_2), 41.2 (CH_2), 31.6 (CH_2), 28.6 (CH_3), 24.7 (CH_3). HR-MS calcd. for $C_{12}H_{20}NO_4$: *m/z* 242.1387, *m/z* found 242.1389.

***tert*-Butyl 4-oxopiperidine-1-carboxylate (32).** Triethylamine (0.84 mL, 6.0 mmol, 1.0 eq) was added drop wise to a mixture of piperidin-4-one, hydrochloride (838 mg, 6.2 mmol, 1.0 eq) and di-*tert*-butyl dicarbonate (2.06 mL, 9.0 mmol, 1.5 eq) in DCM (10 mL) at 0 °C. The resulting mixture was stirred overnight at room temperature. Water was added and the two phases were separated. The organic layer was washed with brine, dried over Na_2SO_4 and concentrated. Silica was added until all the solution was adsorbed. The silica was then filtered, washed with DCM. The solvents were evaporated, and the residue was dried *in vacuo* to afford **32** (958 mg, 4.8 mmol, 71 %) as an off-white solid. ^{1}H NMR (300 MHz, $CDCl_3$): δ 3.72 (4H, t, J = 6.2 Hz), 2.44 (4H, t, J = 6.3 Hz), 1.49 (9H, s). ^{13}C NMR (100 MHz, $CDCl_3$): δ 207.8, 154.5, 80.5, 43.1, 41.2, 28.4. HR-MS calcd. for $C_{10}H_{17}NO_3Na$: *m/z* 222.1102, *m/z* found 222.1101.

2-propionylcyclohexan-1-one (33).[11] A mixture of cyclohexanone (1.0 mL, 9.7 mmol, 1.0 eq) and pyrrolidine (1.7 mL, 20 mmol, 2.0 eq) in dry toluene (5 mL) was heated at 92 °C for 3 h. The mixture was cooled down to room temperature and dried *in vacuo*. Dioxane (4 mL) and propionic anhydride (2.8 mL, 22 mmol, 2.3 eq) were added. The mixture was stirred overnight at room temperature. Water (3 mL) was added at 0 °C. The solution was stirred at reflux for

one hour. The solution was cooled down to room temperature and concentrated. The oily residue was dissolved in water and extracted twice with EtOAc. The combined organic layers were washed with 5 % HCl, dried over Na_2SO_4 and concentrated. The residue was purified by column chromatography on silica gel (Hexane) to afford **33** (610 mg, 3.8 mmol, 39 %, purity 95 %) as a pale yellow oil. RP-UPLC: t_R = 3.66 min (method D). ^{1}H NMR (400 MHz, MeOD): δ 2.48 (2H, q, *J* = 7.3 Hz), 2.37 – 2.27 (4H, m), 1.73 – 1.65 (4H, m), 1.08 (3H, t, *J* = 7.3 Hz).^{13}C NMR (100 MHz, MeOD): δ 204.8, 180.6, 107.7, 31.5, 31.4, 24.7, 23.9, 22.7, 8.4. HR-MS calcd. for $C_9H_{14}O_2Na$: *m/z* 177.0886, *m/z* found 177.0880.

2-(5-hydroxy-3-methyl-1*H*-pyrazol-1-yl)-6-methylpyrimidin-4(3*H*)-one, TFA salt (34).[7] 2-Hydrazino-4-methyl-1*H*-pyrimidin-6-one (158 mg, 1.1 mmol, 1.0 eq) was suspended in water (6 mL). Sodium acetate (108 mg, 1.3 mmol, 1.2 eq) was sequentially added before the addition dropwise of ethyl 3-oxobutanoate (150 µL, 1.2 mmol, 1.1 eq). The white mixture was stirred vigorously at room temperature for 5 h. The residue was purified by preparative HPLC (gradient from 85/15 A/D to 65/35 A/D in 18 min, t_R = 15 % D) to afford **34** (93 mg, 0.29 mmol, 26 %) as a yellow solid. RP-UPLC: t_R = 1.26 min (method A). ^{1}H NMR (400 MHz, $CDCl_3$): δ 6.03 (1H, s), 5.40 (1H, s), 2.28 (3H, s), 2.24 (3H, s). ^{13}C NMR (100 MHz, $CDCl_3$): δ 161.9, 159.2, 154.3, 148.5, 108.7 (CH), 91.0 (CH), 23.2 (CH_3), 14.2 (CH_3). HR-MS calcd. for $C_9H_{11}N_4O_2$: *m/z* 207.0877, *m/z* found 207.0877.

3-methyl-5-oxo-2,5-dihydro-1*H*-pyrazole-1-carboximidamide (35). 1-Aminoguanidine, bicarbonate (1.6 g, 12 mmol, 1.5 eq), ethyl 3-oxobutanoate (1.0 g, 7.9 mmol, 1.0 eq) and ammonium acetate (1.3 g, 17 mmol, 2.2 eq) were dissolved in ethanol (40 mL). The solution was stirred at 80 °C for 7 h. The solution was cooled down to room temperature and the solvent was removed. Water was added and the product was let to crystallize overnight. Crystals were filtered, washed with water and dried *in vacuo* to afford **35** (850 mg, 6.1 mmol, 77 %) as a

yellow solid. RP-UPLC: t_R = 0.98 min (method A). ^{1}H NMR (300 MHz, DMSO): δ 7.73 (3H, s), 4.42 (1H, s), 1.95 (3H, s). ^{13}C NMR (75 MHz, DMSO): δ 167.6, 156.0, 153.0, 82.2 (CH), 15.0 (CH_3). HR-MS calcd. for $C_5H_9N_4O$: *m/z* 141.0771, *m/z* found 141.0768.

Xray deposition

CCDC 1976862, 1976832 and 1976759 contain the supplementary crystallographic data for this paper. These data can be obtained free of charge from The Cambridge Crystallographic Data Centre via www.ccdc.cam.ac.uk/structures

HPLC purity

Table S3: Purity of the final compounds.

Compound	Retention time	Purity [%]	HPLC method[a]
**1**	2.79 min	99	B
**3**	ND[b]	ND[b]	ND[b]
**5**	1.71 min	99	A
**6**	2.00 min	99	A
**7**	2.06 min	99	A
**8**	2.29 min	99	A
**9**	2.45 min	99	A
**10**	2.13 min	99	A
**11**	1.97 min	99	A
**12**	1.34 min	95	A
**13**	2.13 min	99	A
**14**	1.60 min	99	A
**15**	1.15 min	99	A
**16**	2.17 min	99	A
**17**	2.33 min	98	A
**18**	2.37 min	96	A
**19**	1.88 min	99	A
**20**	1.82 min	99	A
**21**	1.46 min	98	A
**22**	1.83 min	99	A
**23**	ND[b]	ND[b]	ND[b]
**24**	1.49 min	99	A
**25**	1.49 min	95	A

[a]Method A: in 2.2 min from 100 % A to 10 0% D, then staying at 100 % D. Method B: in 7.5 min from 10 0% A to 100 % D, then staying at 100 % D. [b]ND = Not Determined, the compound was not soluble in water and acetonitrile.

Reference

(1) Montalbetti, N.; Simonin, A.; Simonin, C.; Awale, M.; Reymond, J.-L.; Hediger, M. A. Discovery and Characterization of a Novel Non-Competitive Inhibitor of the Divalent Metal Transporter DMT1/SLC11A2. *Biochem Pharmacol* **2015**, *96* (3), 216–224. https://doi.org/10.1016/j.bcp.2015.05.002.

(2) Montalbetti, N.; Simonin, A.; Dalghi, M. G.; Kovacs, G.; Hediger, M. A. Development and Validation of a Fast and Homogeneous Cell-Based Fluorescence Screening Assay for Divalent Metal Transporter 1 (DMT1/SLC11A2) Using the FLIPR Tetra. *J Biomol Screen* **2014**, *19* (6), 900–908. https://doi.org/10.1177/1087057114521663.

(3) Mackenzie, B.; Ujwal, M. L.; Chang, M.-H.; Romero, M. F.; Hediger, M. A. Divalent Metal-Ion Transporter DMT1 Mediates Both H+ -Coupled Fe2+ Transport and Uncoupled Fluxes. *Pflüg. Arch.* **2006**, *451* (4), 544–558. https://doi.org/10.1007/s00424-005-1494-3.

(4) Mackenzie, B.; Takanaga, H.; Hubert, N.; Rolfs, A.; Hediger, M. A. Functional Properties of Multiple Isoforms of Human Divalent Metal-Ion Transporter 1 (DMT1). *Biochem. J.* **2007**, *403* (1), 59–69. https://doi.org/10.1042/BJ20061290.

(5) Pujol-Giménez, J.; Hediger, M. A.; Gyimesi, G. A Novel Proton Transfer Mechanism in the SLC11 Family of Divalent Metal Ion Transporters. *Sci. Rep.* **2017**, *7* (1), 1–17. https://doi.org/10.1038/s41598-017-06446-y.

(6) Cadieux, J. A.; Zhang, Z.; Mattice, M.; Brownlie-Cutts, A.; Fu, J.; Ratkay, L. G.; Kwan, R.; Thompson, J.; Sanghara, J.; Zhong, J.; Goldberg, Y. P. Synthesis and Biological Evaluation of Substituted Pyrazoles as Blockers of Divalent Metal Transporter 1 (DMT1). *Bioorg Med Chem Lett* **2012**, *22* (1), 90–95. https://doi.org/10.1016/j.bmcl.2011.11.069.

(7) Erkin, A. V.; Krutikov, V. I. Formation, Structure and Heterocyclization of Aminoguanidine and Ethyl Acetoacetate Condensation Products. *Russ. J. Gen. Chem.* **2009**, *79* (6), 1204–1209. https://doi.org/10.1134/S1070363209060309.

(8) Chafeev, M.; Chakka, N.; Cadieux, J.-J.; Fu, J.; Kamboj, R.; Kodumuru, V.; Langille, J.; Liu, S.; Sun, J.; Sviridov, S.; Zhang, Z. Tricyclic Compounds Useful in Treating Iron Disorders. WO2008109840A1, September 12, 2008.

(9) CADIEUX, J.-J.; Fu, J.; Sadalapure, K.; Kamboj, R.; Zhang, Z. Pyrazole and Pyrrole Compounds Useful in Treating Iron Disorders. WO2008121861A2, 2008.

(10) Orrego-Hernández, J.; Cobo, J.; Portilla, J. Chemoselective Synthesis of 5-Alkylamino-1 *H* -Pyrazole-4-Carbaldehydes by Cesium- and Copper-Mediated Amination: Synthesis of 5-Alkylamino-1 *H* -Pyrazole-4-Carbaldehydes. *Eur. J. Org. Chem.* **2015**, *2015* (23), 5064–5069. https://doi.org/10.1002/ejoc.201500505.

(11) ROYCHOWDHURY, A.; SHARMA, R.; GUPTE, A.; KANDRE, S.; GADEKAR, P., Keshavrao; CHAVAN, S.; JADHAV, R., Dnyandev; THAKRE, G., Amrutrao; BAJAJ, K.; JANRAO, R., Ashok; DEHADE, A.; GAIKWAD, N.; KADAM, K.; MORE, T., Sitaram; GUHA, T.; SEELABOYINA, B.-A. 31/4704 (2006. 01) A. 37/06 (2006. 01) masree; SABLE, V., Vasant. EZH2 INHIBITORS AND USES THEREOF. WO2015110999A1, July 30, 2015.

^{1}H-NMR and ^{13}C-NMR Spectra

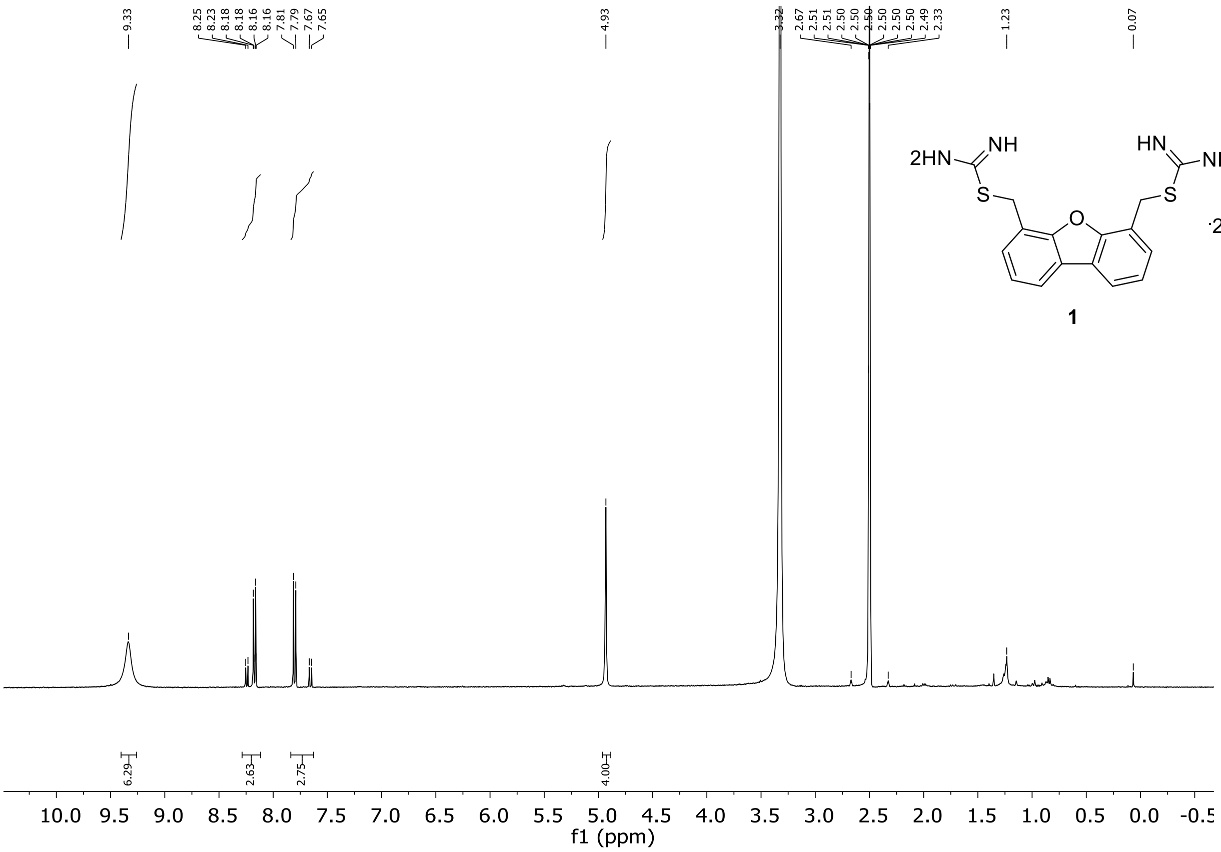

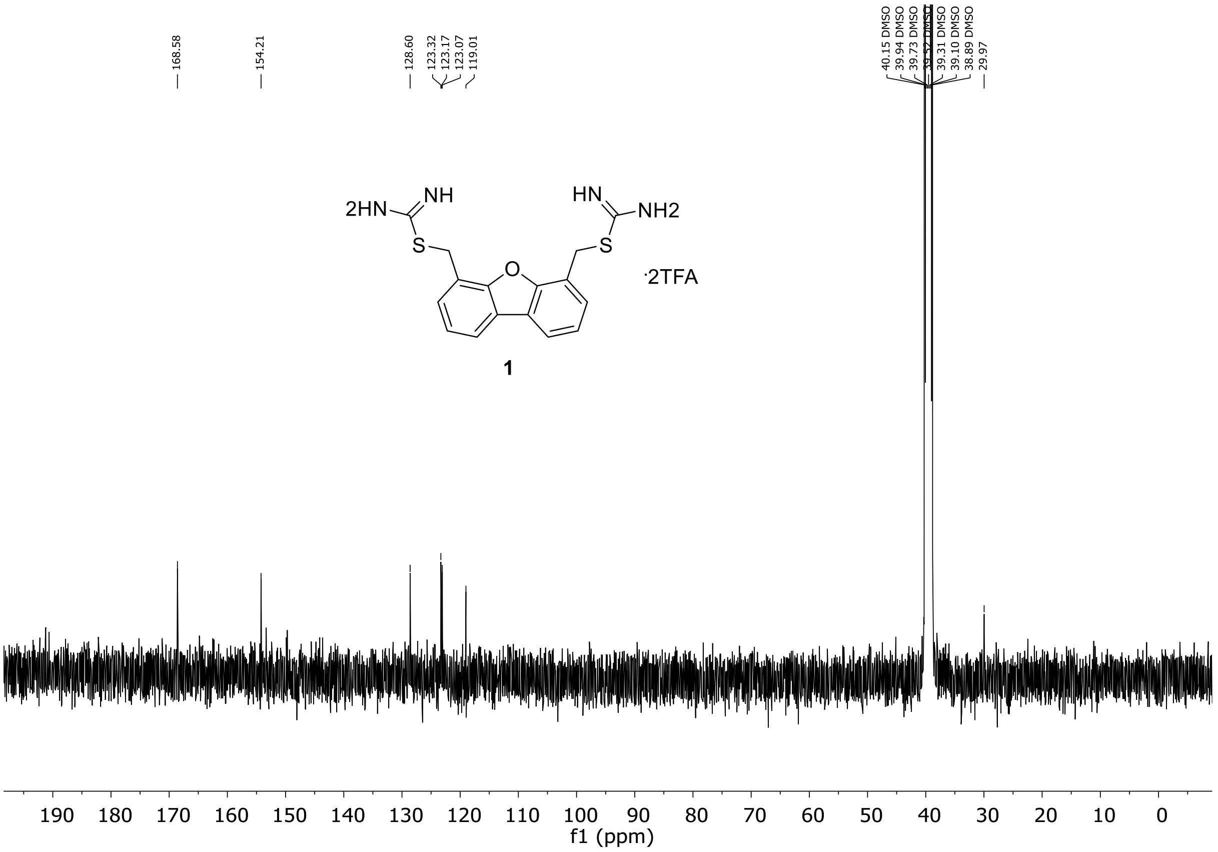

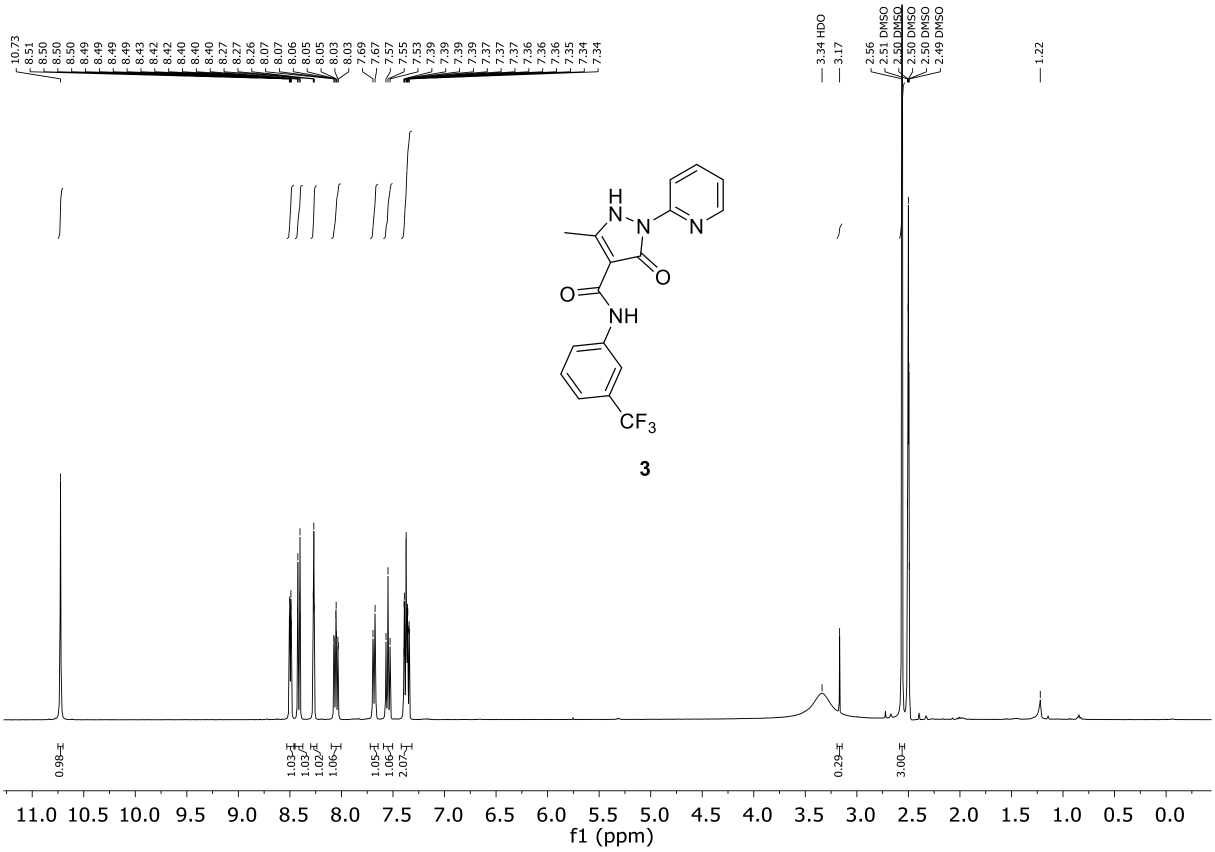
10.73
8.51
8.50
8.50
8.49
8.49
8.49
8.43
8.42
8.42
8.40
8.40
8.27
8.27
8.26
8.07
8.06
8.05
8.05
8.03
8.03
7.69
7.67
7.57
7.55
7.53
7.39
7.39
7.39
7.39
7.37
7.37
7.36
7.36
7.35
7.34
3.34 HDO
3.17
2.56
2.51 DMSO
2.50 DMSO
2.50 DMSO
2.50 DMSO
2.49 DMSO
1.22
H
N
N
N
O
O
NH
CF3
3
0.98
1.03
1.03
1.02
1.06
1.05
1.06
2.07
0.29
3.00
11.0 10.5 10.0 9.5 9.0 8.5 8.0 7.5 7.0 6.5 6.0 5.5 5.0 4.5 4.0 3.5 3.0 2.5 2.0 1.5 1.0 0.5 0.0
f1 (ppm)

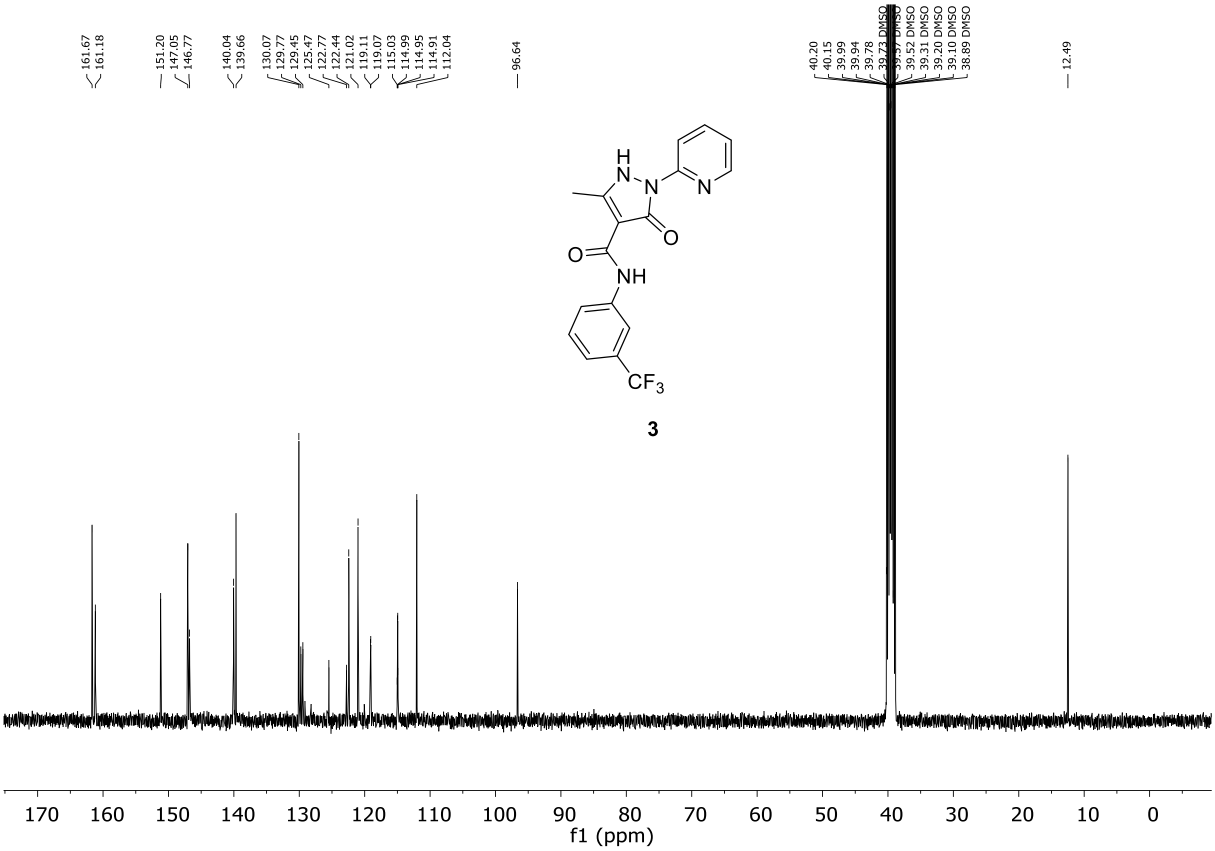
161.67
161.18
151.20
147.05
146.77
140.04
139.66
130.07
129.77
129.45
125.47
122.77
122.44
121.02
119.11
119.07
115.03
114.99
114.95
114.91
112.04
96.64
40.20
40.15
39.99
39.94
39.78
39.73 DMSO
39.57 DMSO
39.52 DMSO
39.31 DMSO
39.20 DMSO
39.10 DMSO
38.89 DMSO
12.49
H
N
N
N
O
O
NH
CF3
3
170 160 150 140 130 120 110 100 90 80 70 60 50 40 30 20 10 0
f1 (ppm)

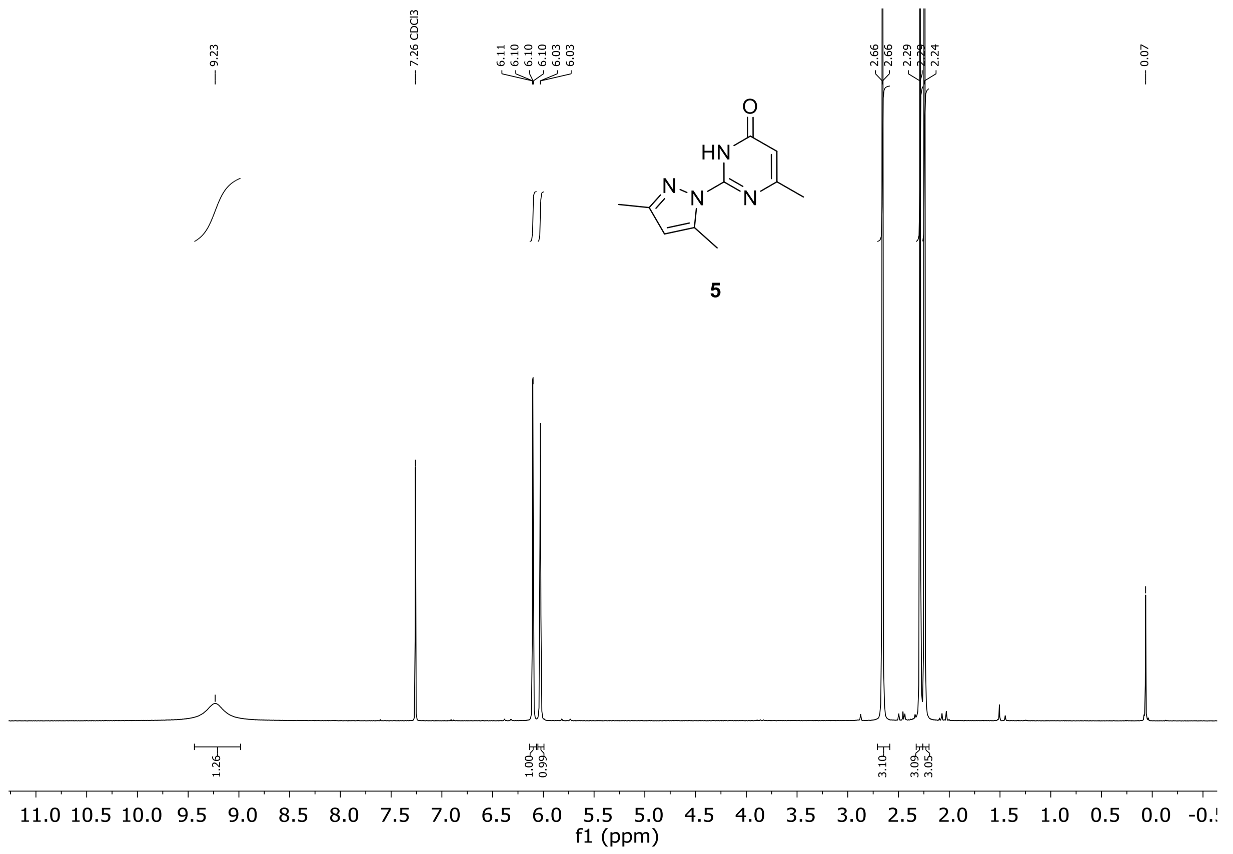
9.23
7.26 CDCl3
6.11
6.10
6.10
6.10
6.03
6.03
2.66
2.66
2.29
2.29
2.24
0.07
HN
O
N
N
N
5
1.26
1.00
0.99
3.10
3.09
3.05
11.0 10.5 10.0 9.5 9.0 8.5 8.0 7.5 7.0 6.5 6.0 5.5 5.0 4.5 4.0 3.5 3.0 2.5 2.0 1.5 1.0 0.5 0.0
f1 (ppm)

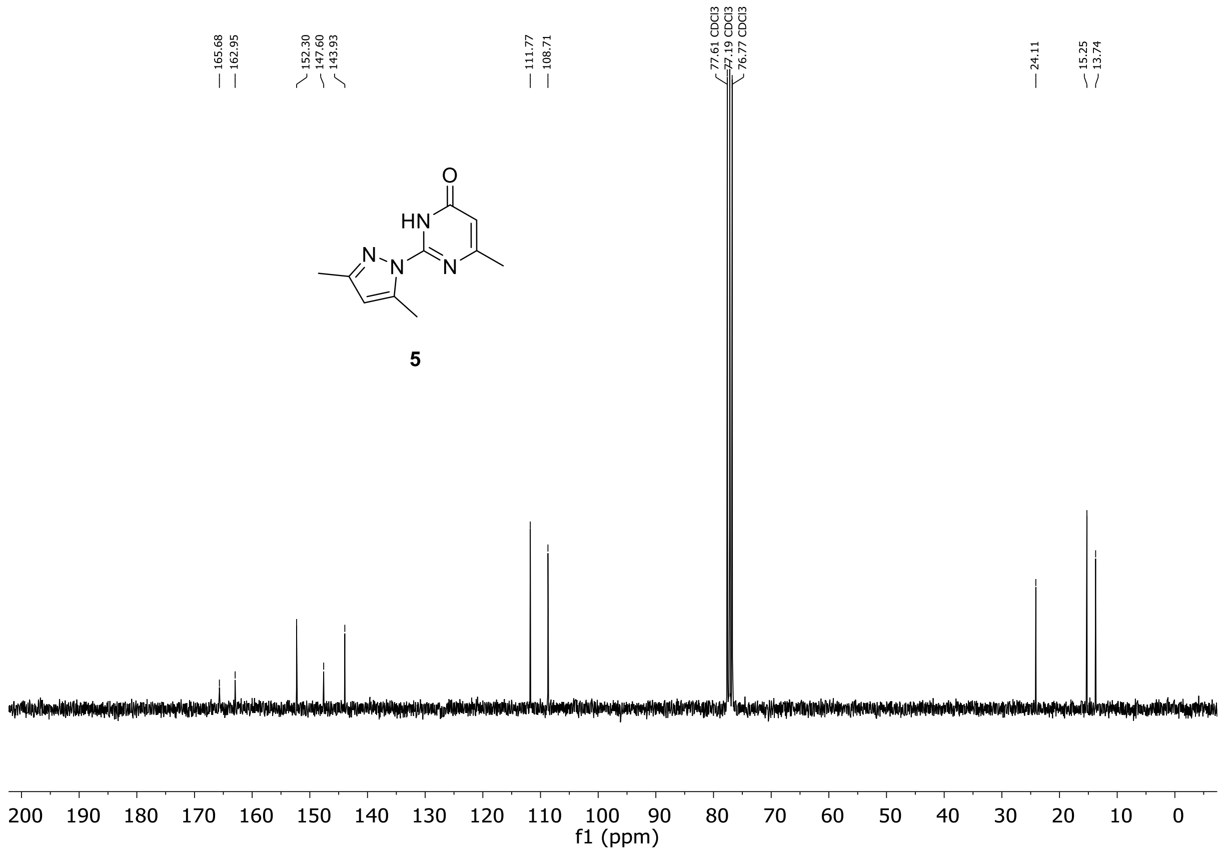
165.68
162.95
152.30
147.60
143.93
111.77
108.71
77.61 CDCl3
77.19 CDCl3
76.77 CDCl3
24.11
15.25
13.74
HN
O
N
N
N
5
200 190 180 170 160 150 140 130 120 110 100 90 80 70 60 50 40 30 20 10 0
f1 (ppm)

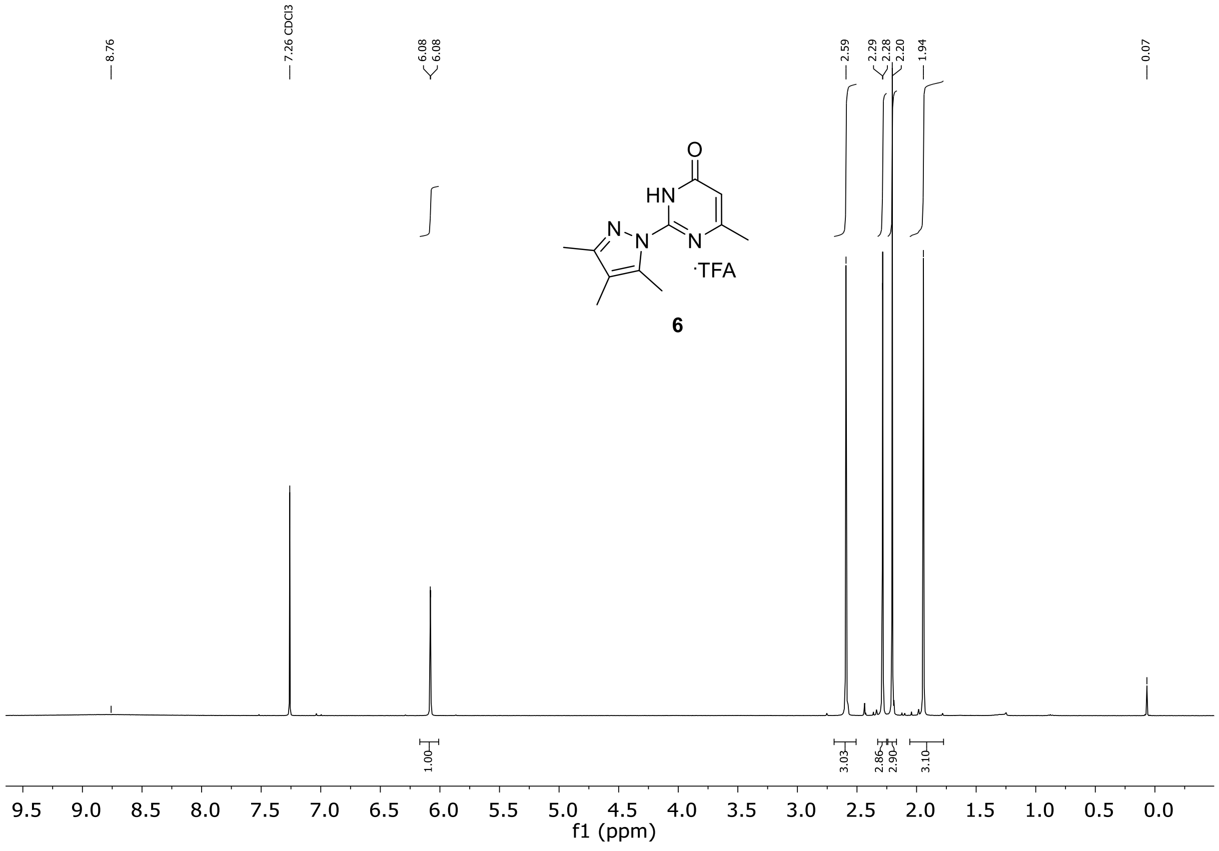
8.76
7.26 CDCl3
6.08
6.08
2.59
2.29
2.28
2.20
1.94
0.07
HN
O
N
N
N
·TFA
6
1.00
3.03
2.86
2.90
3.10
9.5 9.0 8.5 8.0 7.5 7.0 6.5 6.0 5.5 5.0 4.5 4.0 3.5 3.0 2.5 2.0 1.5 1.0 0.5 0.0
f1 (ppm)

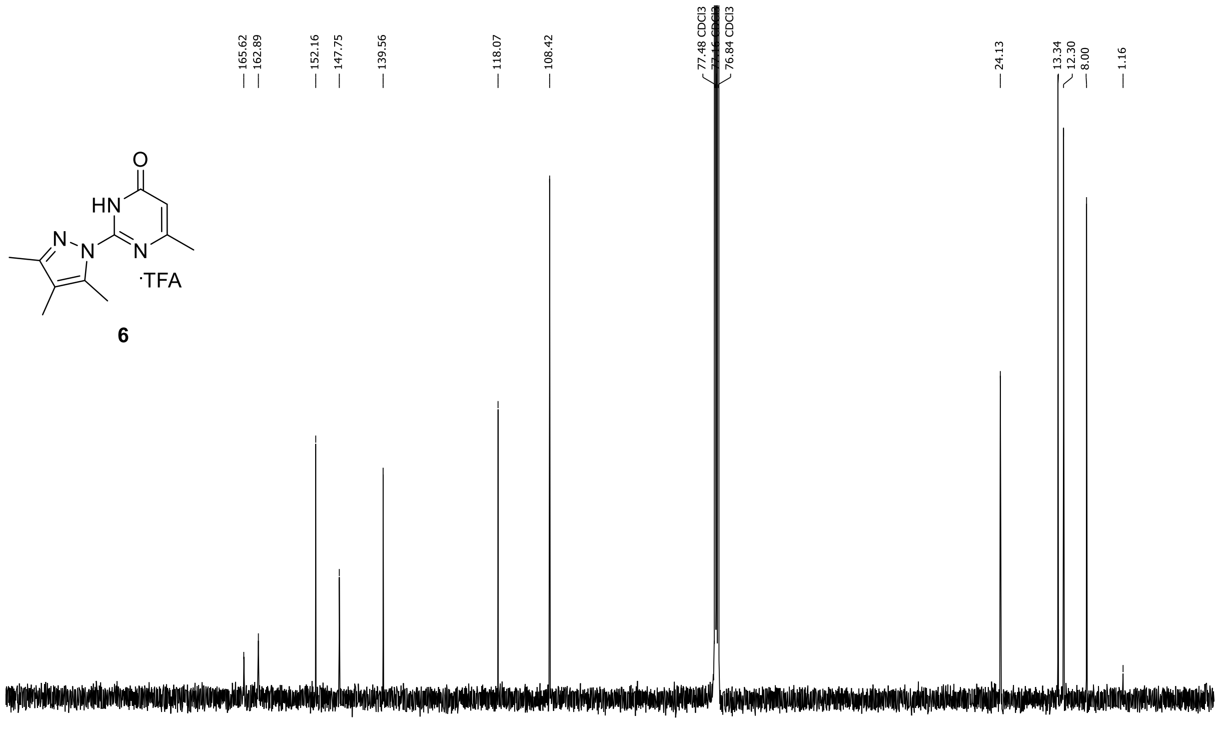
165.62
162.89
152.16
147.75
139.56
118.07
108.42
77.48 CDCl3
76.84 CDCl3
24.13
13.34
12.30
8.00
1.16
HN
O
N
N
N
·TFA
6
10 200 190 180 170 160 150 140 130 120 110 100 90 80 70 60 50 40 30 20 10 0 -10
f1 (ppm)

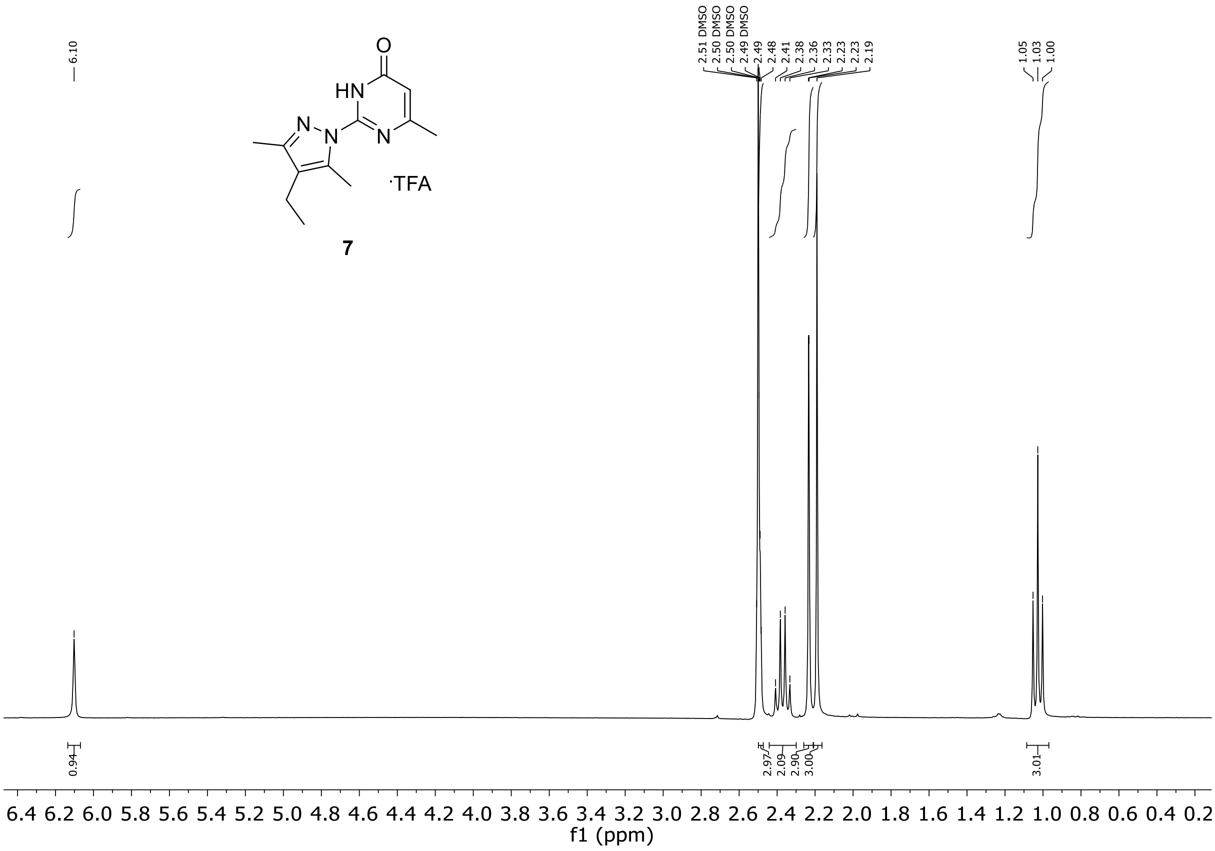

6.10
2.51 DMSO
2.50 DMSO
2.50 DMSO
2.49 DMSO
2.49
2.48
2.41
2.38
2.36
2.33
2.23
2.23
2.19
1.05
1.03
1.00
HN
O
N
N
N
·TFA
7
0.94
2.97
2.09
2.90
3.00
3.01
6.4 6.2 6.0 5.8 5.6 5.4 5.2 5.0 4.8 4.6 4.4 4.2 4.0 3.8 3.6 3.4 3.2 3.0 2.8 2.6 2.4 2.2 2.0 1.8 1.6 1.4 1.2 1.0 0.8 0.6 0.4 0.2
f1 (ppm)

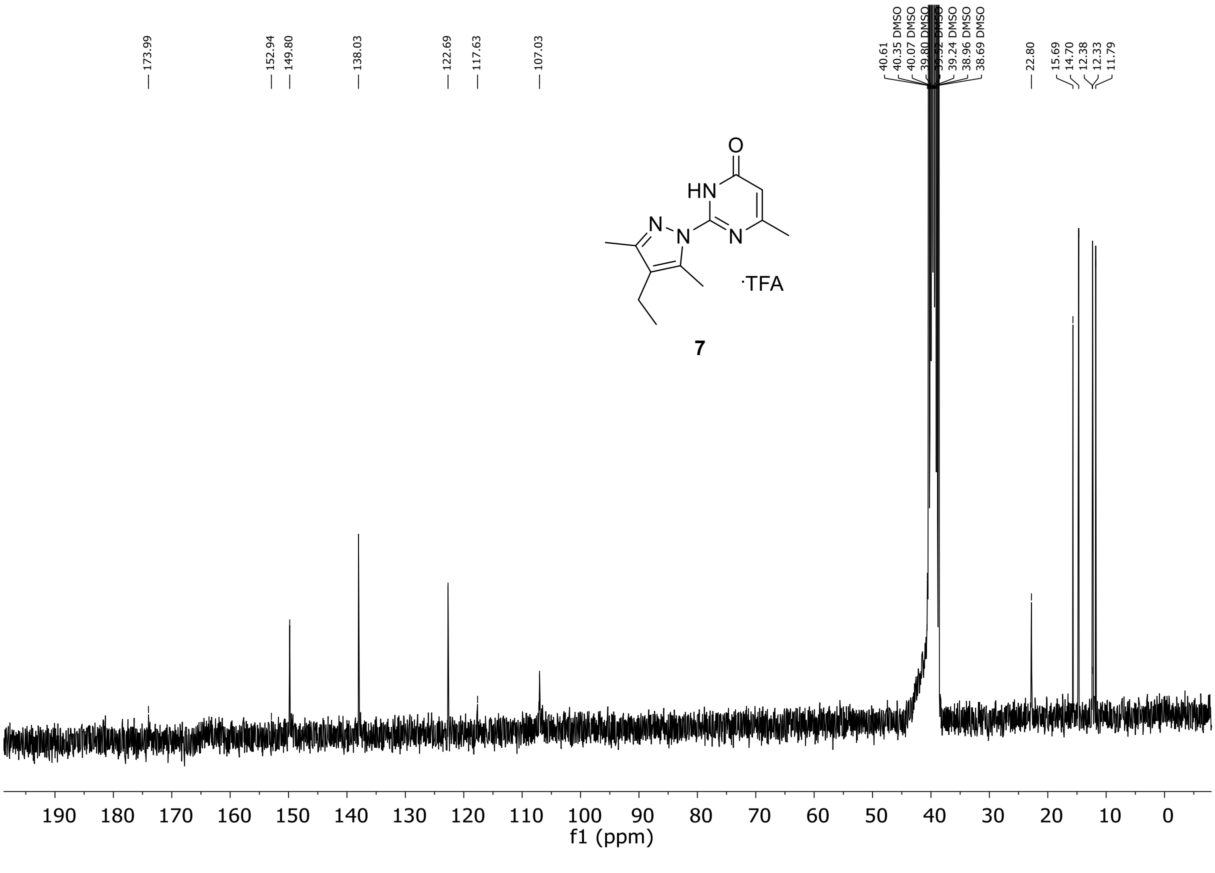

173.99
152.94
149.80
138.03
122.69
117.63
107.03
40.61
40.35 DMSO
40.07 DMSO
39.80 DMSO
39.52 DMSO
39.24 DMSO
38.96 DMSO
38.69 DMSO
22.80
15.69
14.70
12.38
12.33
11.79
HN
O
N
N
N
·TFA
7
190 180 170 160 150 140 130 120 110 100 90 80 70 60 50 40 30 20 10 0
f1 (ppm)

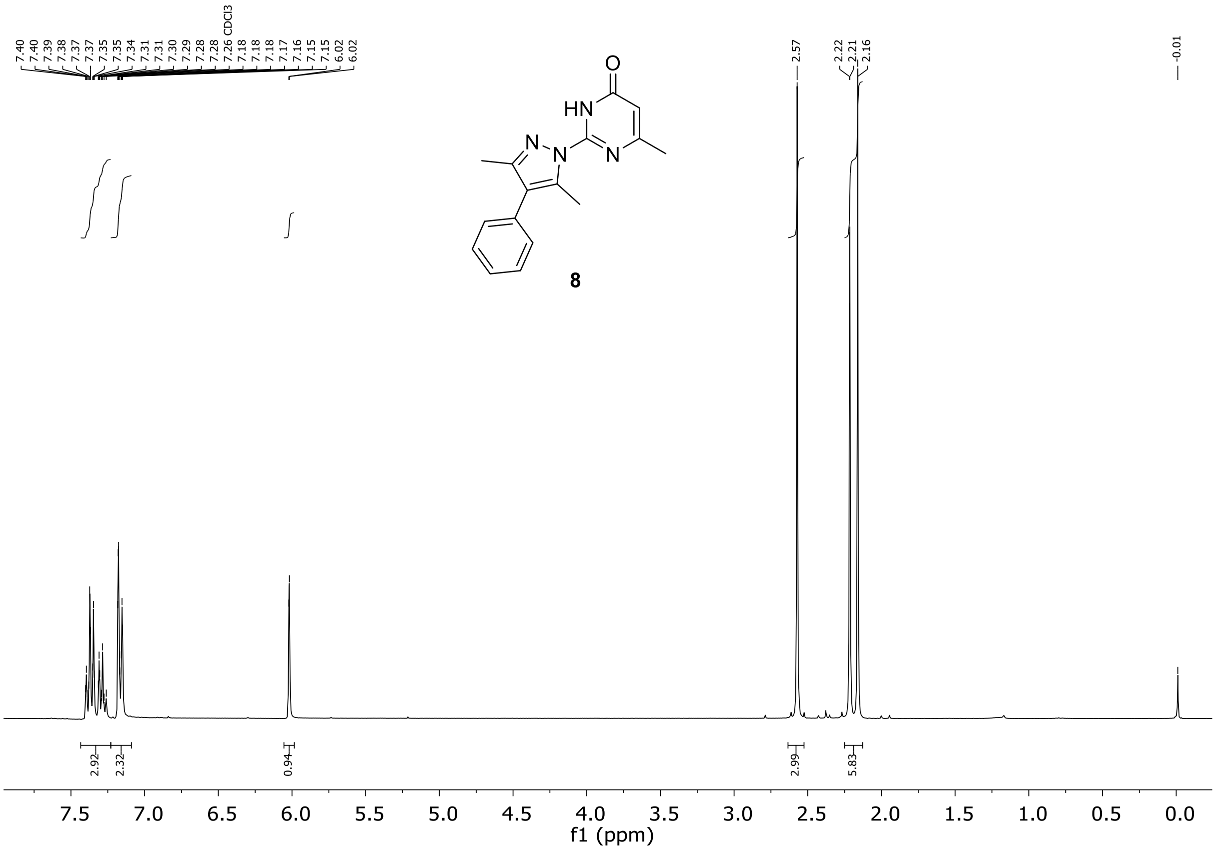
7.40
7.40
7.39
7.38
7.37
7.37
7.35
7.35
7.34
7.31
7.31
7.30
7.29
7.28
7.28
7.26 CDCl3
7.18
7.18
7.18
7.18
7.17
7.16
7.15
7.15
6.02
6.02
2.57
2.22
2.21
2.16
-0.01
O
HN
N
N
N
8
2.92
2.32
0.94
2.99
5.83
7.5 7.0 6.5 6.0 5.5 5.0 4.5 4.0 3.5 3.0 2.5 2.0 1.5 1.0 0.5 0.0
f1 (ppm)

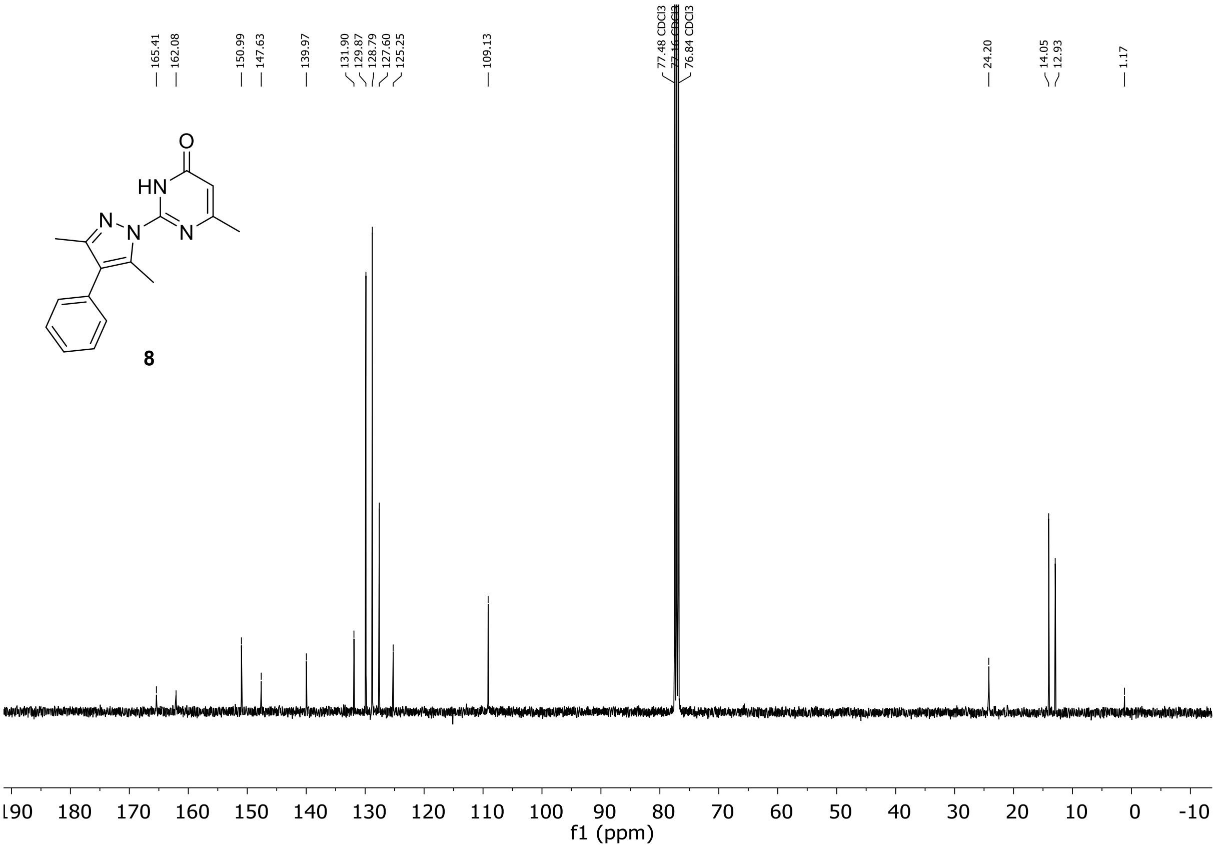
165.41
162.08
150.99
147.63
139.97
131.90
129.87
128.79
127.60
125.25
109.13
77.48 CDCl3
76.84 CDCl3
24.20
14.05
12.93
1.17
O
HN
N
N
N
8
180 170 160 150 140 130 120 110 100 90 80 70 60 50 40 30 20 10 0 -10
f1 (ppm)

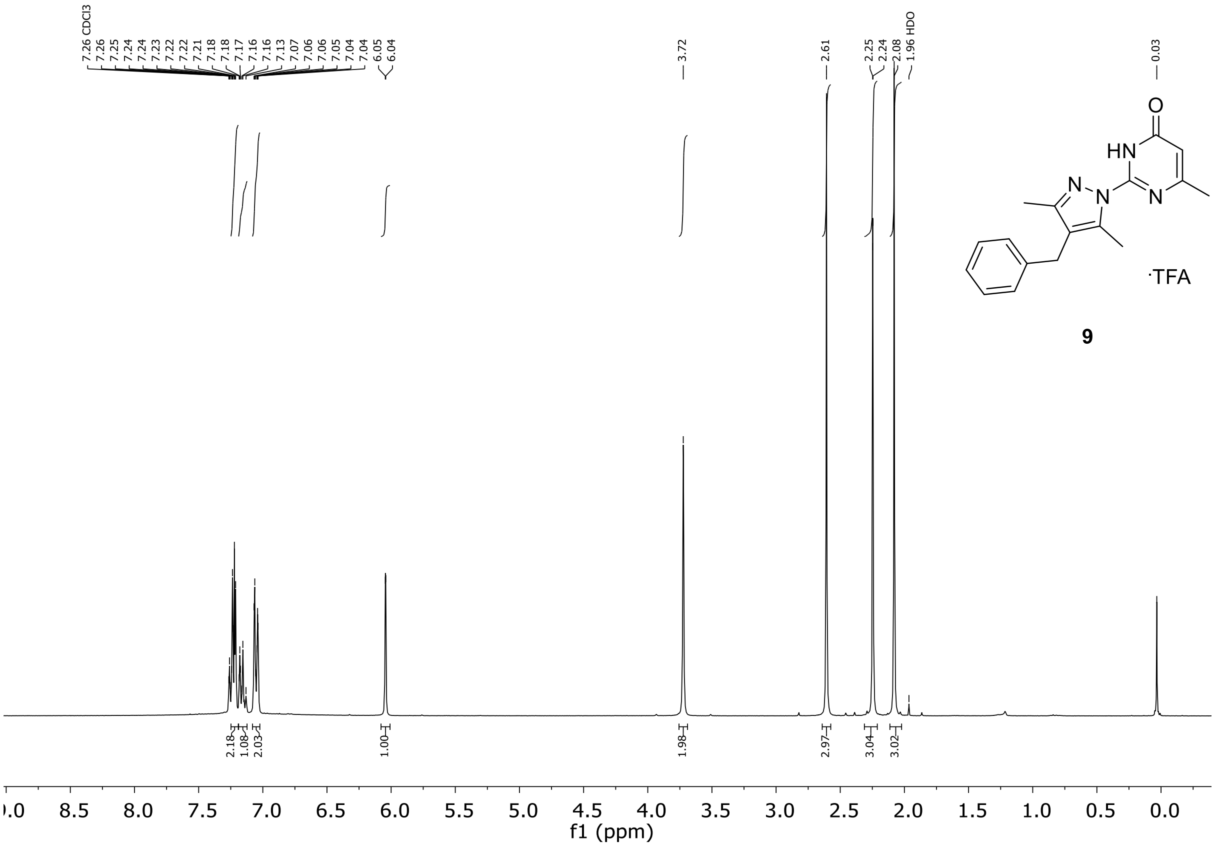
7.26 CDCl3
6.05
6.04
3.72
2.61
2.25
2.24
2.08
1.96 HDO
0.03
2.18
1.08
2.03
1.00
1.98
2.97
3.04
3.02
·TFA
9
f1 (ppm)

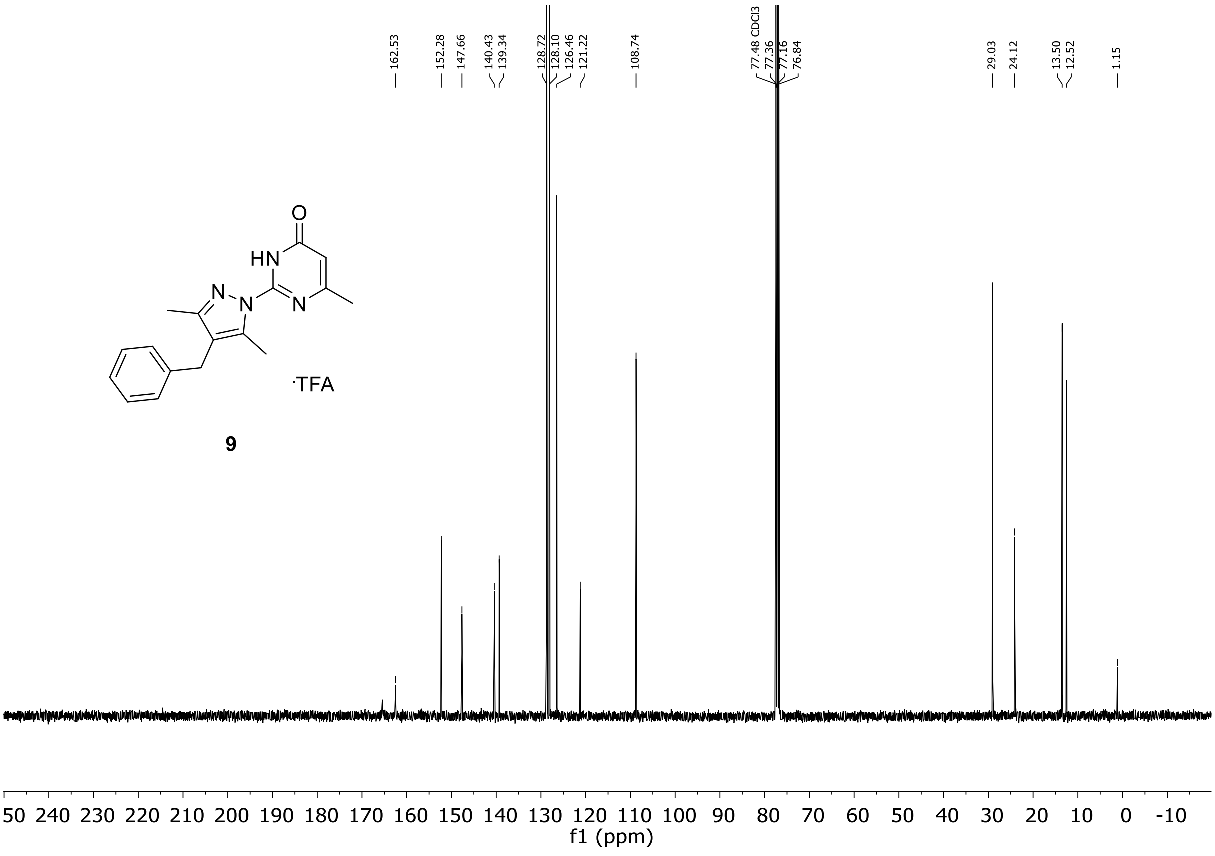
162.53
152.28
147.66
140.43
139.34
128.72
128.10
126.46
121.22
108.74
77.48 CDCl3
77.36
77.16
76.84
29.03
24.12
13.50
12.52
1.15
·TFA
9
f1 (ppm)

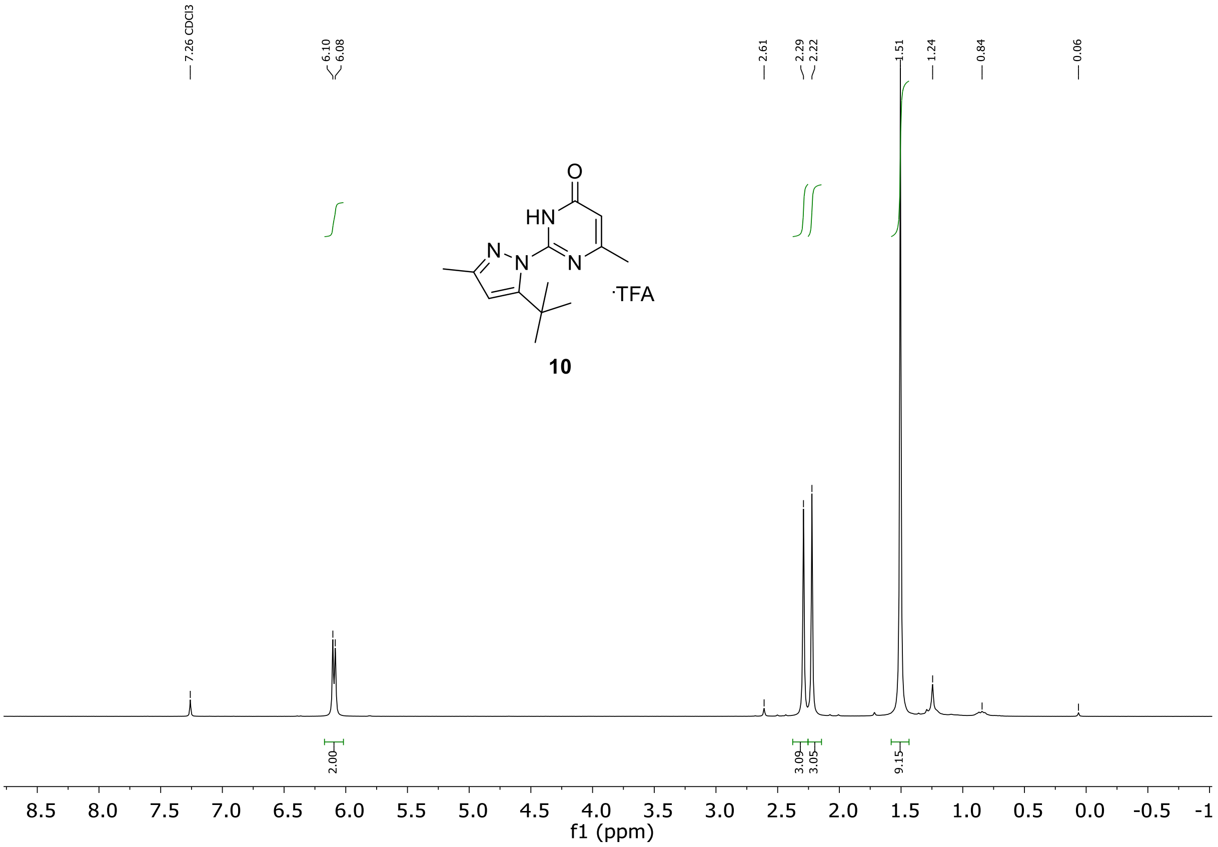
7.26 CDCl3
6.10
6.08
2.61
2.29
2.22
1.51
1.24
0.84
0.06
HN
O
N
N
N
·TFA
10
2.00
3.09
3.05
9.15
8.5 8.0 7.5 7.0 6.5 6.0 5.5 5.0 4.5 4.0 3.5 3.0 2.5 2.0 1.5 1.0 0.5 0.0 -0.5 -1
f1 (ppm)

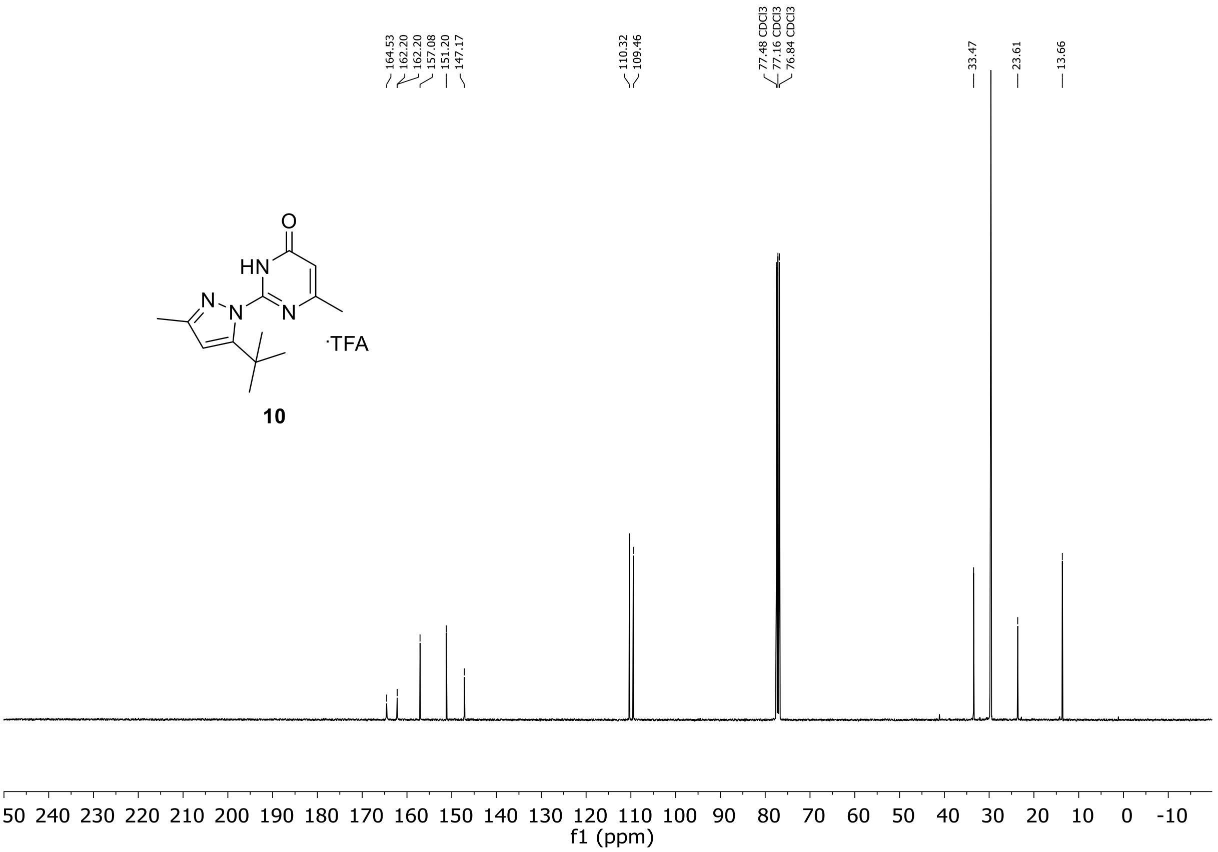
164.53
162.20
162.20
157.08
151.20
147.17
110.32
109.46
77.48 CDCl3
77.16 CDCl3
76.84 CDCl3
33.47
23.61
13.66
HN
O
N
N
N
·TFA
10
50 240 230 220 210 200 190 180 170 160 150 140 130 120 110 100 90 80 70 60 50 40 30 20 10 0 -10
f1 (ppm)

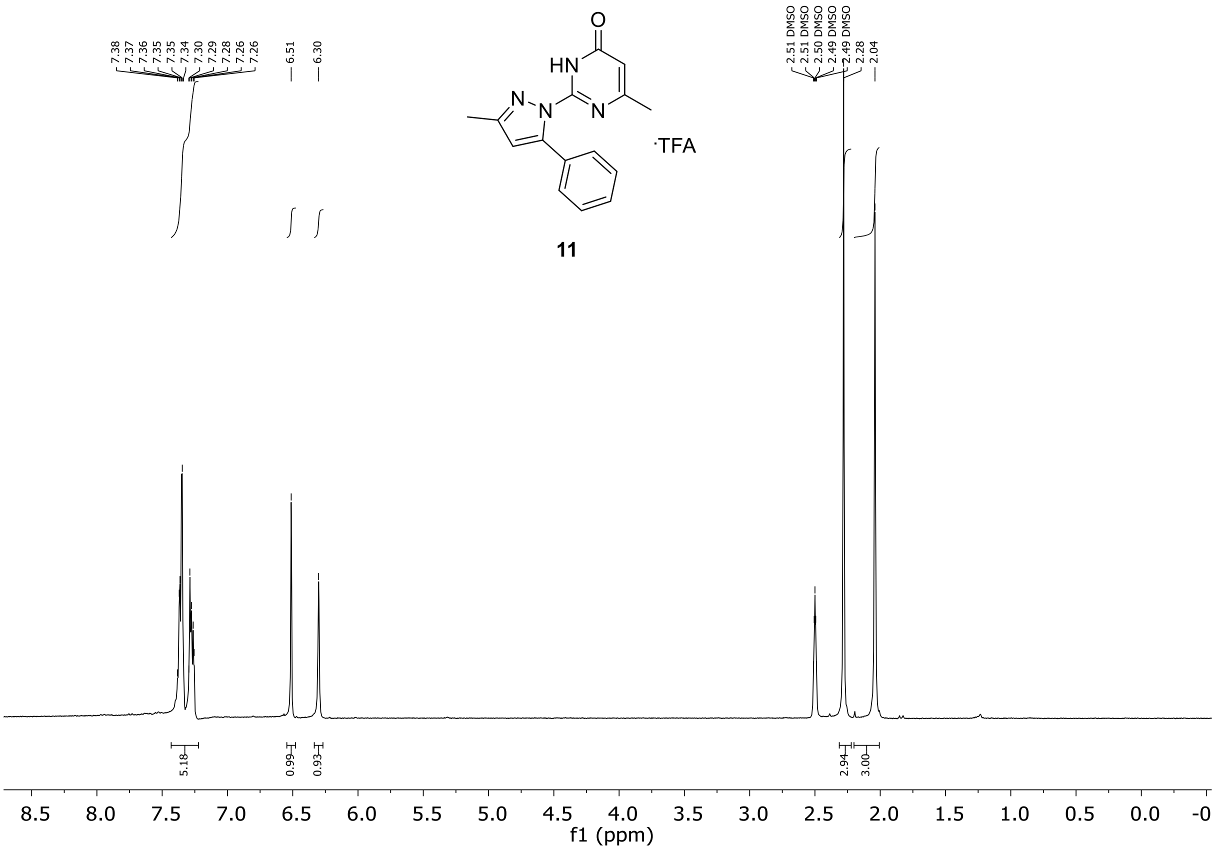
7.38
7.37
7.36
7.35
7.35
7.34
7.30
7.29
7.28
7.26
7.26
6.51
6.30
2.51 DMSO
2.51 DMSO
2.50 DMSO
2.49 DMSO
2.49 DMSO
2.28
2.04
HN
O
N
N
N
N
·TFA
11
5.18
0.99
0.93
2.94
3.00
8.5 8.0 7.5 7.0 6.5 6.0 5.5 5.0 4.5 4.0 3.5 3.0 2.5 2.0 1.5 1.0 0.5 0.0 -0
f1 (ppm)

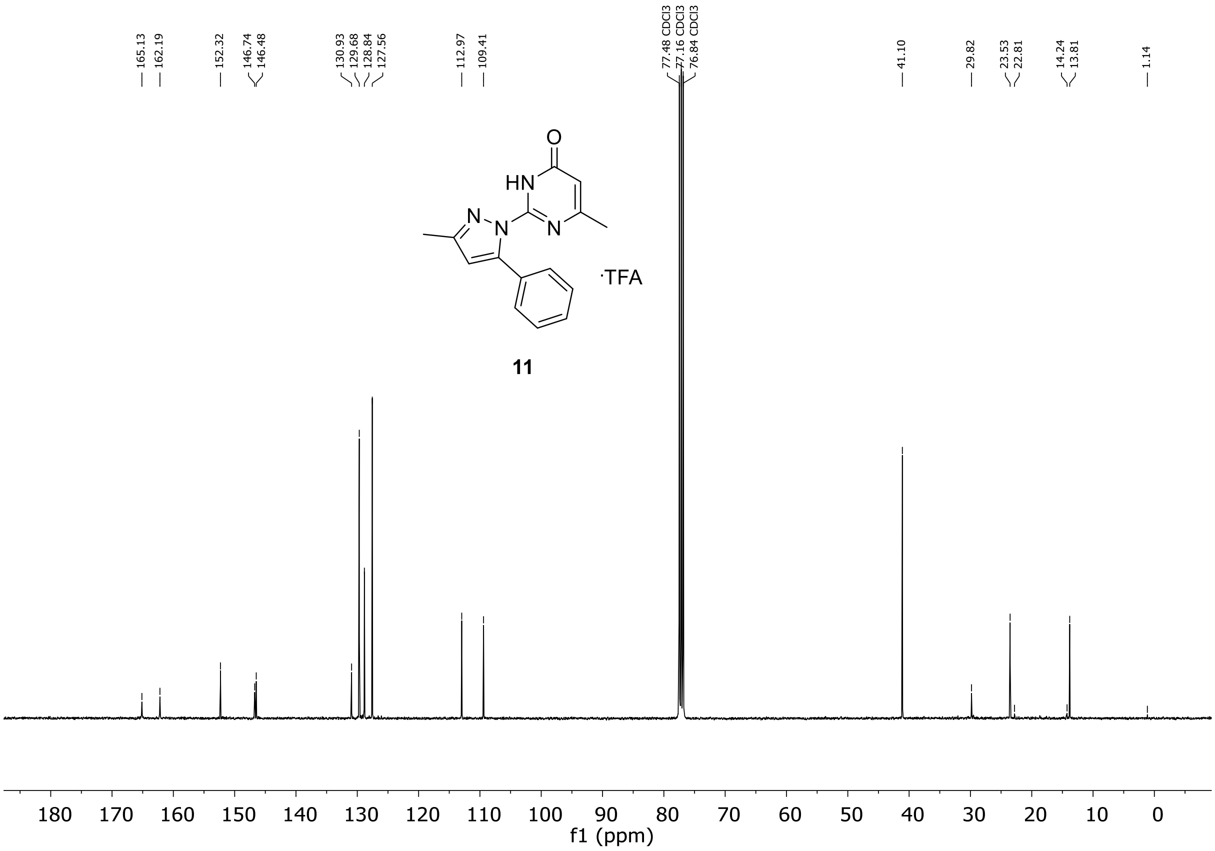
165.13
162.19
152.32
146.74
146.48
130.93
129.68
128.84
127.56
112.97
109.41
77.48 CDCl3
77.16 CDCl3
76.84 CDCl3
41.10
29.82
23.53
22.81
14.24
13.81
1.14
HN
O
N
N
N
N
·TFA
11
180 170 160 150 140 130 120 110 100 90 80 70 60 50 40 30 20 10 0
f1 (ppm)

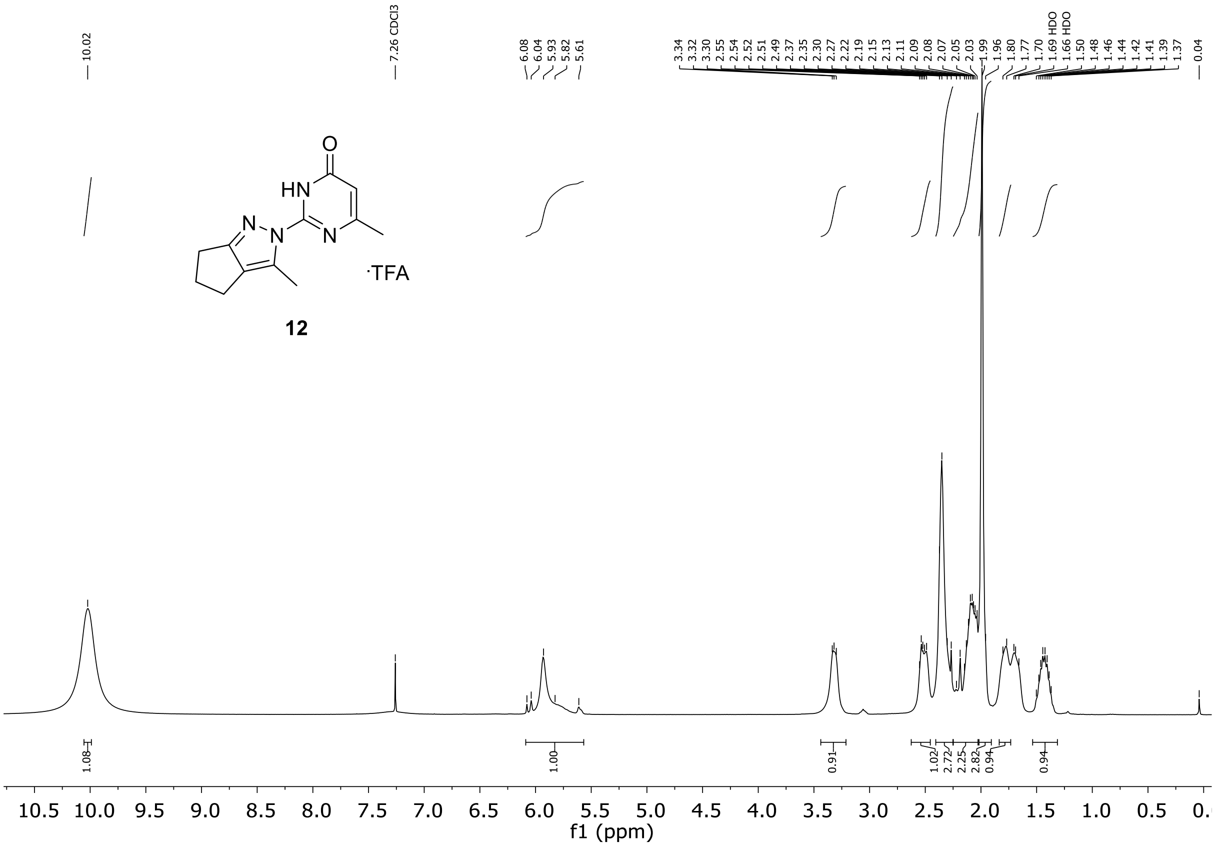
10.02
7.26 CDCl3
6.08
6.04
5.93
5.82
5.61
3.34
3.32
3.30
2.55
2.54
2.52
2.51
2.49
2.37
2.35
2.30
2.27
2.22
2.19
2.15
2.13
2.11
2.09
2.08
2.07
2.05
2.03
1.99
1.96
1.80
1.77
1.70
1.69 HDO
1.66 HDO
1.50
1.48
1.46
1.44
1.42
1.41
1.39
1.37
0.04
O
HN
N
N
N
·TFA
12
1.08
1.00
0.91
1.02
2.72
2.25
2.82
0.94
0.94
10.5 10.0 9.5 9.0 8.5 8.0 7.5 7.0 6.5 6.0 5.5 5.0 4.5 4.0 3.5 3.0 2.5 2.0 1.5 1.0 0.5 0.
f1 (ppm)

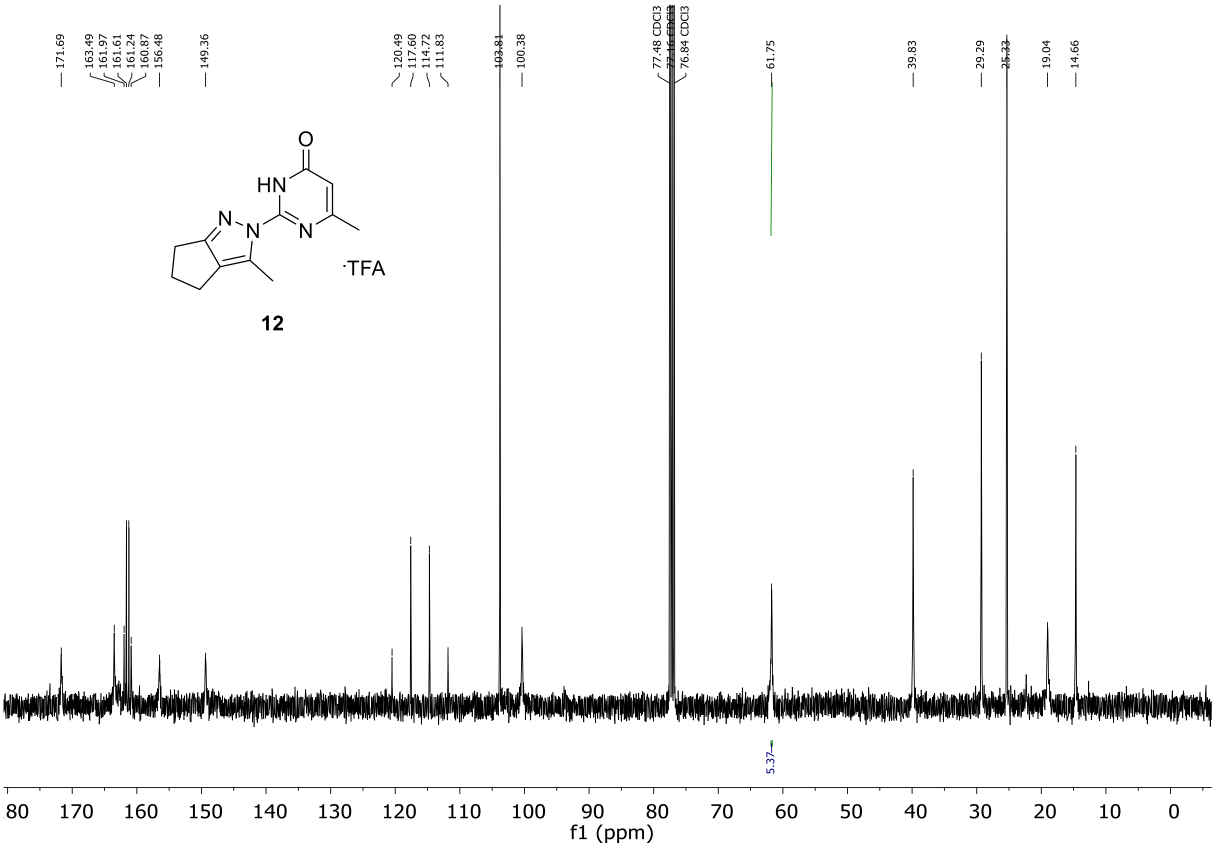
171.69
163.49
161.97
161.61
161.24
160.87
156.48
149.36
120.49
117.60
114.72
111.83
103.81
100.38
77.48 CDCl3
77.16 CDCl3
76.84 CDCl3
61.75
39.83
29.29
25.33
19.04
14.66
O
HN
N
N
N
·TFA
12
5.37
80 170 160 150 140 130 120 110 100 90 80 70 60 50 40 30 20 10 0
f1 (ppm)

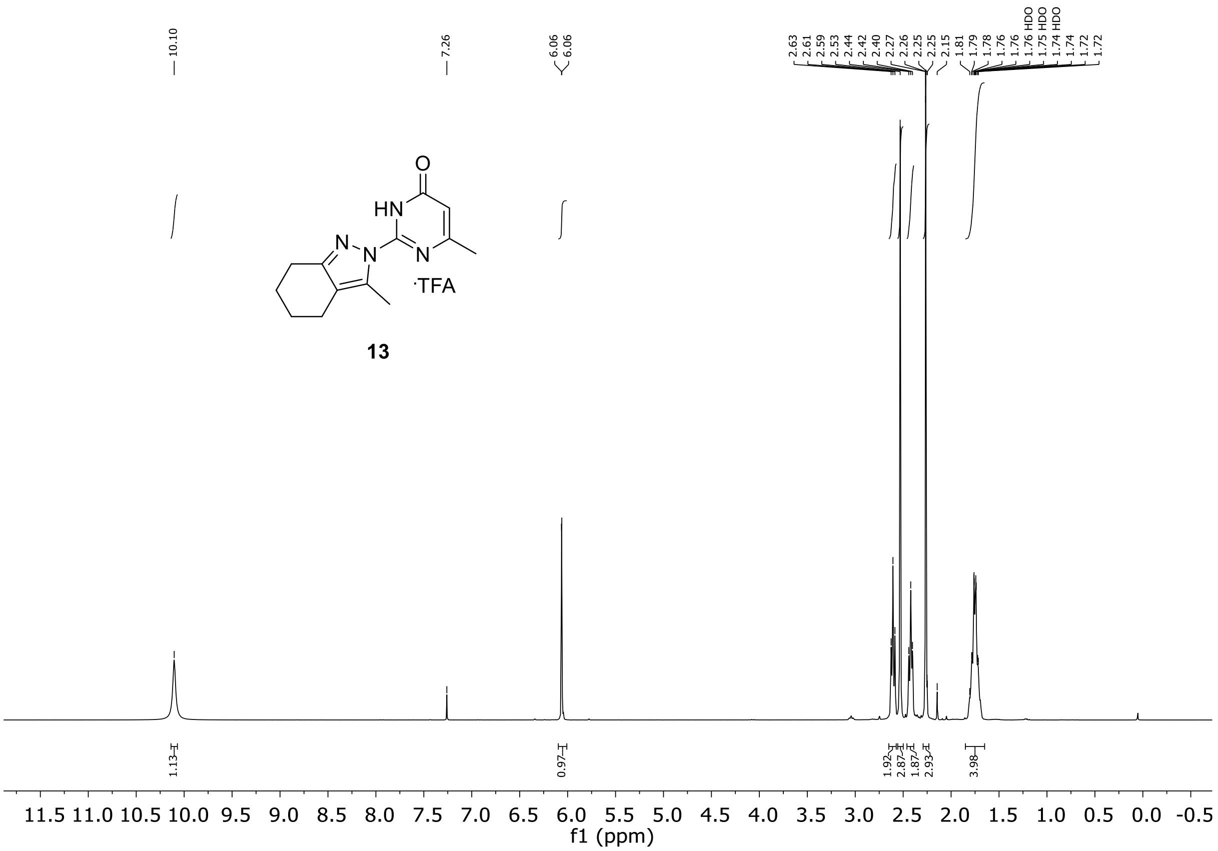
10.10
7.26
6.06
6.06
2.63 2.61 2.59 2.53 2.44 2.42 2.40 2.27 2.26 2.25 2.25 2.15 1.81 1.79 1.78 1.76 1.76 1.76 HDO 1.75 HDO 1.74 HDO 1.74 1.72 1.72
O
HN
N
N
N
·TFA
13
1.13
0.97
1.92
2.87
1.87
2.93
3.98
11.5 11.0 10.5 10.0 9.5 9.0 8.5 8.0 7.5 7.0 6.5 6.0 5.5 5.0 4.5 4.0 3.5 3.0 2.5 2.0 1.5 1.0 0.5 0.0 -0.5
f1 (ppm)

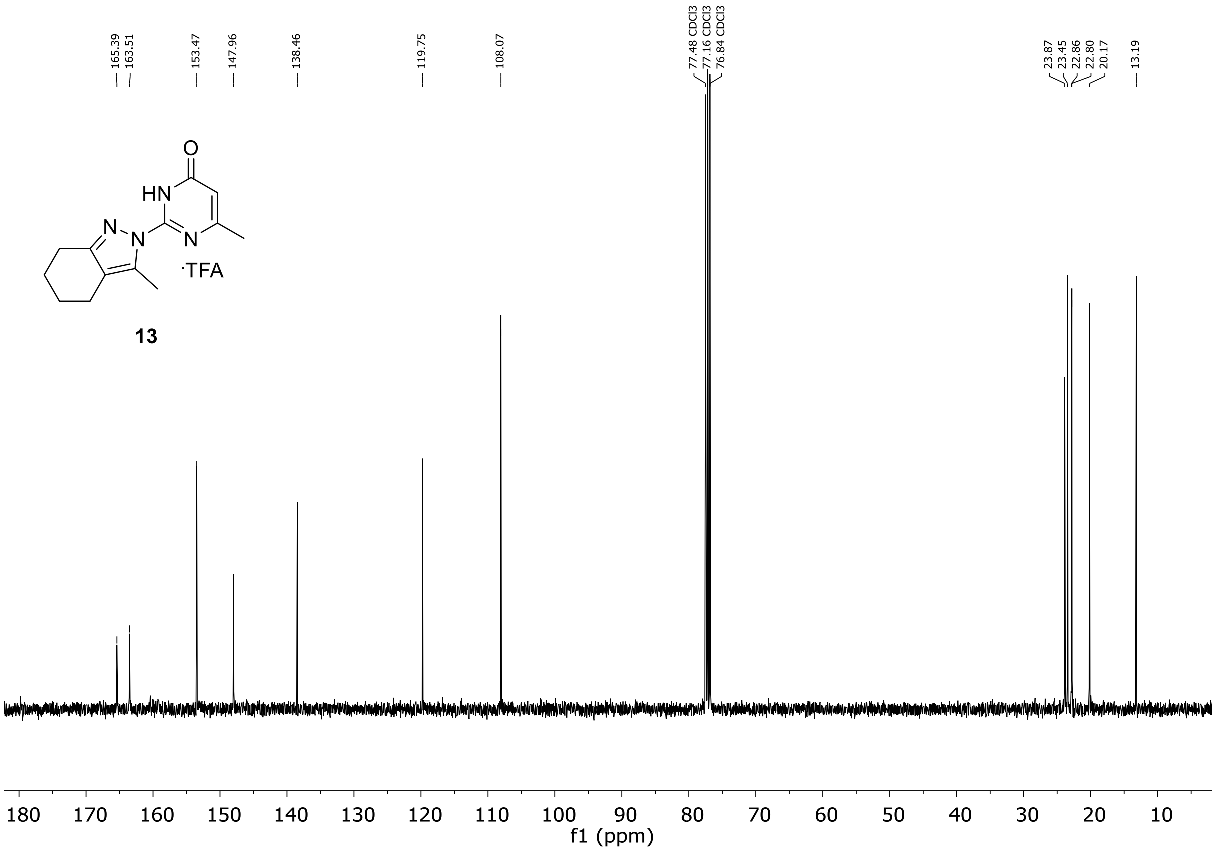
165.39
163.51
153.47
147.96
138.46
119.75
108.07
77.48 CDCl3
77.16 CDCl3
76.84 CDCl3
23.87
23.45
22.86
22.80
20.17
13.19
O
HN
N
N
N
·TFA
13
180 170 160 150 140 130 120 110 100 90 80 70 60 50 40 30 20 10
f1 (ppm)

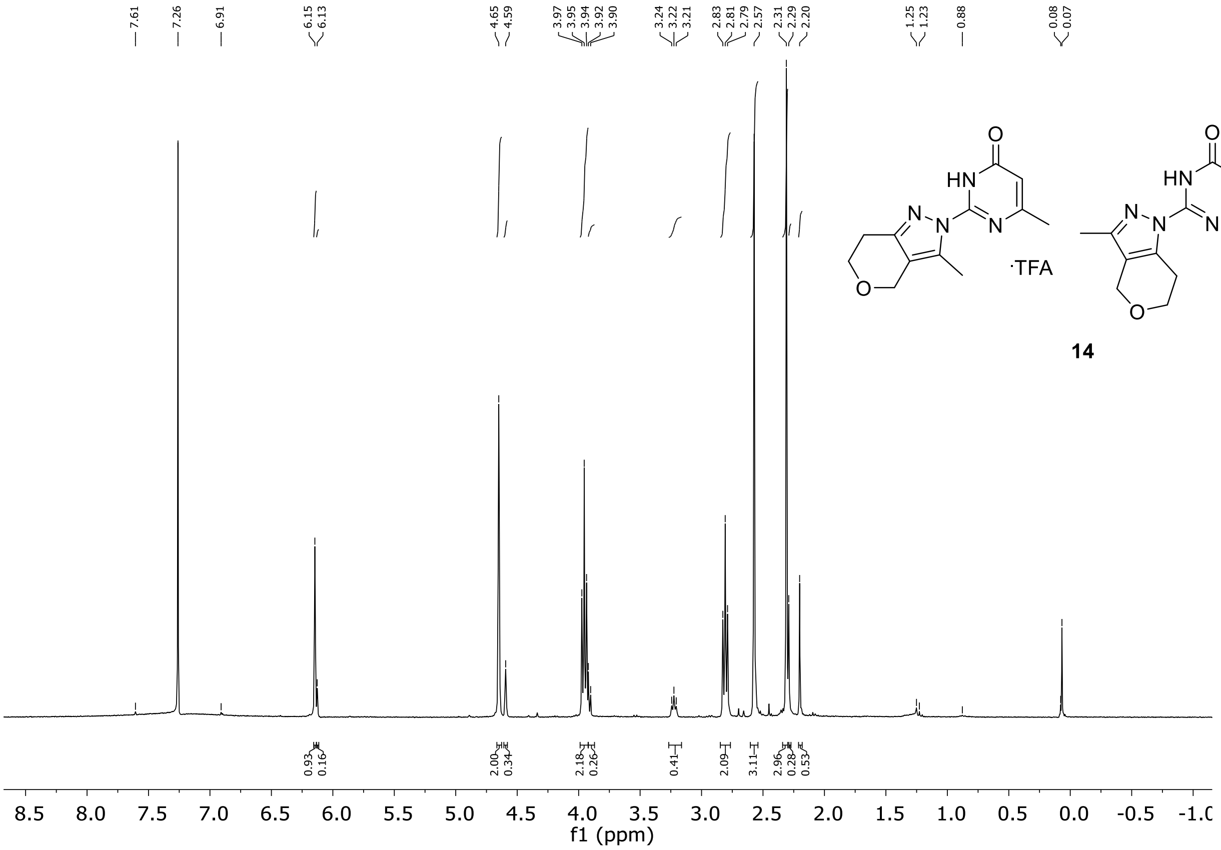
7.61
7.26
6.91
6.15
6.13
4.65
4.59
3.97
3.95
3.94
3.92
3.90
3.24
3.22
3.21
2.83
2.81
2.79
2.57
2.31
2.29
2.20
1.25
1.23
0.88
0.08
0.07
HN
N
N
N
O
·TFA
14
0.93
0.16
2.00
0.34
2.18
0.26
0.41
2.09
3.11
2.96
0.28
0.53
f1 (ppm)

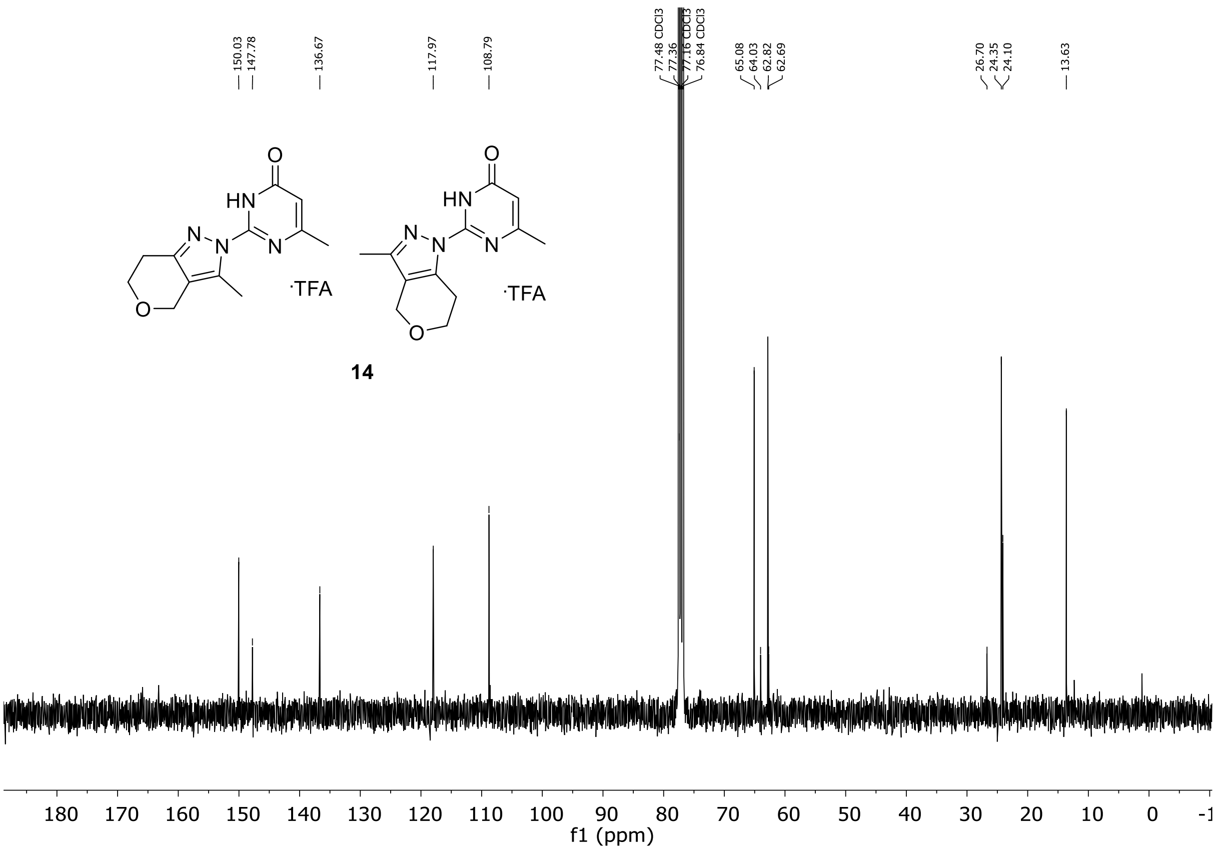
150.03
147.78
136.67
117.97
108.79
77.48 CDCl3
77.36
77.16 CDCl3
76.84 CDCl3
65.08
64.03
62.82
62.69
26.70
24.35
24.10
13.63
HN
N
N
N
O
·TFA
14
f1 (ppm)

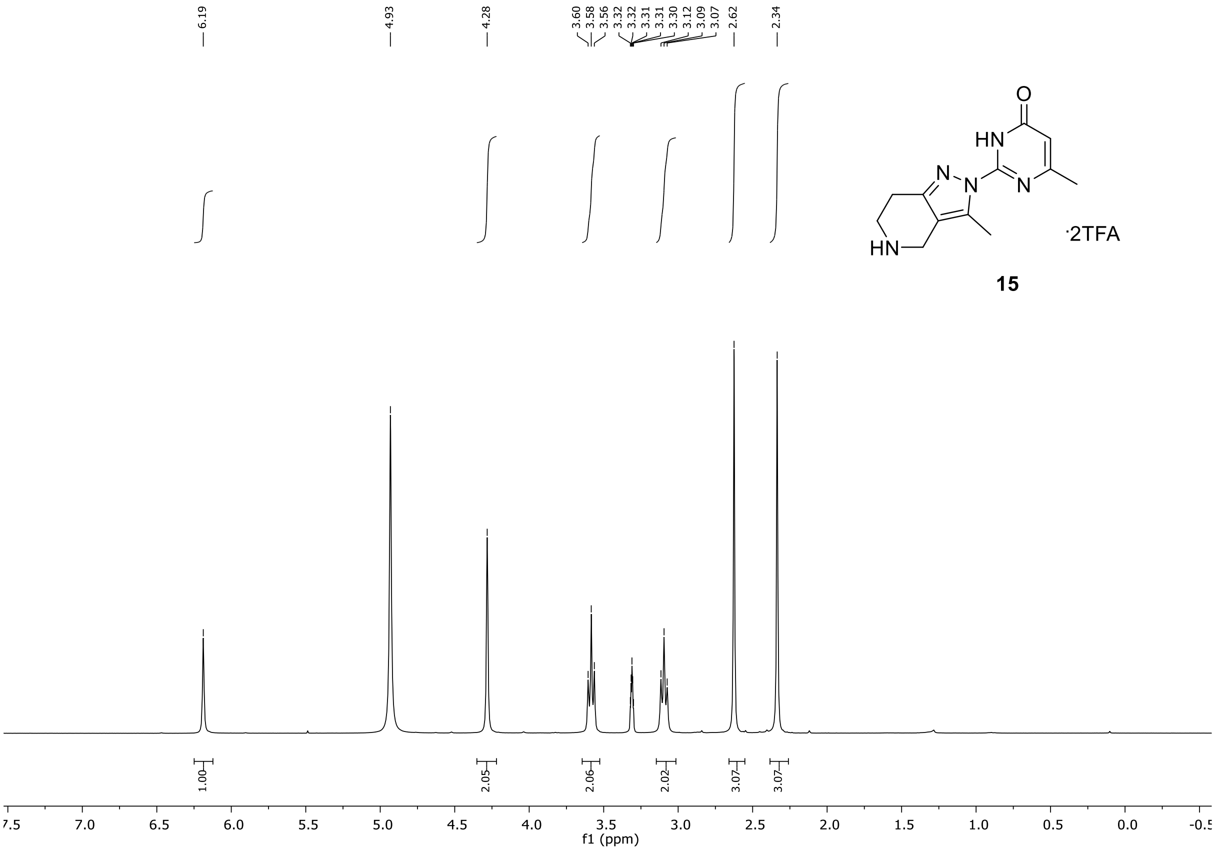
6.19
4.93
4.28
3.60 3.58 3.56 3.32 3.32 3.31 3.31 3.30 3.12 3.09 3.07
2.62
2.34
HN
N
N
N
O
HN
·2TFA
15
1.00
2.05
2.06
2.02
3.07
3.07
f1 (ppm)

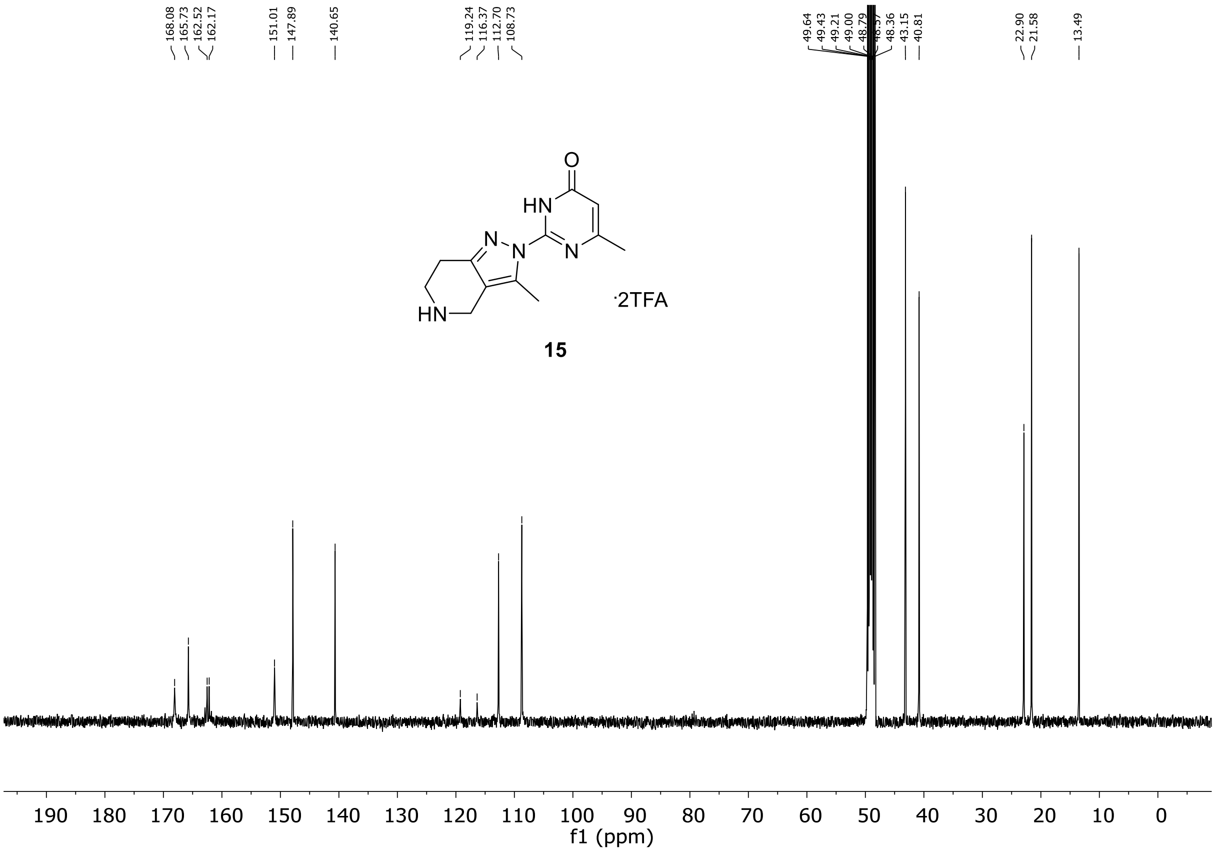
168.08 165.73 162.52 162.17
151.01 147.89
140.65
119.24 116.37 112.70 108.73
49.64 49.43 49.21 49.00 48.79 48.36 43.15 40.81
22.90 21.58
13.49
O
HN
N
N
N
HN
·2TFA
15
f1 (ppm)

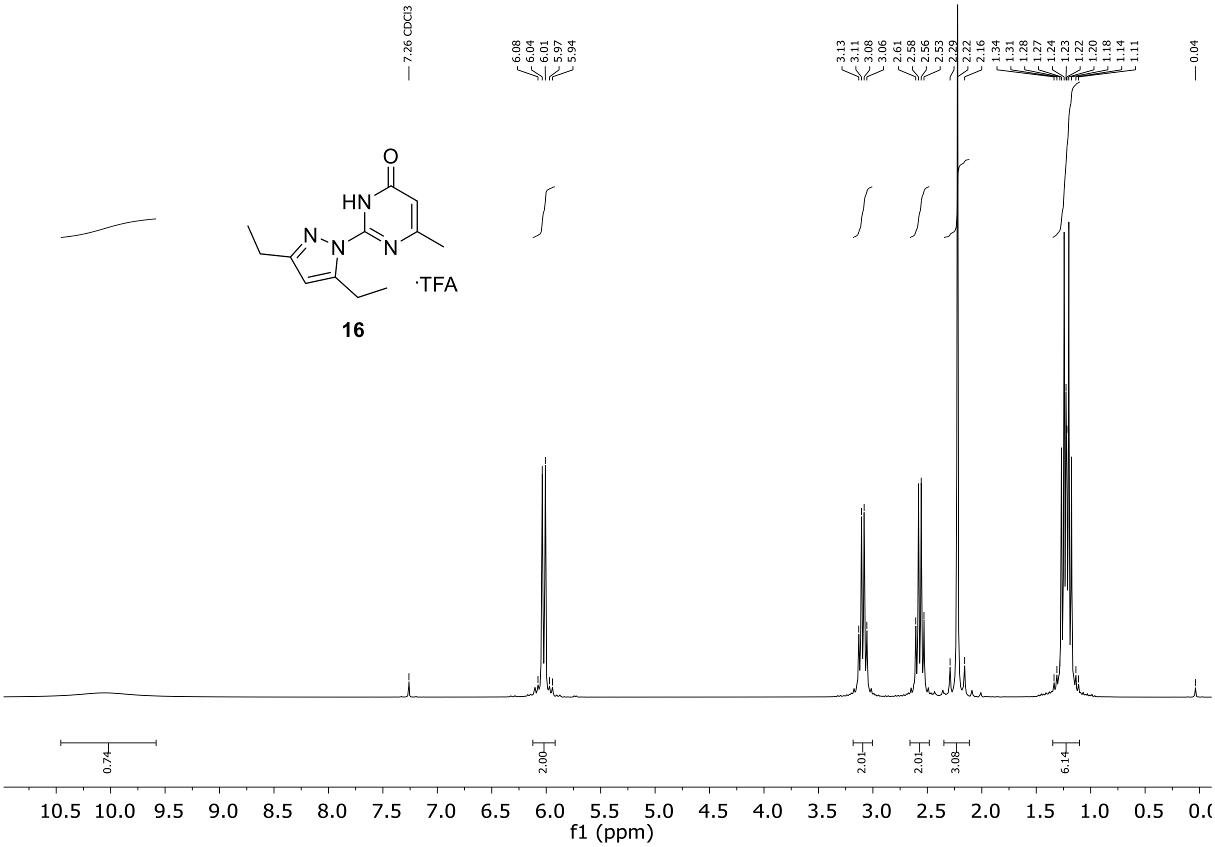
7.26 CDCl3
6.08
6.04
6.01
5.97
5.94
3.13
3.11
3.08
3.06
2.61
2.58
2.56
2.53
2.29
2.22
2.16
1.34
1.31
1.28
1.27
1.24
1.23
1.22
1.20
1.18
1.14
1.11
0.04
·TFA
16
0.74
2.00
2.01
2.01
3.08
6.14
f1 (ppm)

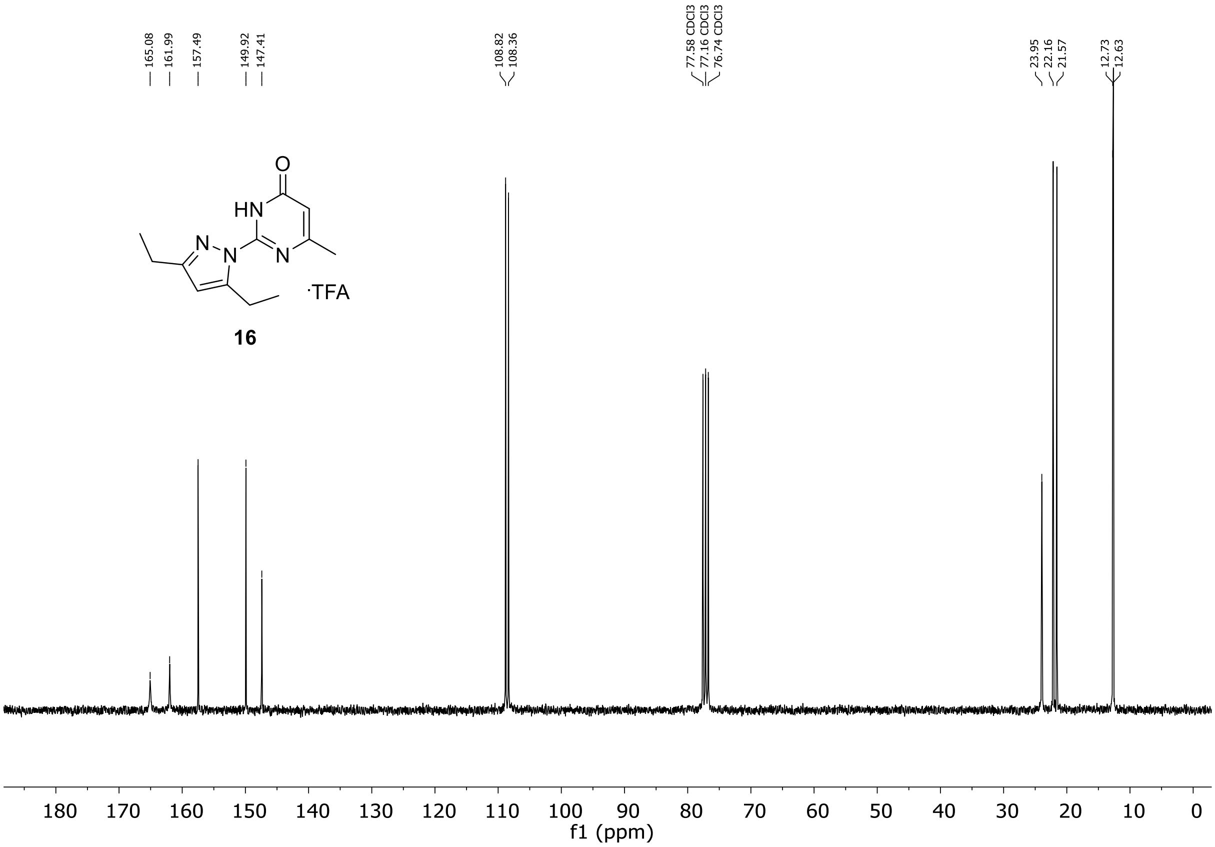
165.08
161.99
157.49
149.92
147.41
108.82
108.36
77.58 CDCl3
77.16 CDCl3
76.74 CDCl3
23.95
22.16
21.57
12.73
12.63
·TFA
16
f1 (ppm)

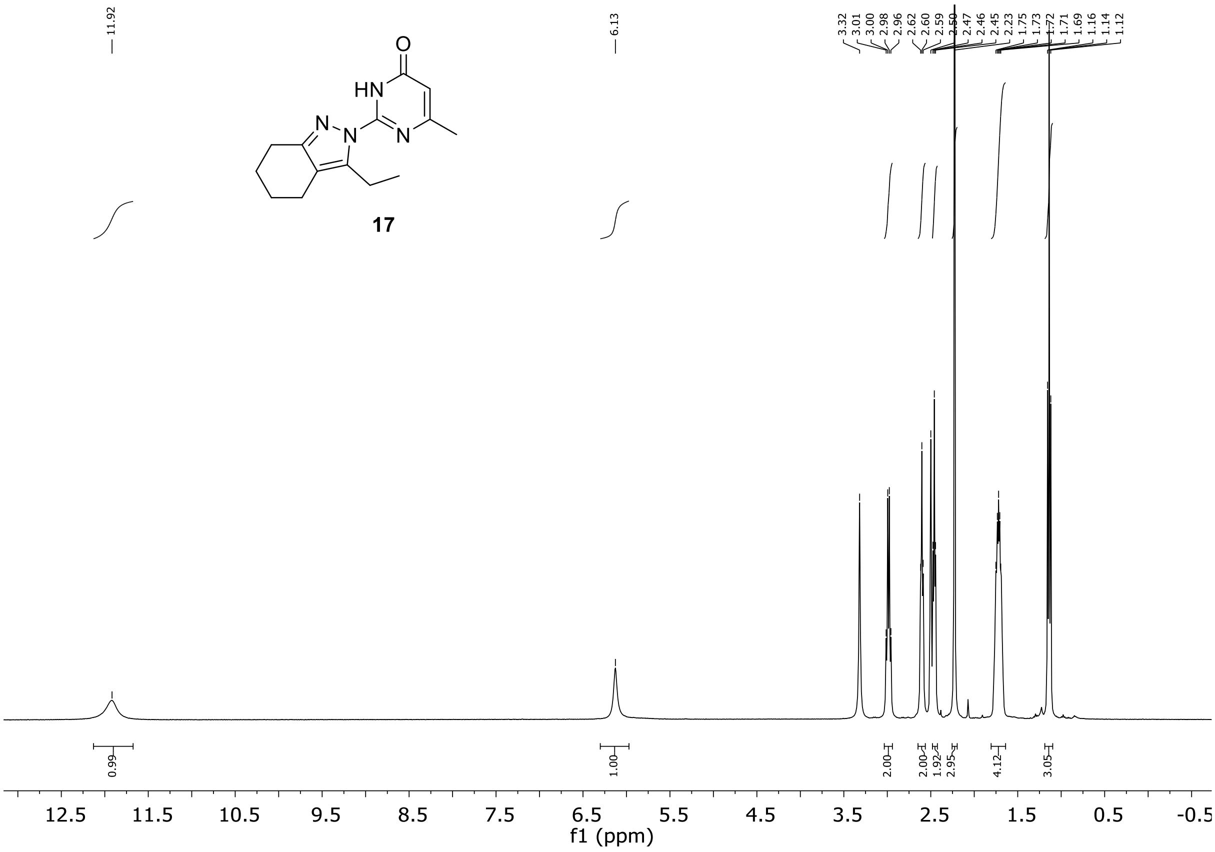
11.92
6.13
3.32
3.01
3.00
2.98
2.96
2.62
2.60
2.59
2.47
2.46
2.45
2.23
1.75
1.73
1.72
1.71
1.69
1.16
1.14
1.12
O
HN
N
N
N
17
0.99
1.00
2.00
2.00
1.92
2.95
4.12
3.05
12.5 11.5 10.5 9.5 8.5 7.5 6.5 5.5 4.5 3.5 2.5 1.5 0.5 -0.5
f1 (ppm)

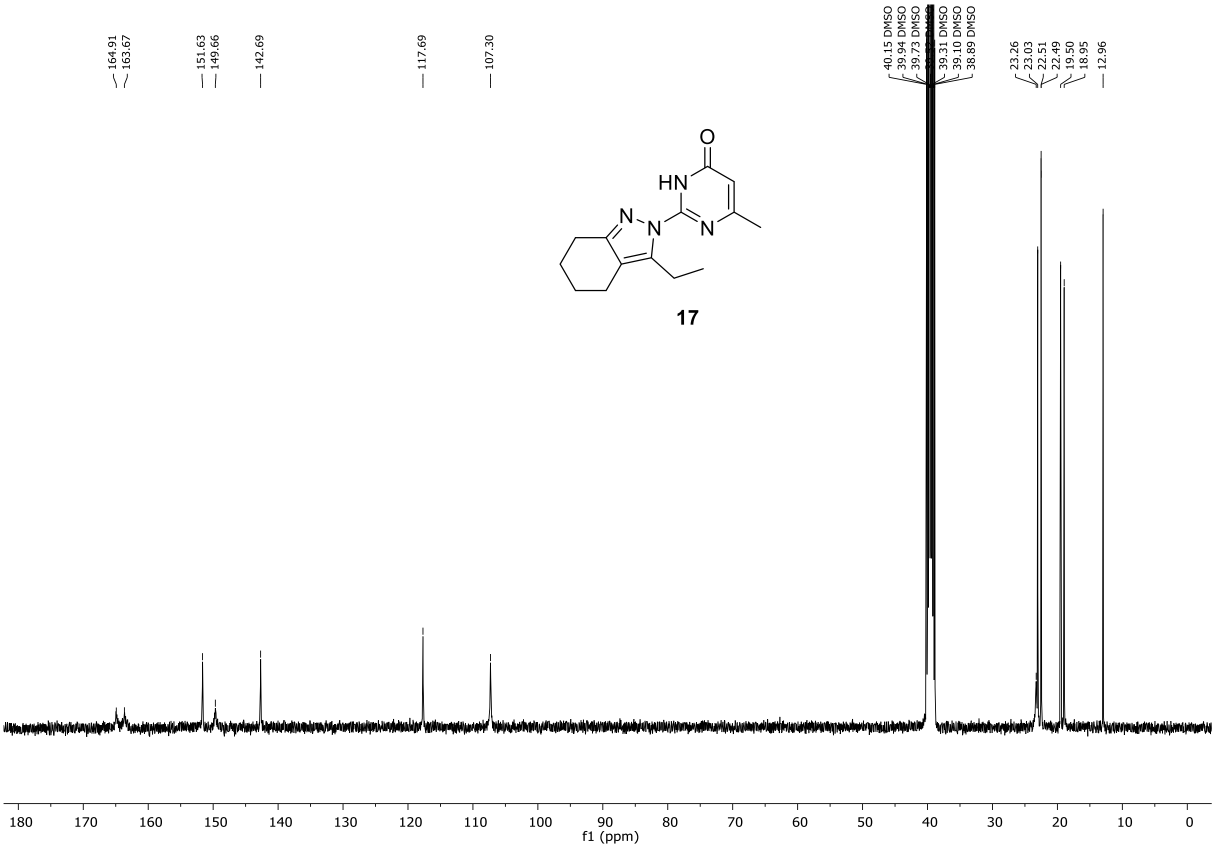
164.91
163.67
151.63
149.66
142.69
117.69
107.30
40.15 DMSO
39.94 DMSO
39.73 DMSO
39.31 DMSO
39.10 DMSO
38.89 DMSO
23.26
23.03
22.51
22.49
19.50
18.95
12.96
O
HN
N
N
N
17
180 170 160 150 140 130 120 110 100 90 80 70 60 50 40 30 20 10 0
f1 (ppm)

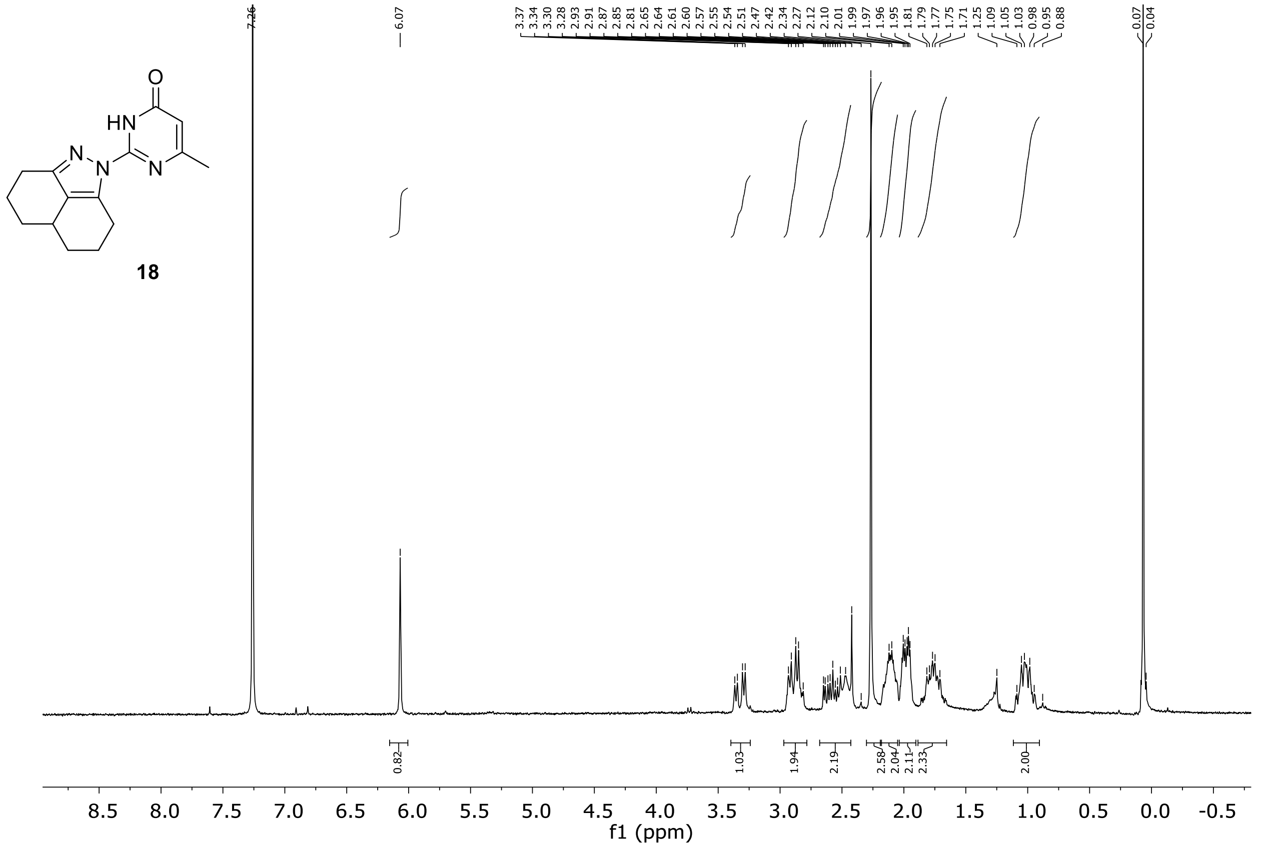
7.26
6.07
3.37
3.34
3.30
3.28
2.93
2.91
2.87
2.85
2.81
2.65
2.64
2.61
2.60
2.57
2.55
2.54
2.51
2.47
2.42
2.34
2.27
2.12
2.10
2.01
1.99
1.97
1.96
1.95
1.81
1.79
1.77
1.75
1.71
1.25
1.09
1.05
1.03
0.98
0.95
0.88
0.07
0.04
O
HN
N
N
N
18
0.82
1.03
1.94
2.19
2.58
2.04
2.11
2.33
2.00
8.5
8.0
7.5
7.0
6.5
6.0
5.5
5.0
4.5
4.0
3.5
3.0
2.5
2.0
1.5
1.0
0.5
0.0
-0.5
f1 (ppm)

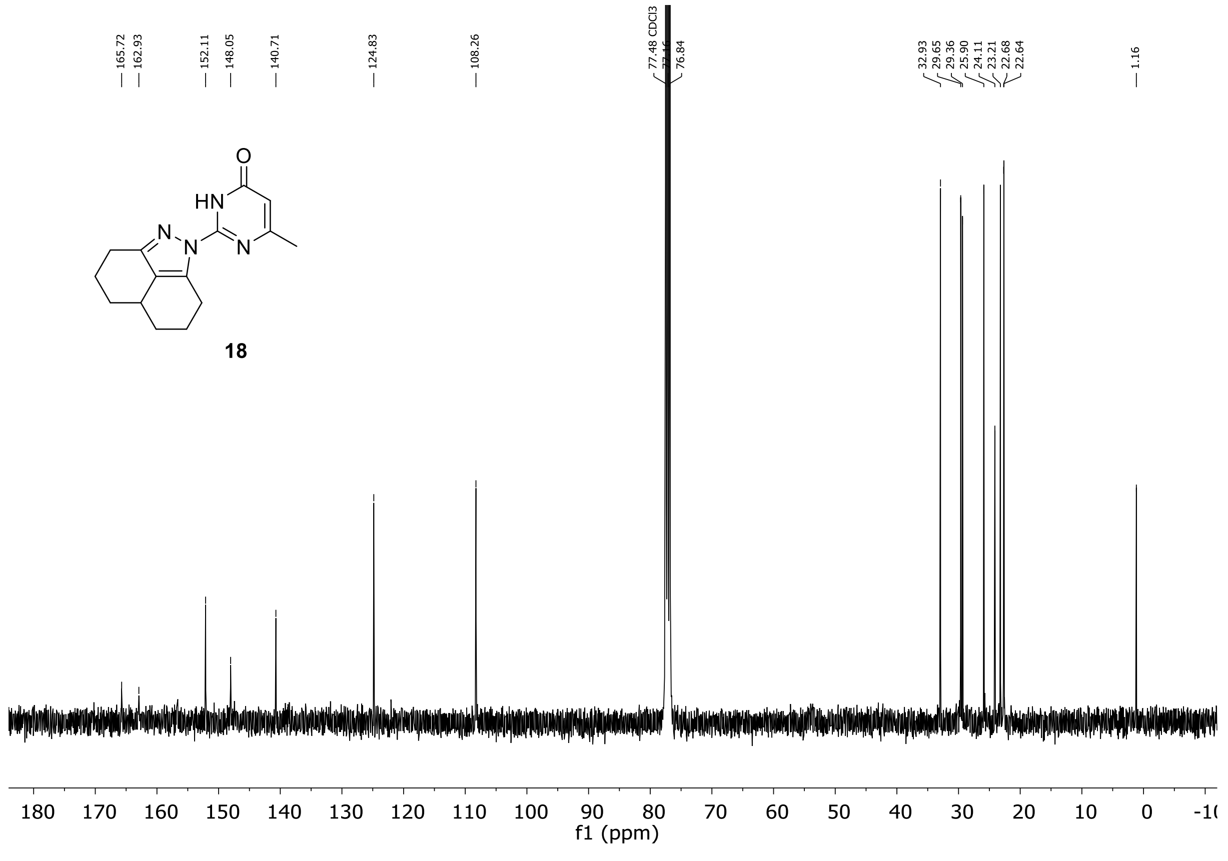
165.72
162.93
152.11
148.05
140.71
124.83
108.26
77.48 CDCl3
76.84
32.93
29.65
29.36
25.90
24.11
23.21
22.68
22.64
1.16
O
HN
N
N
N
18
180
170
160
150
140
130
120
110
100
90
80
70
60
50
40
30
20
10
0
f1 (ppm)

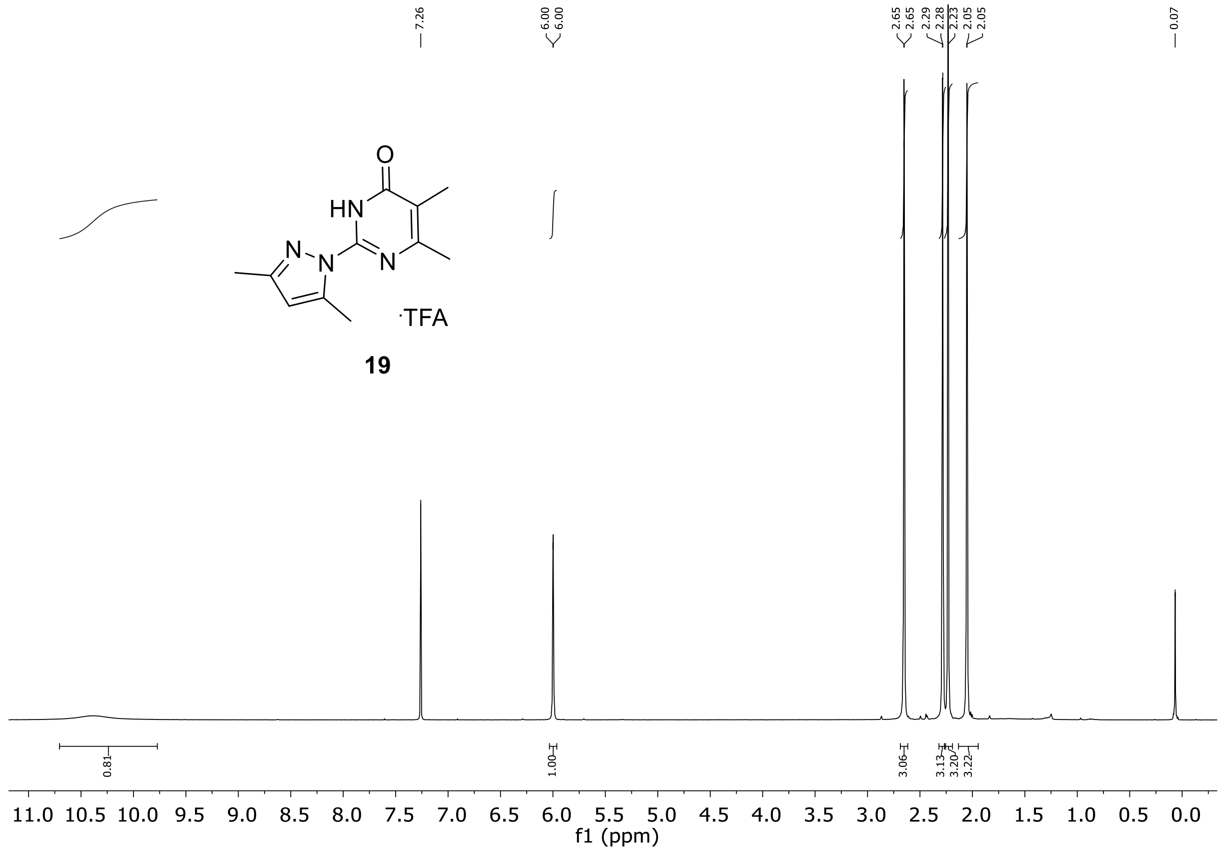
7.26
6.00
6.00
2.65
2.65
2.29
2.28
2.23
2.05
2.05
0.07
O
HN
N
N
N
·TFA
19
0.81
1.00
3.06
3.13
3.20
3.22
11.0 10.5 10.0 9.5 9.0 8.5 8.0 7.5 7.0 6.5 6.0 5.5 5.0 4.5 4.0 3.5 3.0 2.5 2.0 1.5 1.0 0.5 0.0
f1 (ppm)

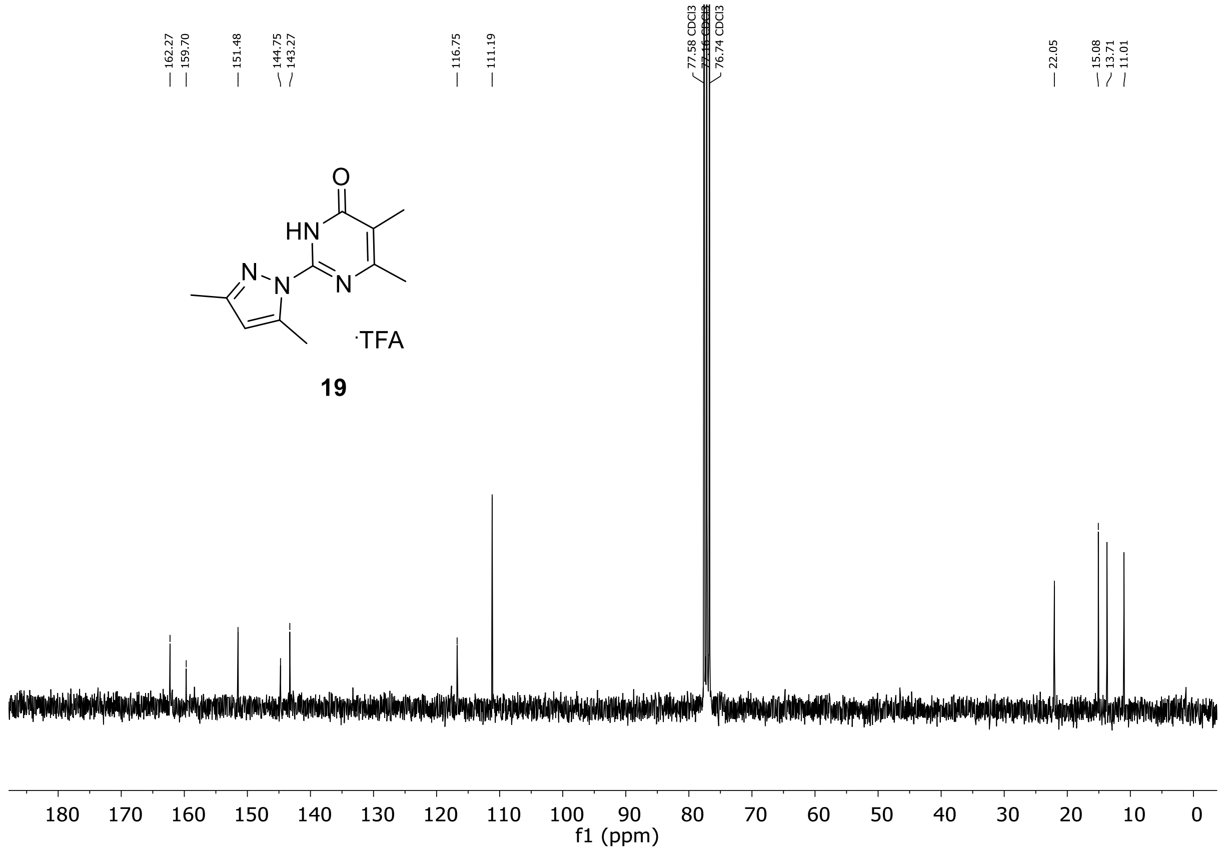
162.27
159.70
151.48
144.75
143.27
116.75
111.19
77.58 CDCl3
76.74 CDCl3
22.05
15.08
13.71
11.01
O
HN
N
N
N
·TFA
19
180 170 160 150 140 130 120 110 100 90 80 70 60 50 40 30 20 10 0
f1 (ppm)

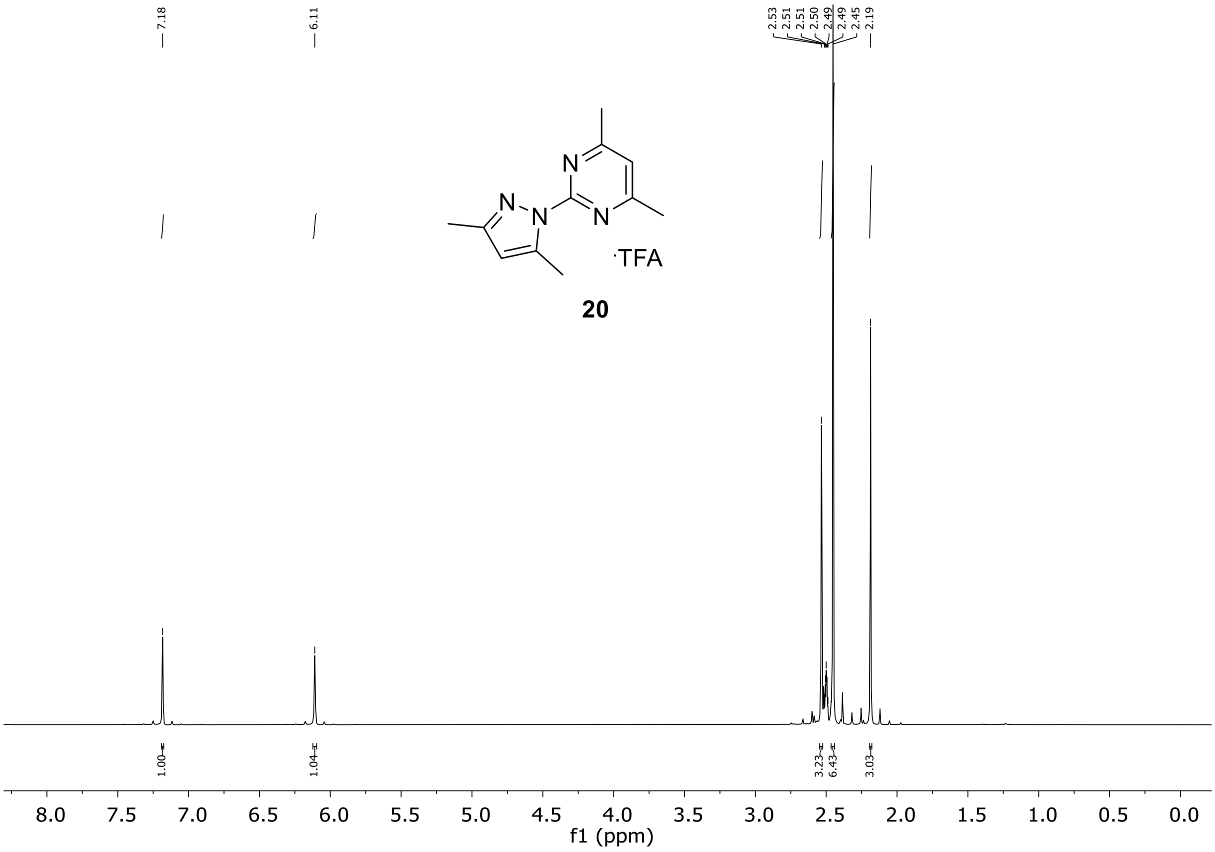
7.18
6.11
2.53
2.51
2.51
2.50
2.49
2.49
2.45
2.19
N
N
N
N
·TFA
20
1.00
1.04
3.23
6.43
3.03
8.0 7.5 7.0 6.5 6.0 5.5 5.0 4.5 4.0 3.5 3.0 2.5 2.0 1.5 1.0 0.5 0.0
f1 (ppm)

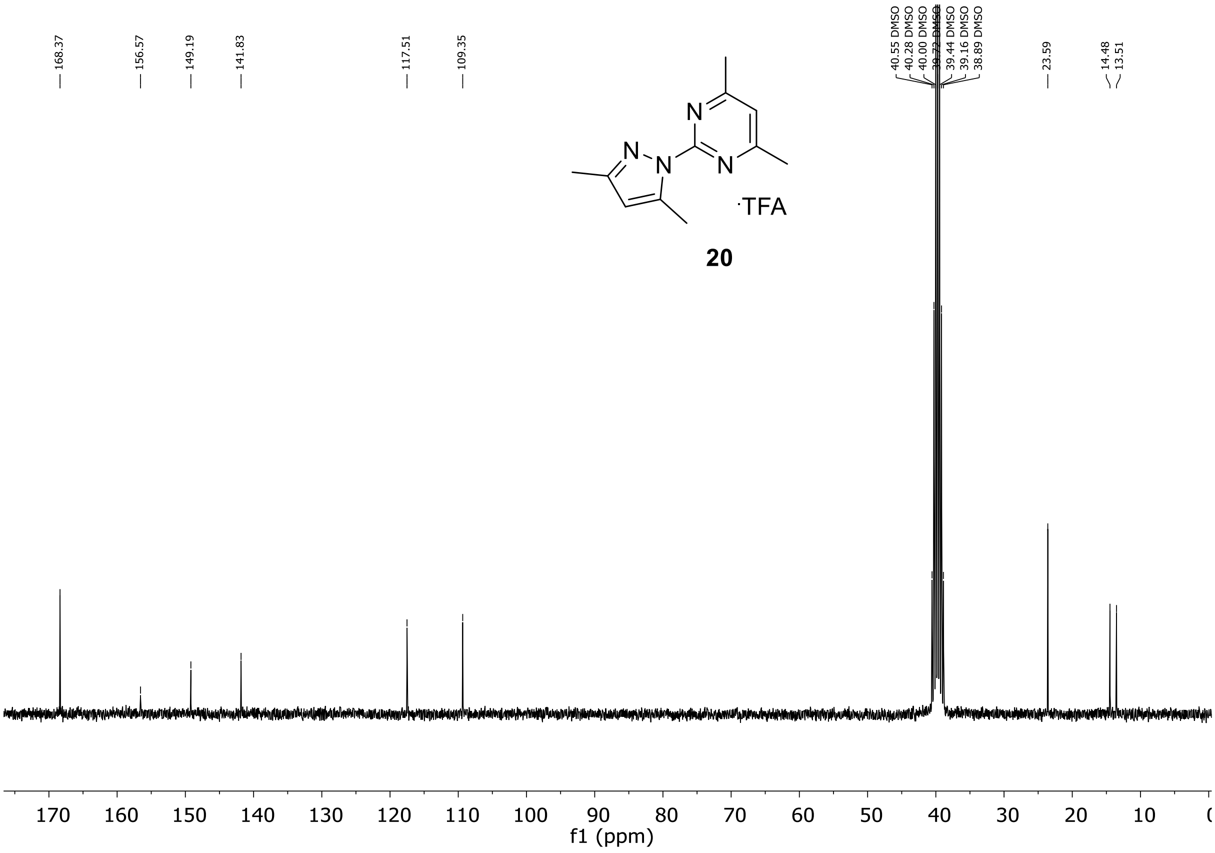
168.37
156.57
149.19
141.83
117.51
109.35
40.55 DMSO
40.28 DMSO
40.00 DMSO
39.44 DMSO
39.16 DMSO
38.89 DMSO
23.59
14.48
13.51
N
N
N
N
·TFA
20
170 160 150 140 130 120 110 100 90 80 70 60 50 40 30 20 10
f1 (ppm)

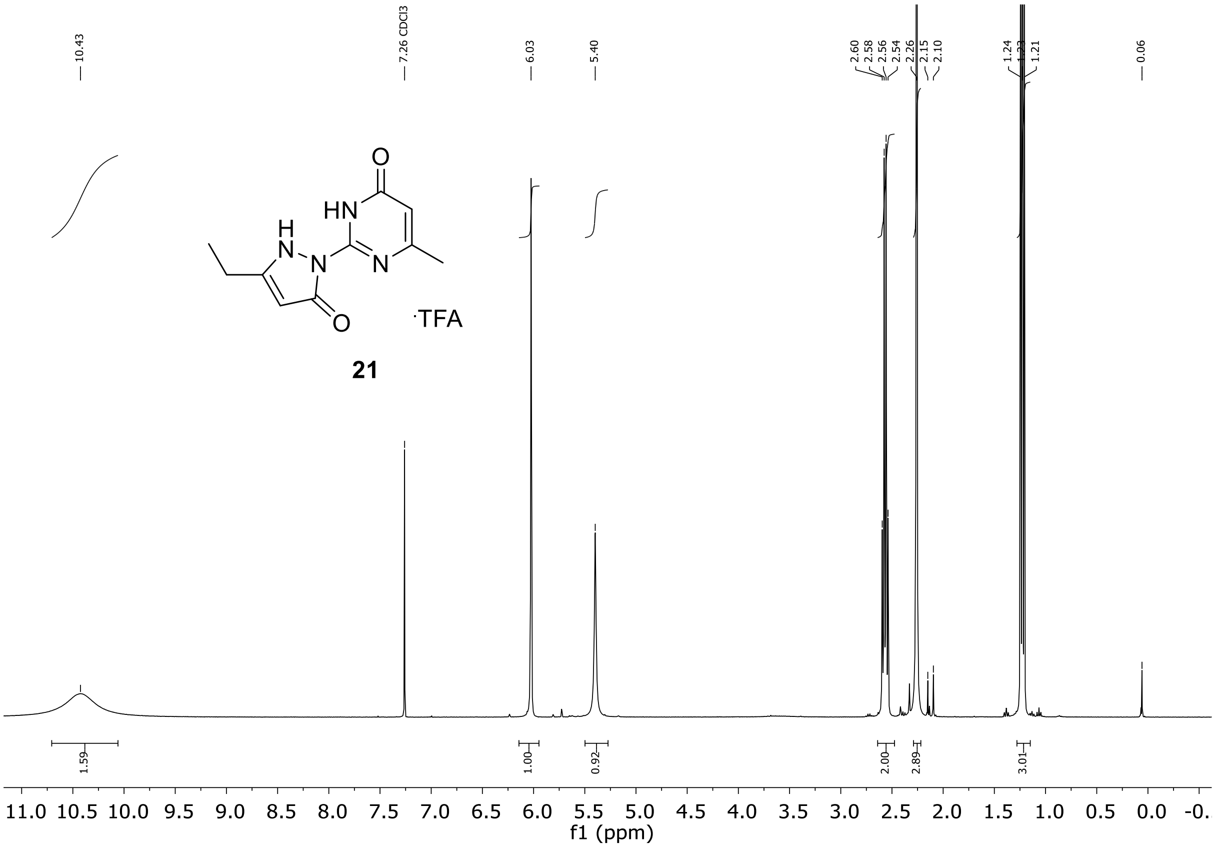
10.43
7.26 CDCl3
6.03
5.40
2.60
2.58
2.56
2.54
2.26
2.15
2.10
1.24
1.21
0.06
HN
O
N
H
N
N
O
·TFA
21
1.59
1.00
0.92
2.00
2.89
3.01
11.0 10.5 10.0 9.5 9.0 8.5 8.0 7.5 7.0 6.5 6.0 5.5 5.0 4.5 4.0 3.5 3.0 2.5 2.0 1.5 1.0 0.5 0.0 -0.
f1 (ppm)

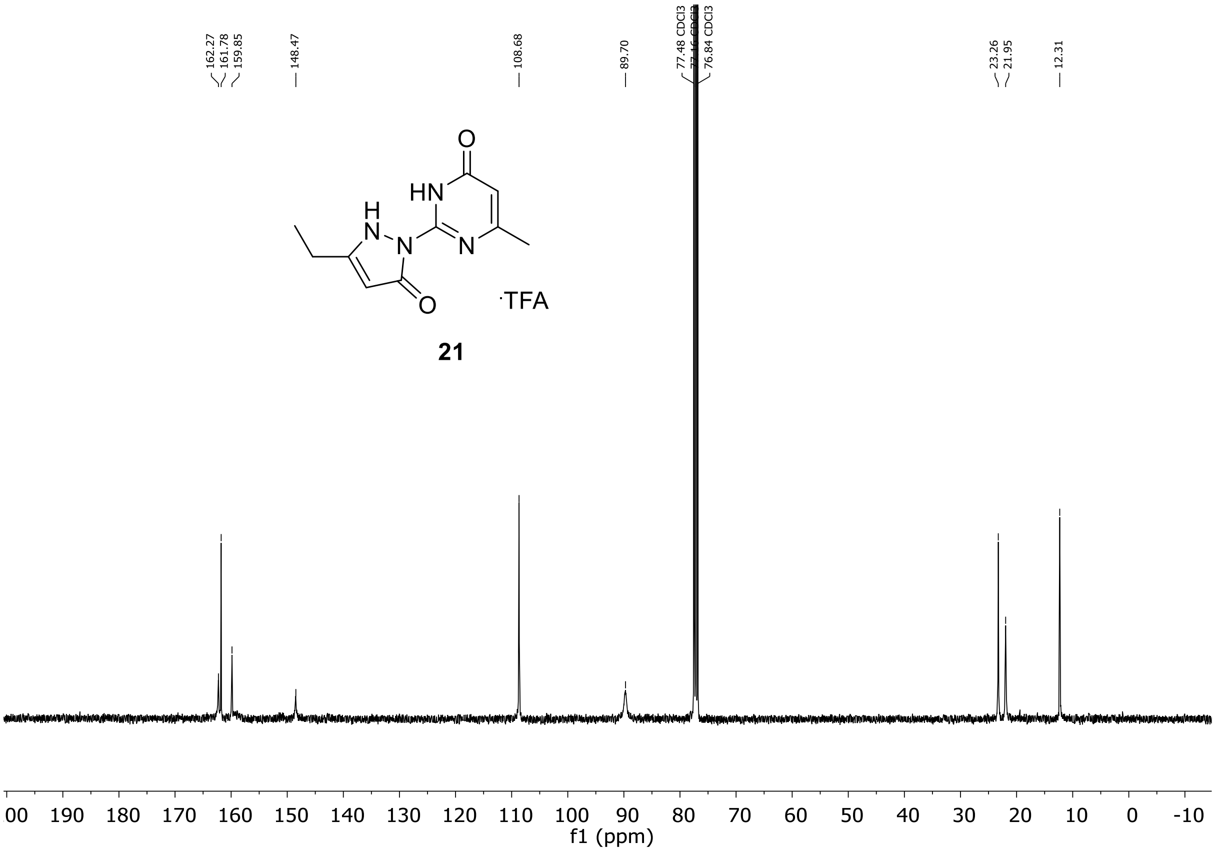
162.27
161.78
159.85
148.47
108.68
89.70
77.48 CDCl3
76.84 CDCl3
23.26
21.95
12.31
HN
O
N
H
N
N
O
·TFA
21
00 190 180 170 160 150 140 130 120 110 100 90 80 70 60 50 40 30 20 10 0 -10
f1 (ppm)

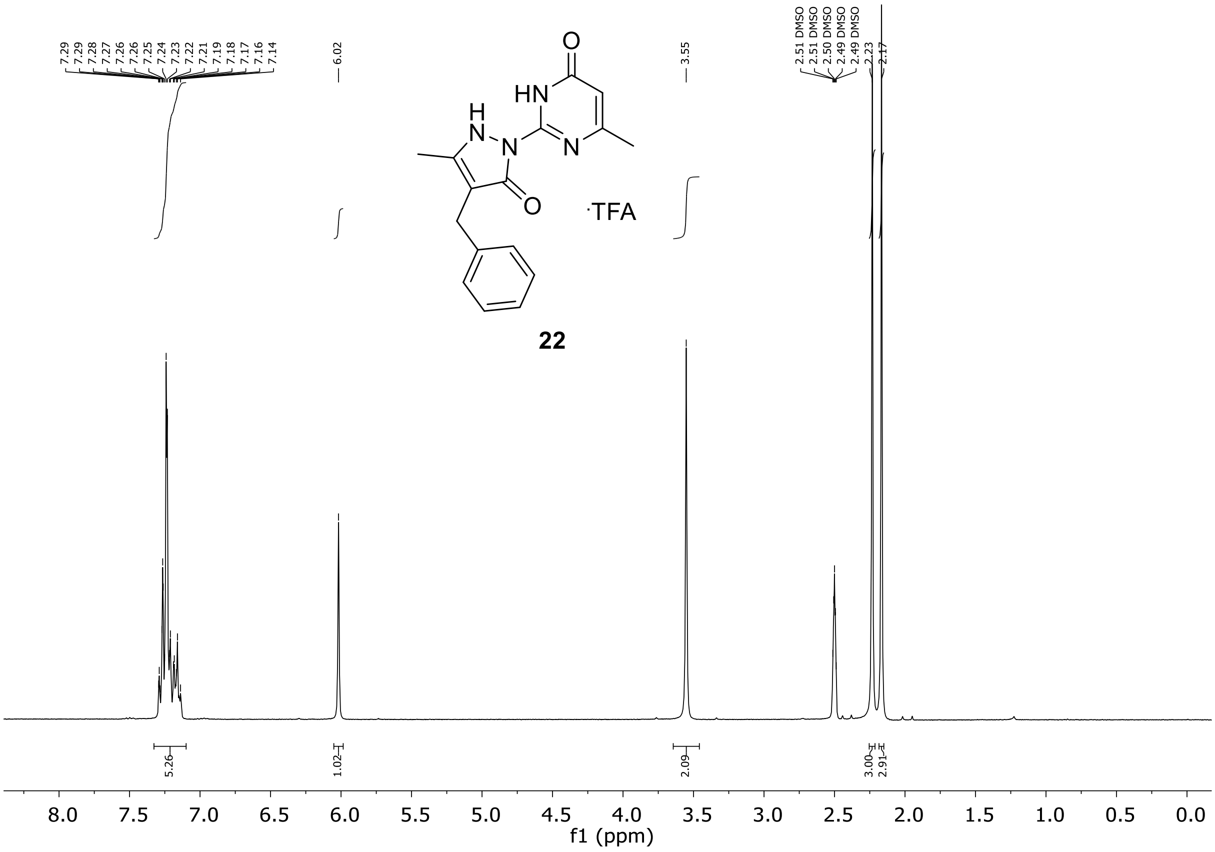
7.29
7.29
7.28
7.27
7.26
7.26
7.25
7.24
7.23
7.22
7.21
7.19
7.18
7.17
7.16
7.14
6.02
3.55
2.51 DMSO
2.51 DMSO
2.50 DMSO
2.49 DMSO
2.49 DMSO
2.23
2.17
HN
H
N
N
N
O
O
·TFA
22
5.26
1.02
2.09
3.00
2.91
8.0 7.5 7.0 6.5 6.0 5.5 5.0 4.5 4.0 3.5 3.0 2.5 2.0 1.5 1.0 0.5 0.0
f1 (ppm)

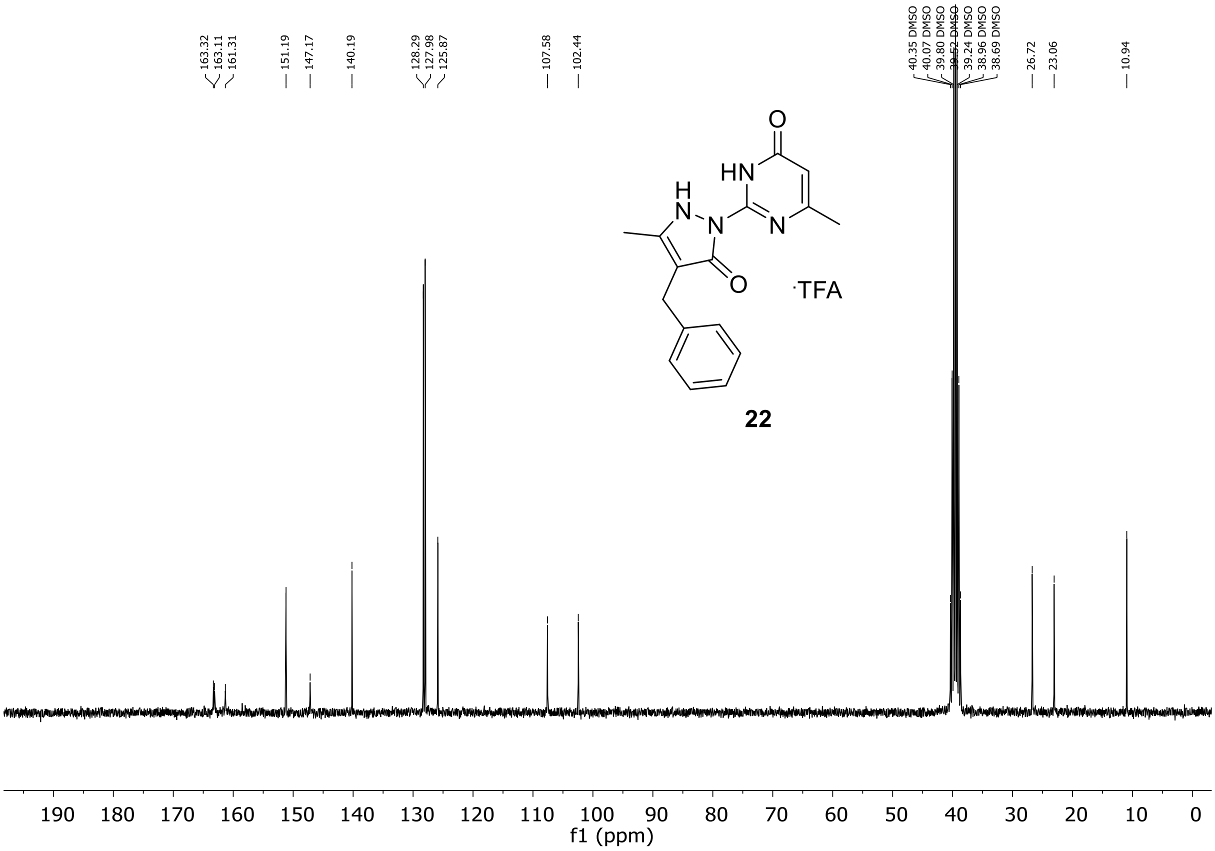
163.32
163.11
161.31
151.19
147.17
140.19
128.29
127.98
125.87
107.58
102.44
40.35 DMSO
40.07 DMSO
39.80 DMSO
39.52 DMSO
39.24 DMSO
38.96 DMSO
38.69 DMSO
26.72
23.06
10.94
HN
H
N
N
N
O
O
·TFA
22
190 180 170 160 150 140 130 120 110 100 90 80 70 60 50 40 30 20 10 0
f1 (ppm)

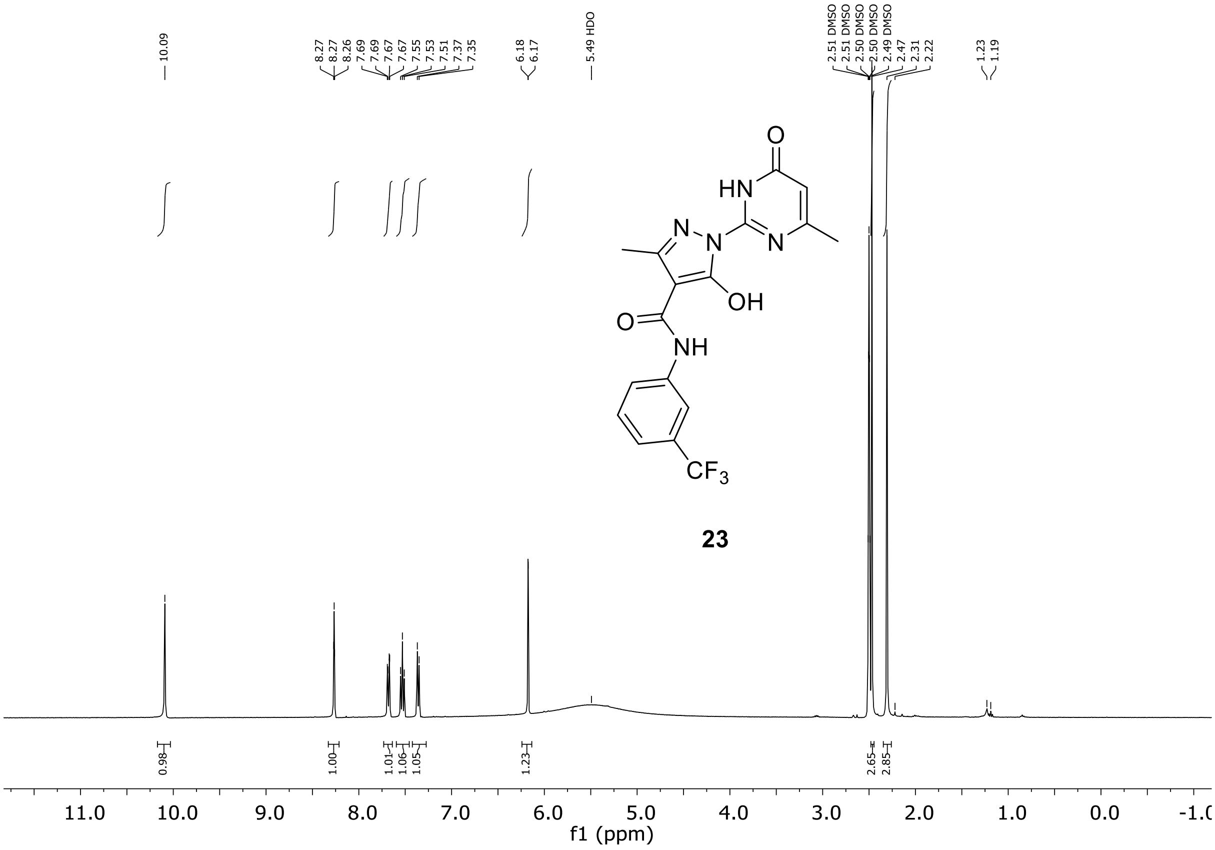
10.09
8.27 8.27 8.26 7.69 7.67 7.67 7.55 7.53 7.51 7.37 7.35
6.18 6.17
5.49 HDO
2.51 DMSO 2.51 DMSO 2.50 DMSO 2.50 DMSO 2.49 DMSO 2.47 2.31 2.22
1.23 1.19
HN
O
N
N
N
OH
O
NH
CF3
23
0.98 1.00 1.01 1.06 1.05 1.23 2.65 2.85
11.0 10.0 9.0 8.0 7.0 6.0 5.0 4.0 3.0 2.0 1.0 0.0
f1 (ppm)

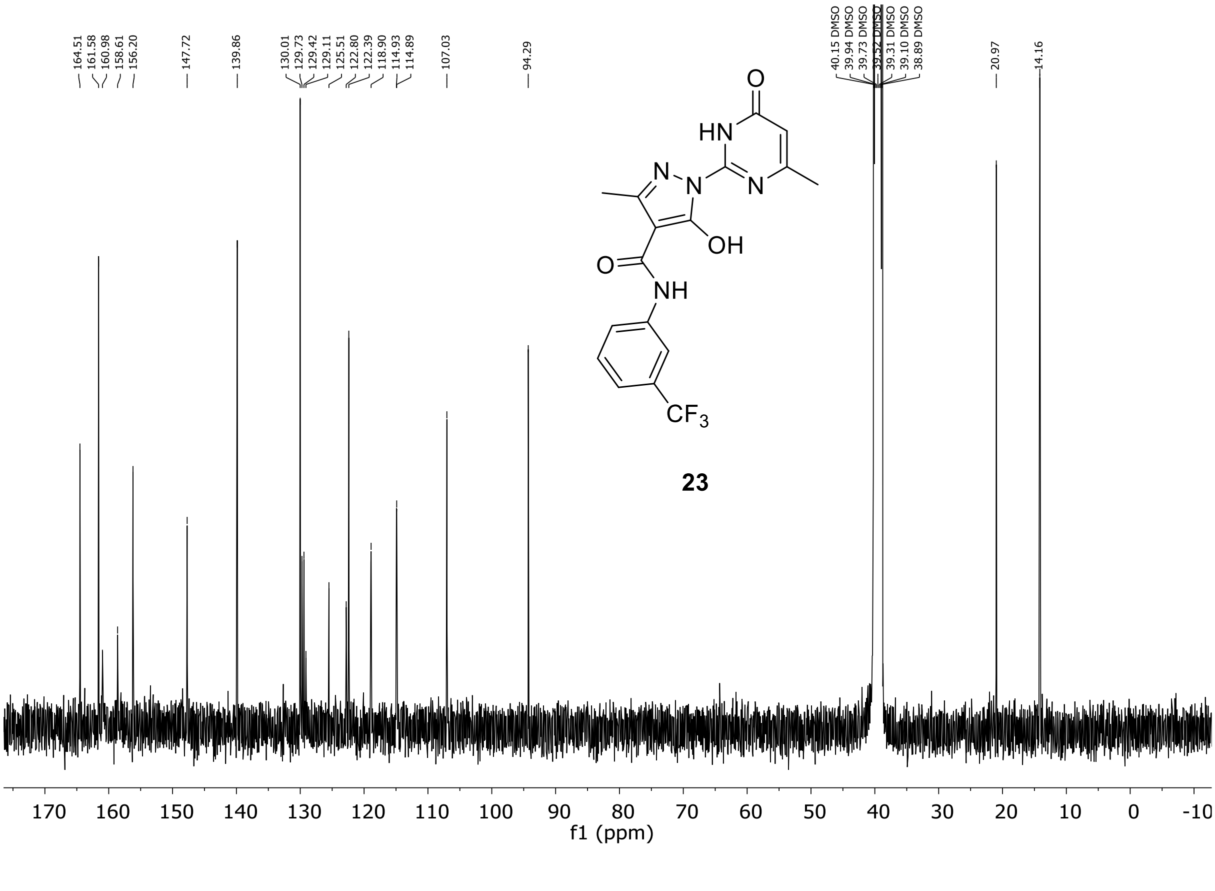
164.51 161.58 160.98 158.61 156.20
147.72
139.86
130.01 129.73 129.42 129.11 125.51 122.80 122.39 118.90 114.93 114.89
107.03
94.29
40.15 DMSO 39.94 DMSO 39.73 DMSO 39.52 DMSO 39.31 DMSO 39.10 DMSO 38.89 DMSO
20.97
14.16
HN
O
N
N
N
OH
O
NH
CF3
23
170 160 150 140 130 120 110 100 90 80 70 60 50 40 30 20 10 0 -10
f1 (ppm)

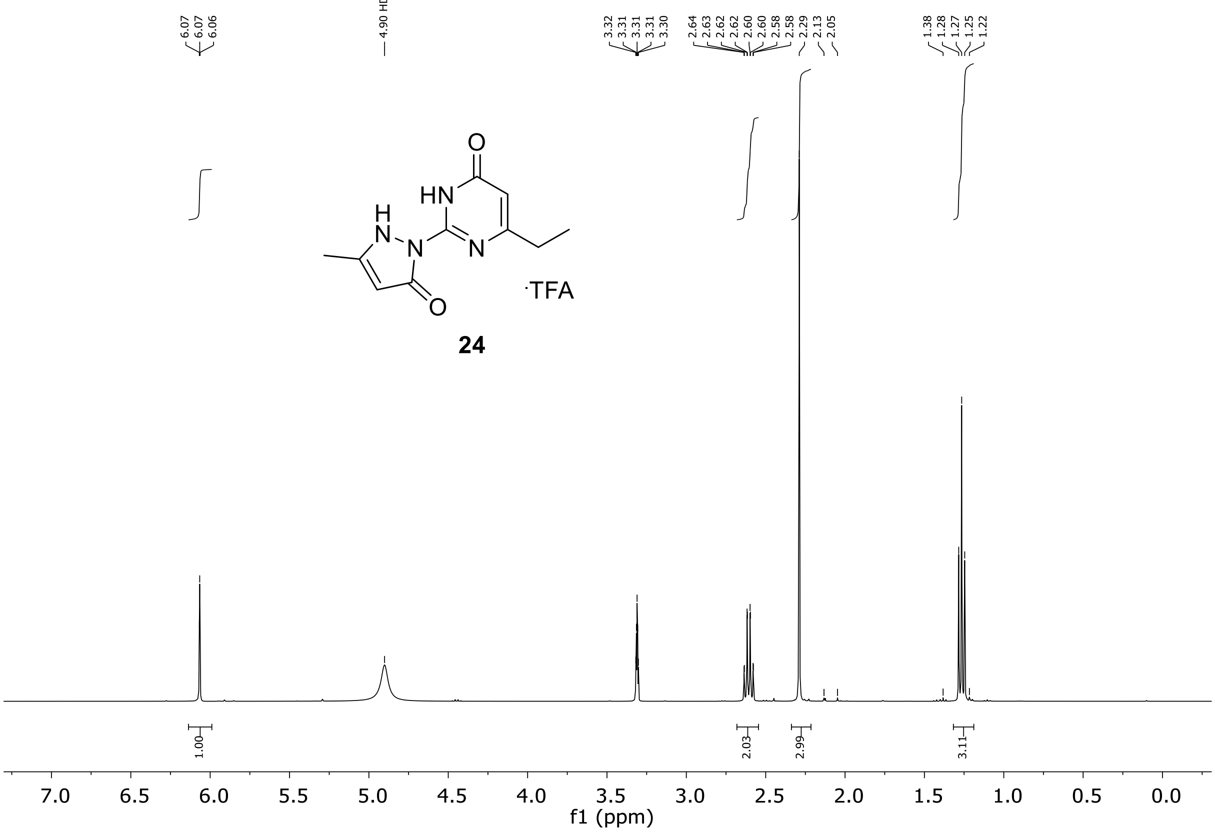
6.07
6.07
6.06
4.90 HDO
3.32
3.31
3.31
3.31
3.30
2.64
2.63
2.62
2.62
2.60
2.60
2.58
2.58
2.29
2.13
2.05
1.38
1.28
1.27
1.25
1.22
HN
H
N
N
N
O
O
·TFA
24
1.00
2.03
2.99
3.11
7.0 6.5 6.0 5.5 5.0 4.5 4.0 3.5 3.0 2.5 2.0 1.5 1.0 0.5 0.0
f1 (ppm)

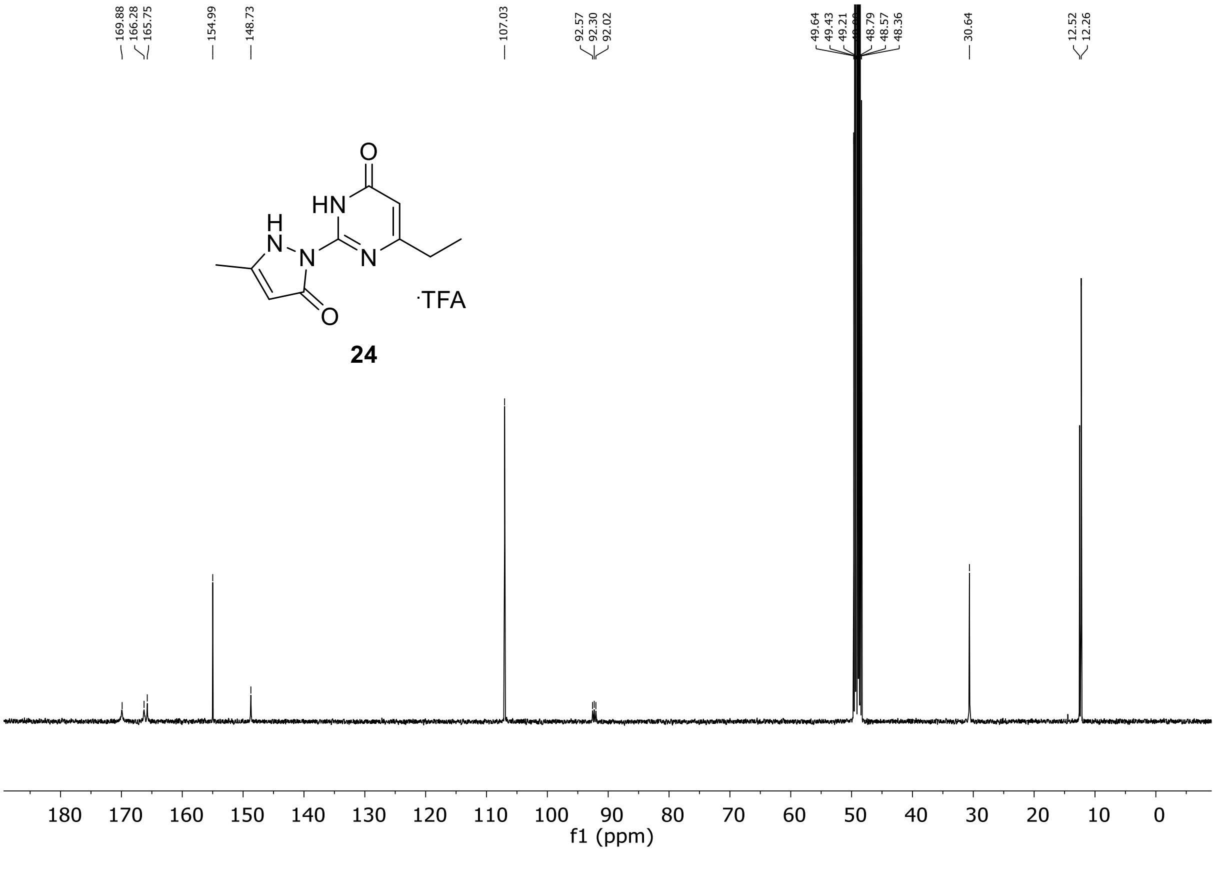
169.88
166.28
165.75
154.99
148.73
107.03
92.57
92.30
92.02
49.64
49.43
49.21
48.79
48.57
48.36
30.64
12.52
12.26
HN
H
N
N
N
O
O
·TFA
24
180 170 160 150 140 130 120 110 100 90 80 70 60 50 40 30 20 10 0
f1 (ppm)

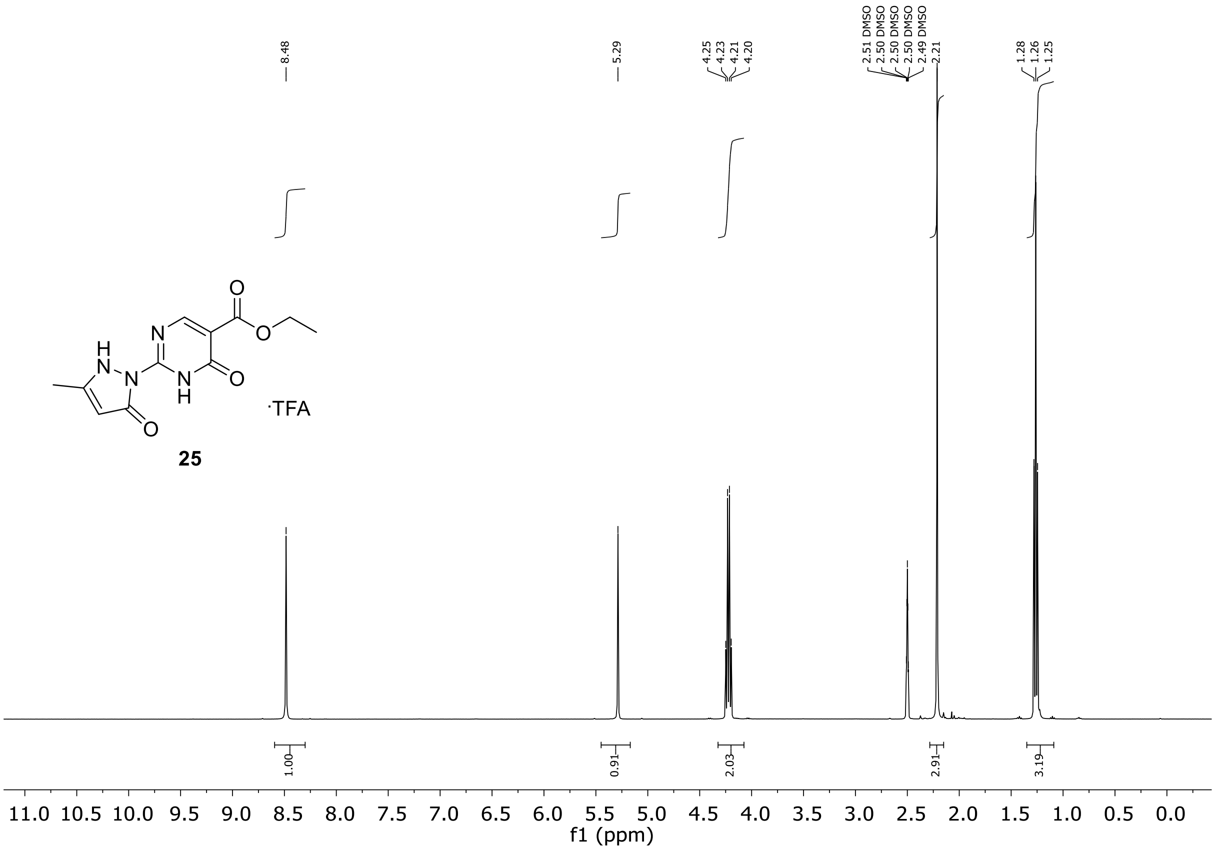
8.48
5.29
4.25
4.23
4.21
4.20
2.51 DMSO
2.50 DMSO
2.50 DMSO
2.50 DMSO
2.49 DMSO
2.21
1.28
1.26
1.25
·TFA
25
1.00
0.91
2.03
2.91
3.19
11.0 10.5 10.0 9.5 9.0 8.5 8.0 7.5 7.0 6.5 6.0 5.5 5.0 4.5 4.0 3.5 3.0 2.5 2.0 1.5 1.0 0.5 0.0
f1 (ppm)

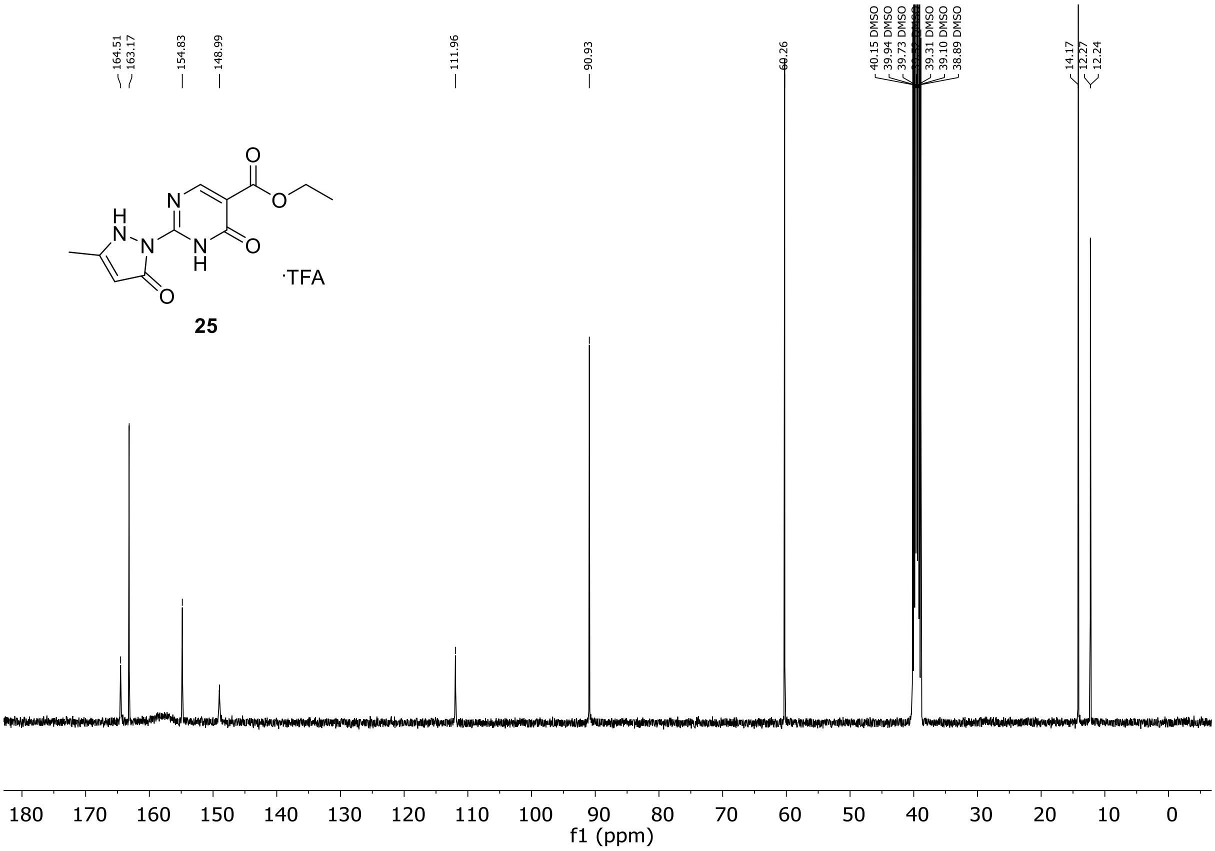
164.51
163.17
154.83
148.99
111.96
90.93
60.26
40.15 DMSO
39.94 DMSO
39.73 DMSO
39.31 DMSO
39.10 DMSO
38.89 DMSO
14.17
12.27
12.24
·TFA
25
180 170 160 150 140 130 120 110 100 90 80 70 60 50 40 30 20 10 0
f1 (ppm)

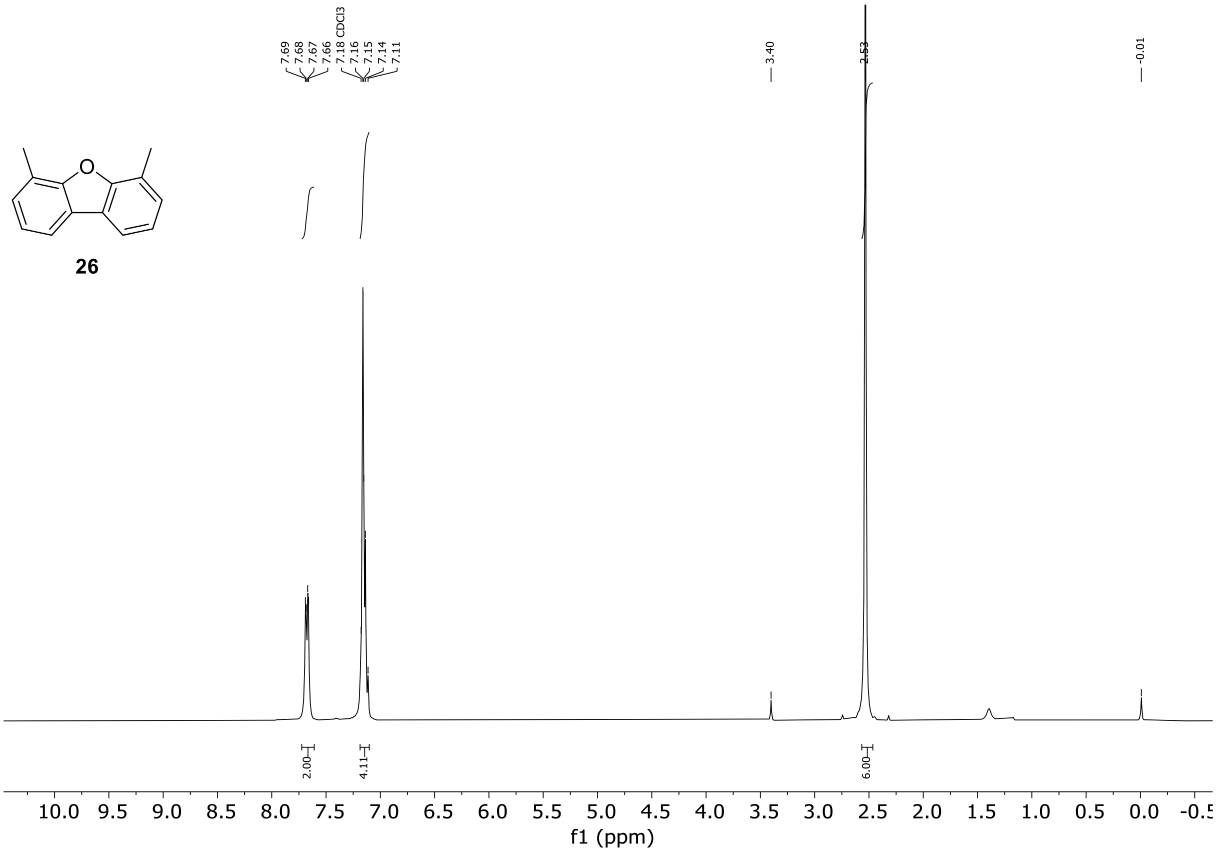
7.69
7.68
7.67
7.66
7.18 CDCl3
7.16
7.15
7.14
7.11
3.40
2.53
-0.01
O
26
2.00
4.11
6.00
10.0 9.5 9.0 8.5 8.0 7.5 7.0 6.5 6.0 5.5 5.0 4.5 4.0 3.5 3.0 2.5 2.0 1.5 1.0 0.5 0.0
f1 (ppm)

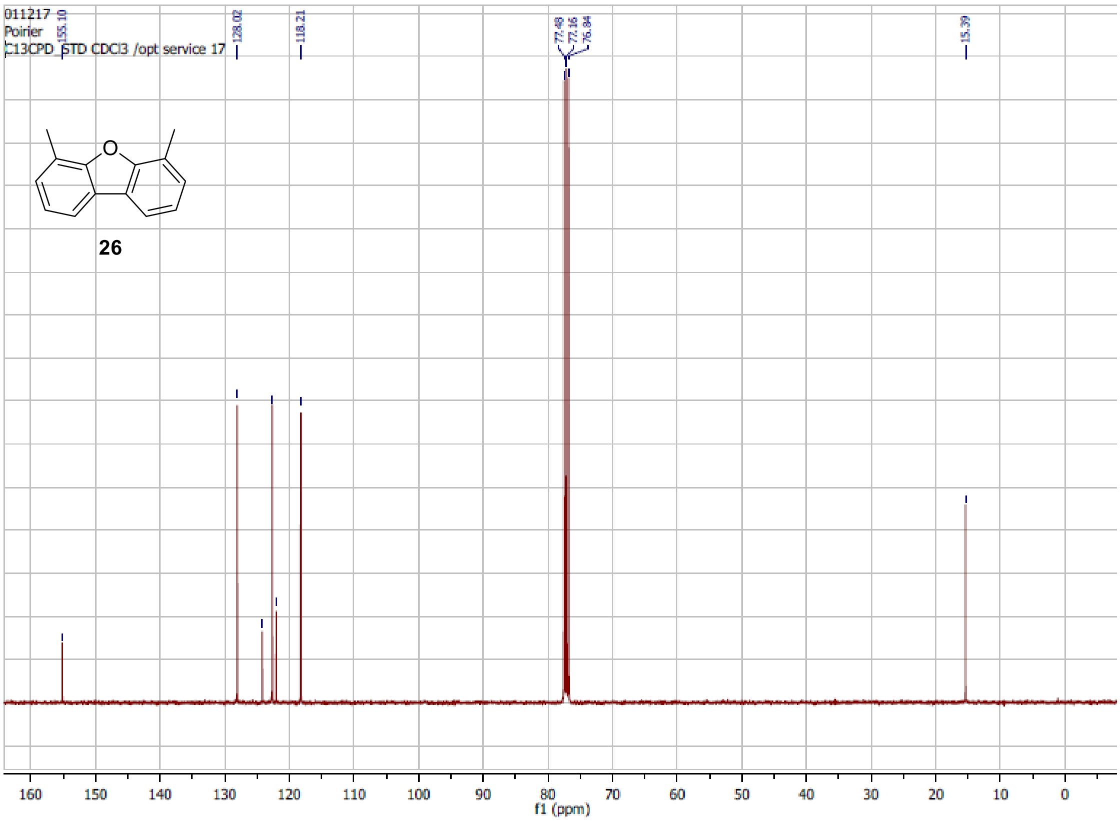
011217
Poirier
C13CPD_STD CDCl3 /opt service 17
155.10
128.02
118.21
77.48
77.16
76.84
15.39
O
26
160 150 140 130 120 110 100 90 80 70 60 50 40 30 20 10 0
f1 (ppm)

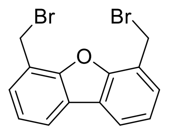

27

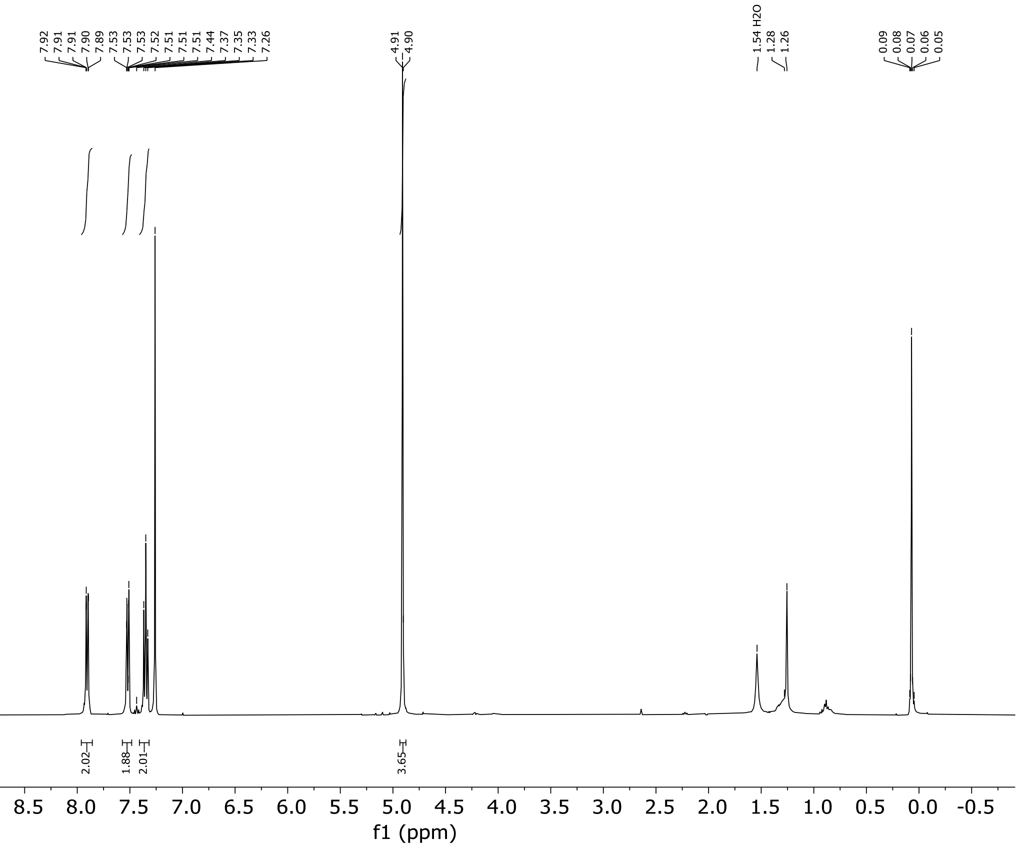
7.92
7.91
7.91
7.90
7.89
7.53
7.53
7.53
7.52
7.51
7.51
7.51
7.44
7.37
7.35
7.33
7.26
4.91
4.90
1.54 H2O
1.28
1.26
0.09
0.08
0.07
0.06
0.05
2.02
1.88
2.01
3.65
f1 (ppm)

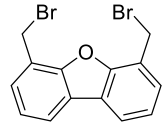

27

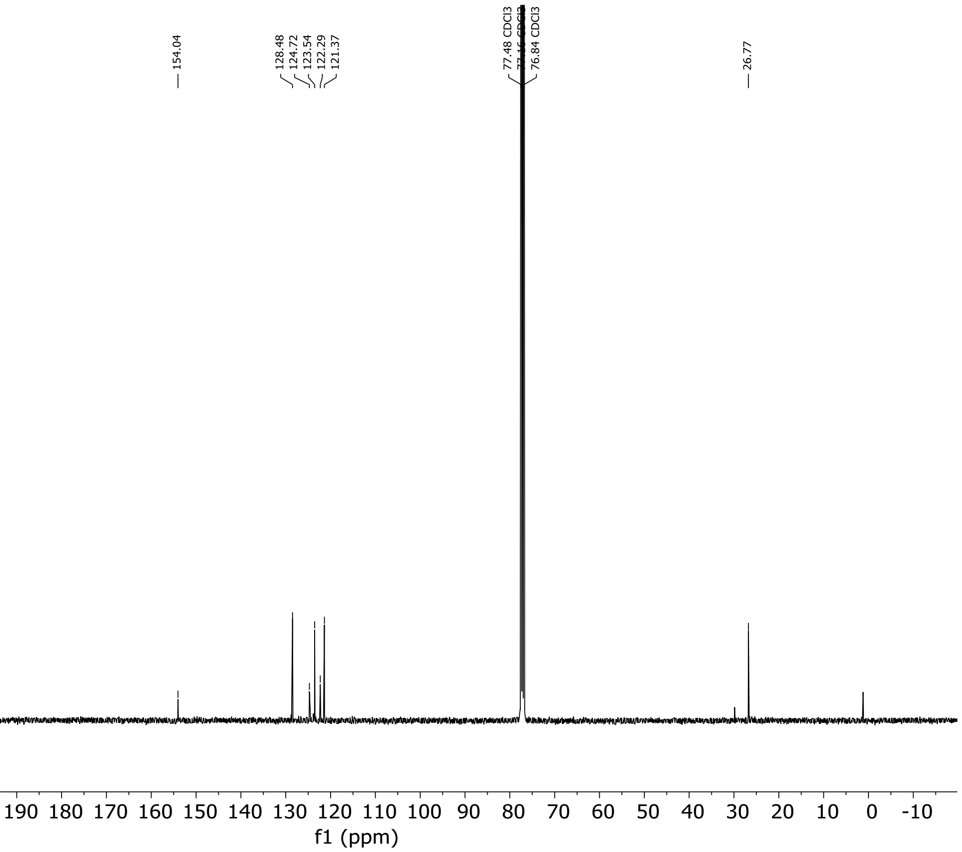
154.04
128.48
124.72
123.54
122.29
121.37
77.48 CDCl3
76.84 CDCl3
26.77
f1 (ppm)

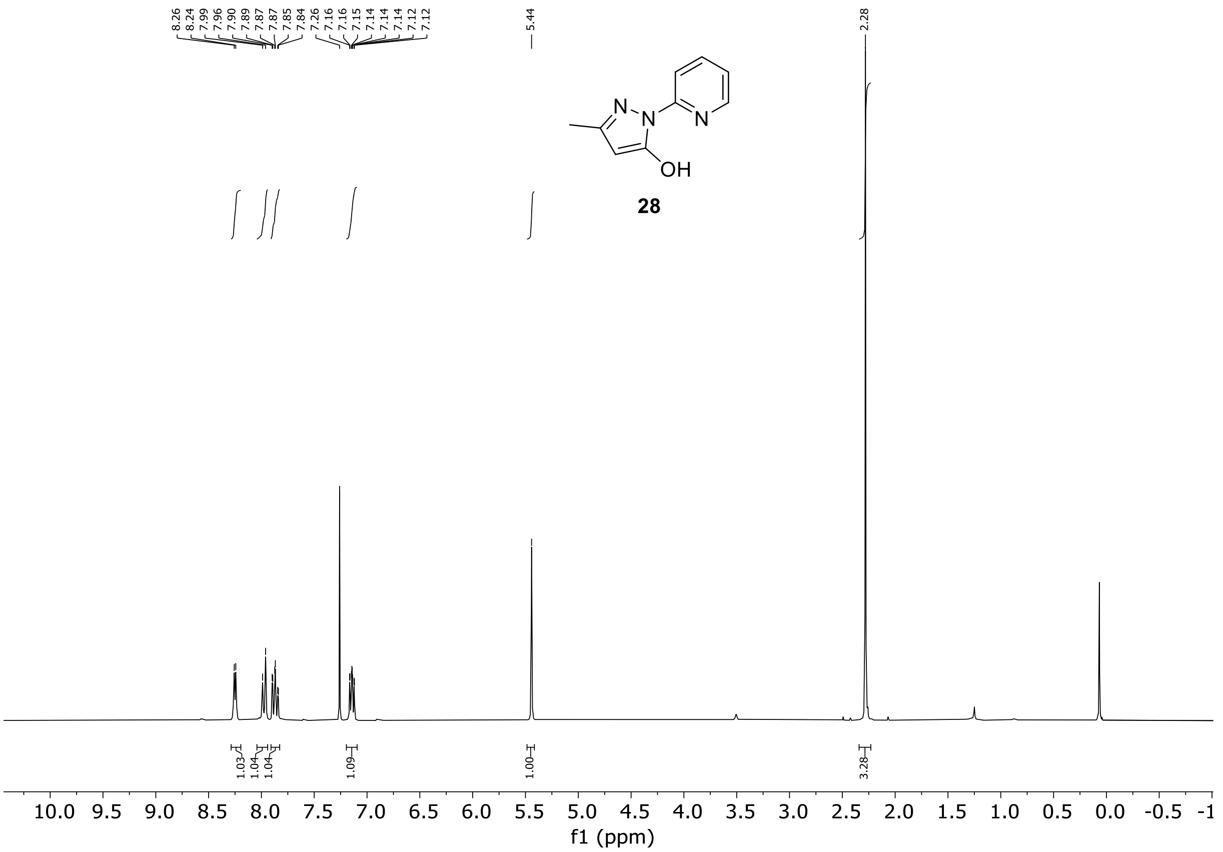

28

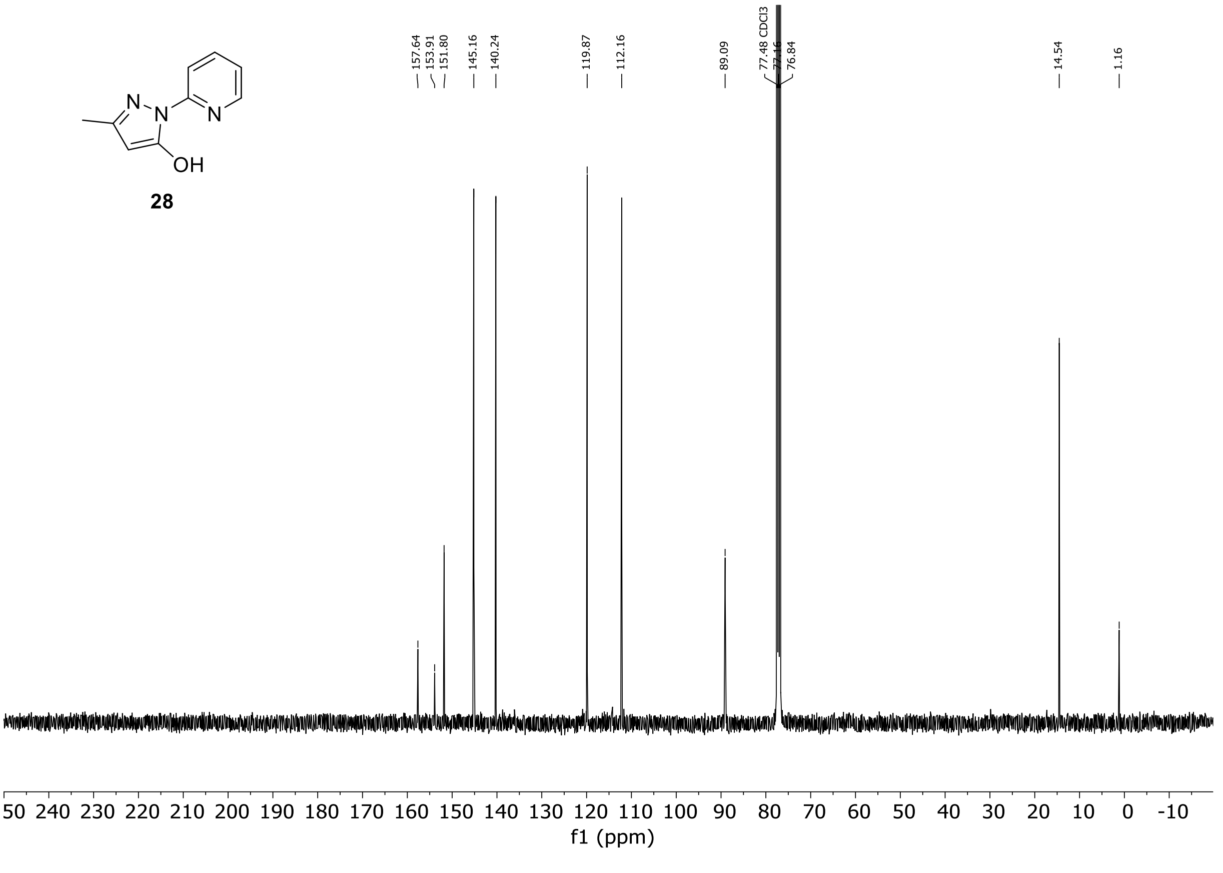

28

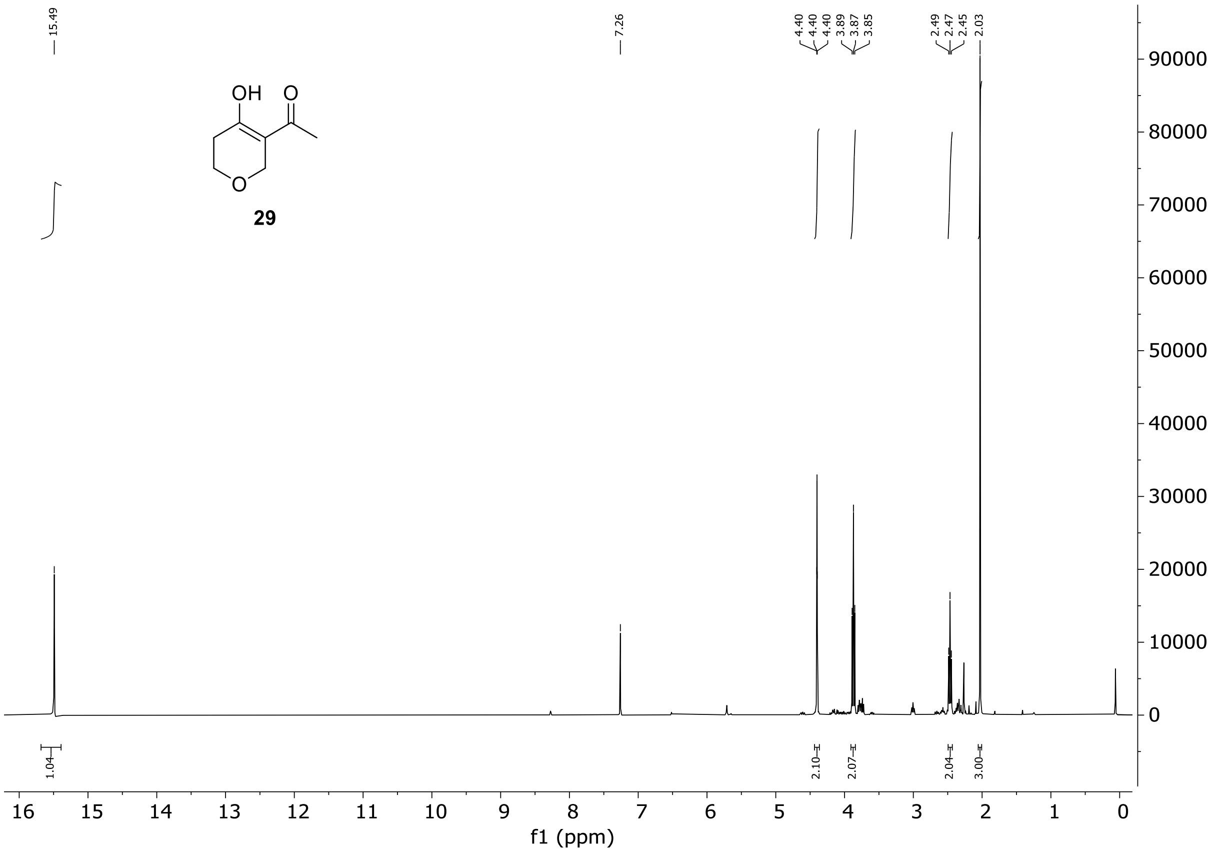
15.49
7.26
4.40
4.40
4.40
3.89
3.87
3.85
2.49
2.47
2.45
2.03
OH
O
O
29
1.04
2.10
2.07
2.04
3.00
f1 (ppm)

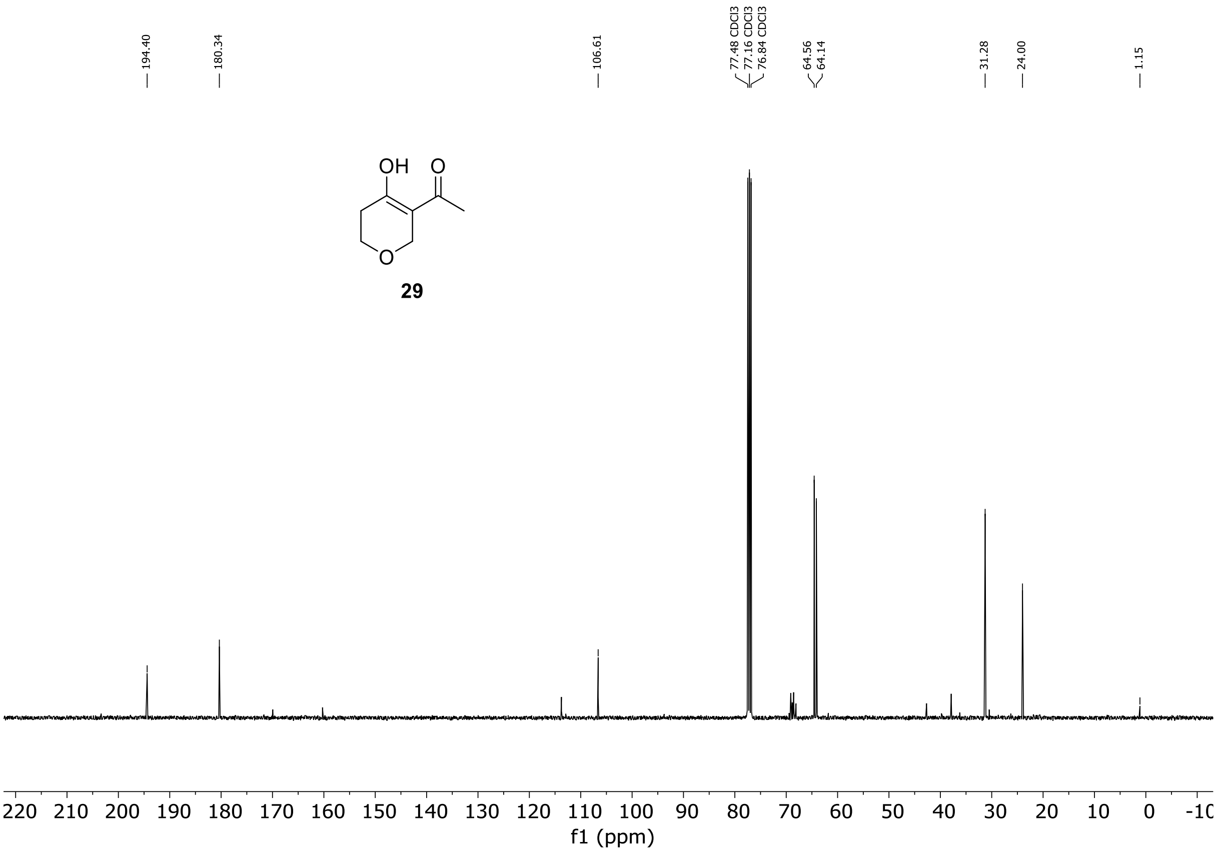
194.40
180.34
106.61
77.48 CDCl3
77.16 CDCl3
76.84 CDCl3
64.56
64.14
31.28
24.00
1.15
OH
O
O
29
f1 (ppm)

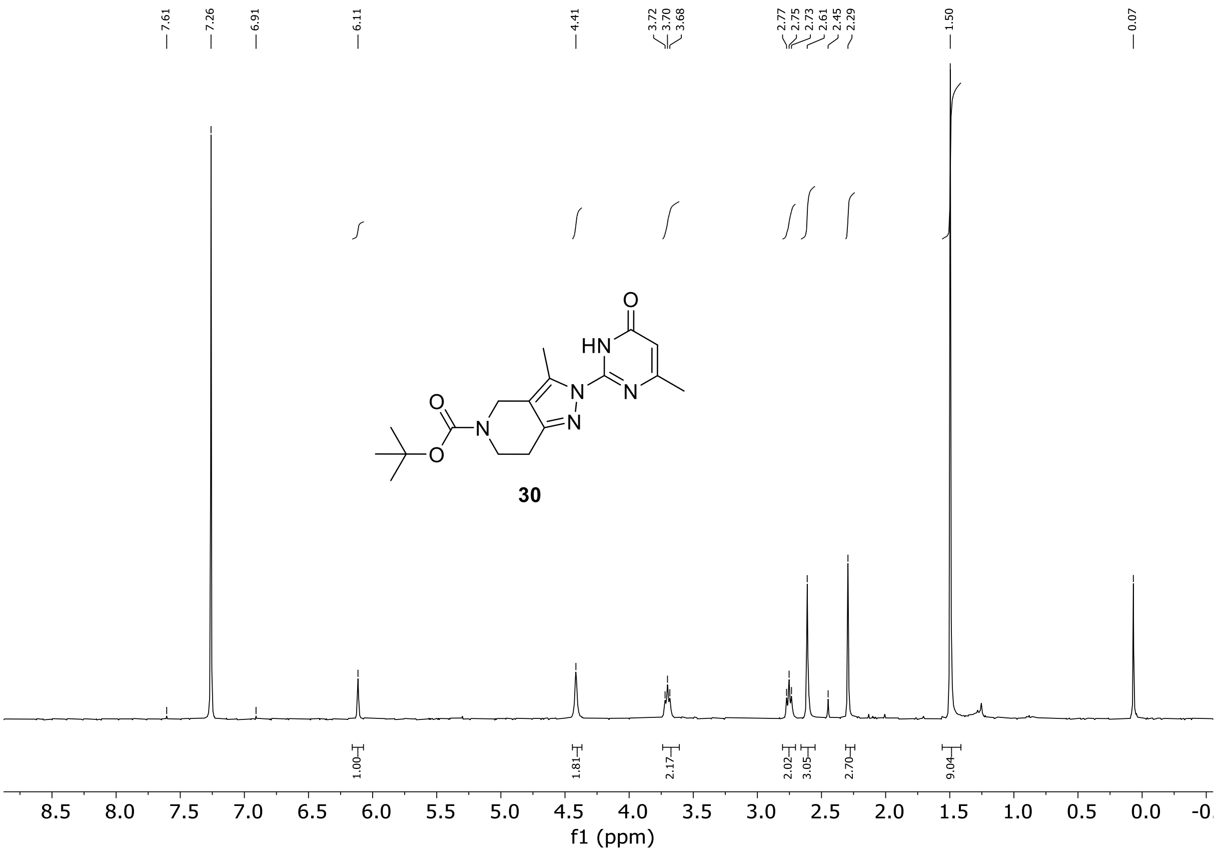
7.61
7.26
6.91
6.11
4.41
3.72
3.70
3.68
2.77
2.75
2.73
2.61
2.45
2.29
1.50
0.07
O
HN
N
N
N
O
O
30
1.00
1.81
2.17
2.02
3.05
2.70
9.04
f1 (ppm)

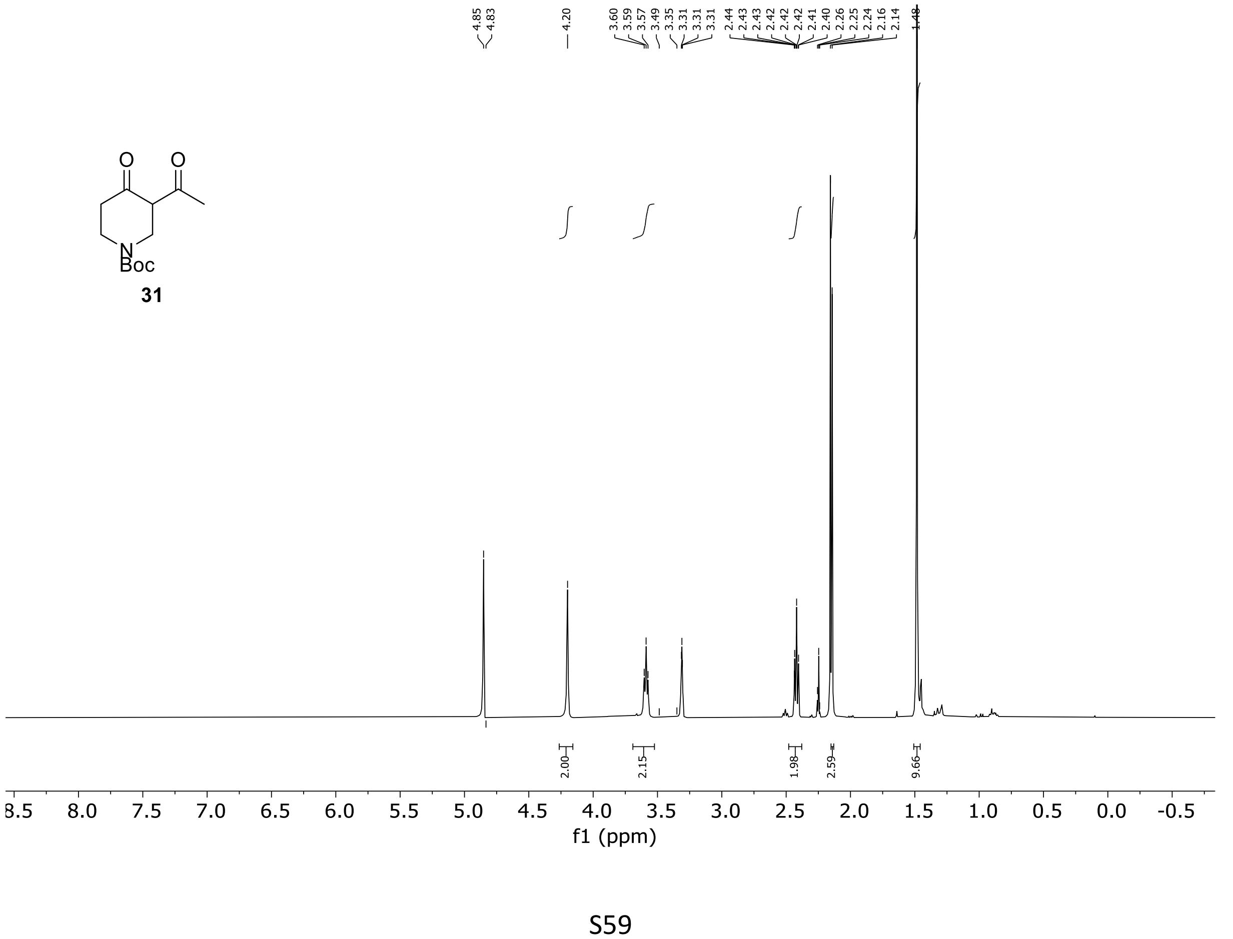
4.85
4.83
4.20
3.60
3.59
3.57
3.49
3.35
3.31
3.31
3.31
3.31
2.44
2.43
2.42
2.42
2.42
2.41
2.40
2.26
2.25
2.24
2.16
2.14
1.48
O
O
N
Boc
31
2.00
2.15
1.98
2.59
9.66
f1 (ppm)

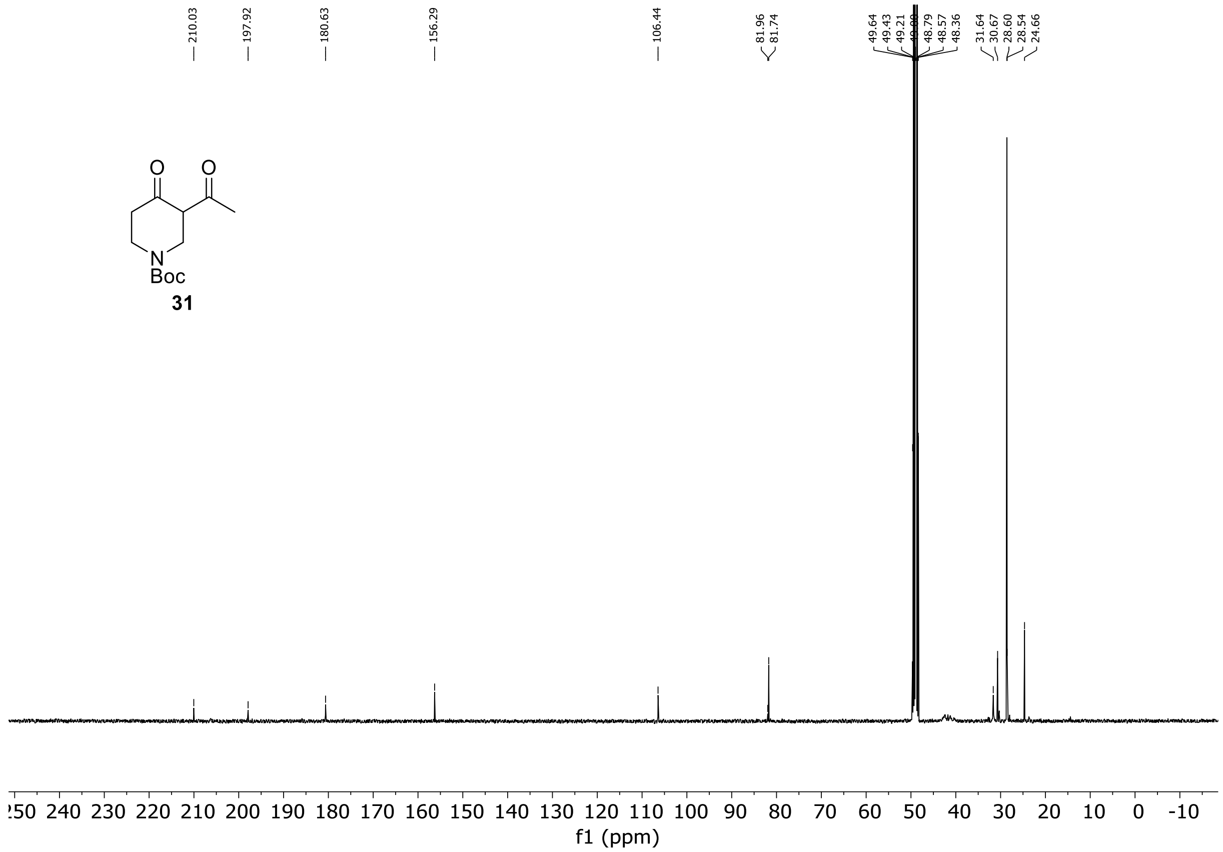
210.03
197.92
180.63
156.29
106.44
81.96
81.74
49.64
49.43
49.21
48.79
48.57
48.36
31.64
30.67
28.60
28.54
24.66
Boc
31
f1 (ppm)

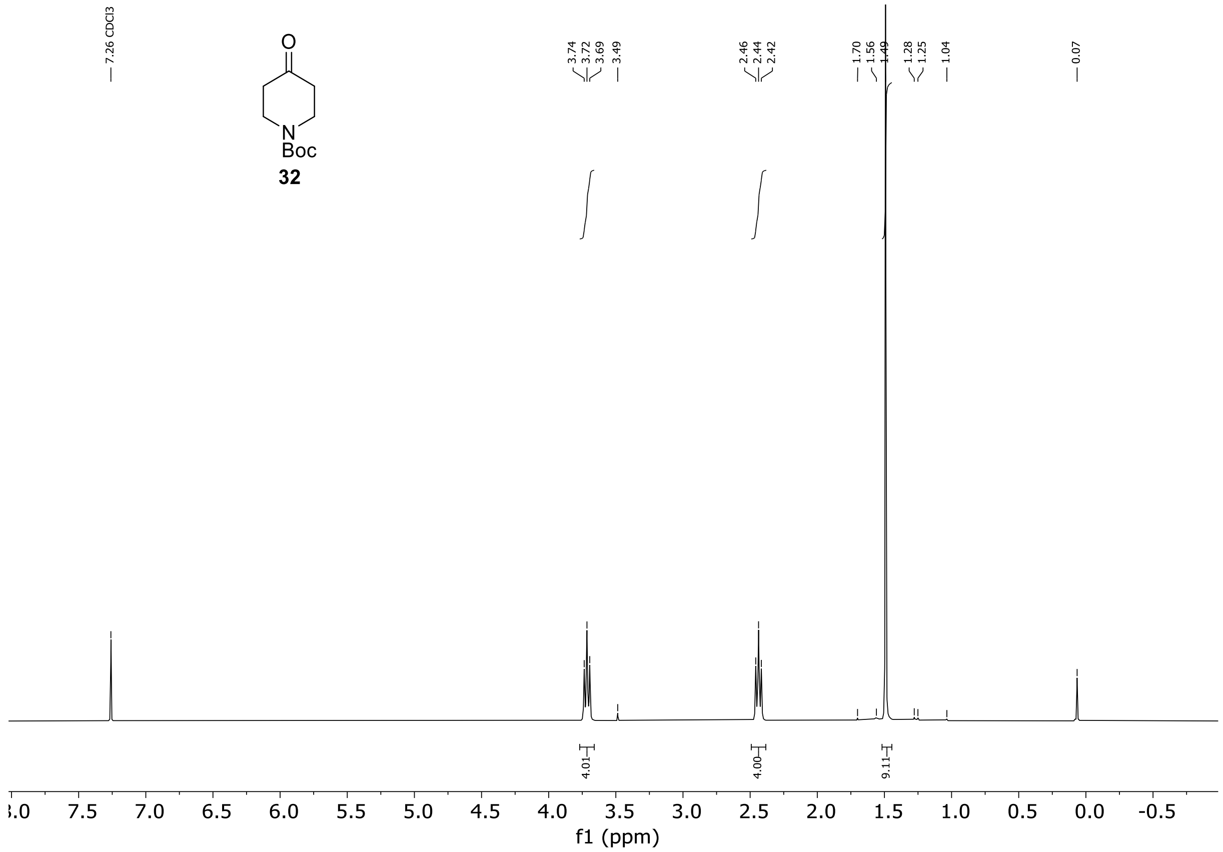
7.26 CDCl3
3.74
3.72
3.69
3.49
2.46
2.44
2.42
1.70
1.56
1.49
1.28
1.25
1.04
0.07
Boc
32
4.01
4.00
9.11
f1 (ppm)

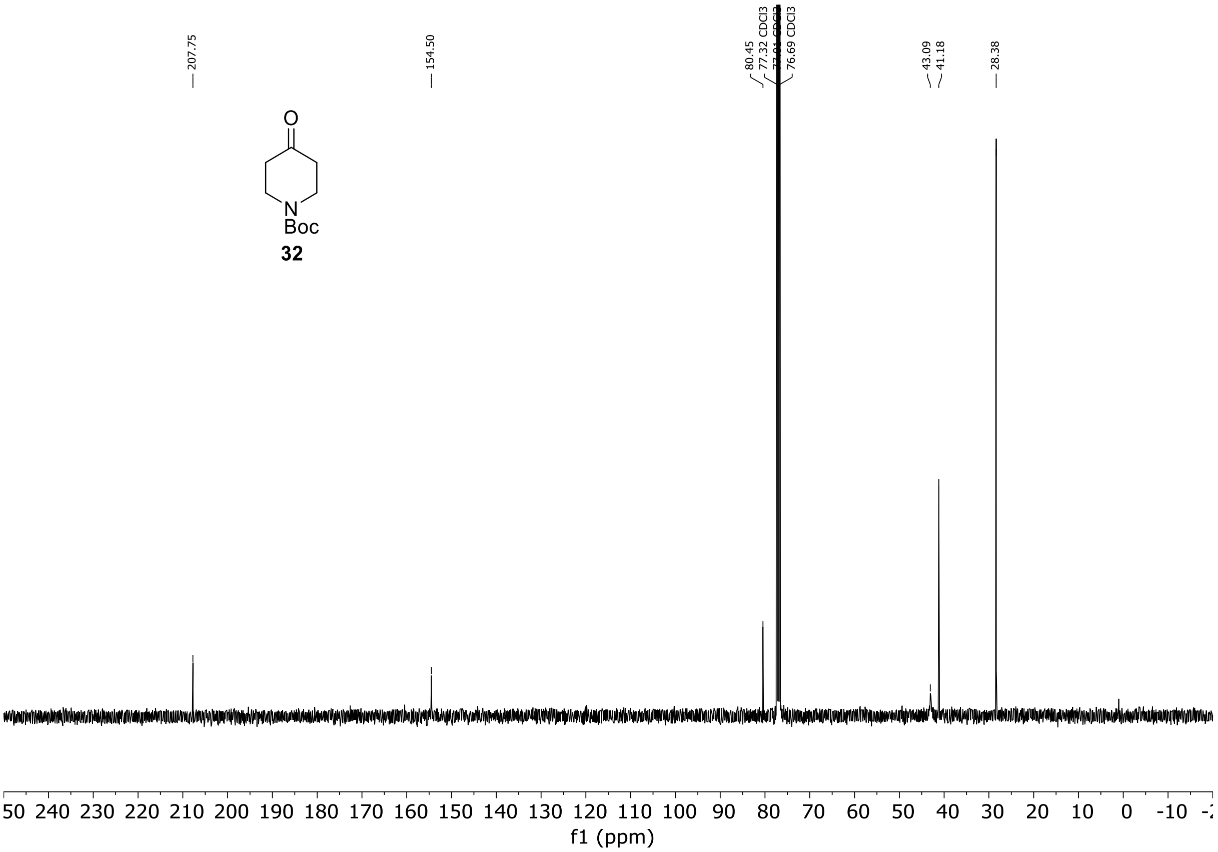
207.75
154.50
80.45
77.32 CDCl3
77.01 CDCl3
76.69 CDCl3
43.09
41.18
28.38
O
N
Boc
32
50 240 230 220 210 200 190 180 170 160 150 140 130 120 110 100 90 80 70 60 50 40 30 20 10 0 -10
f1 (ppm)

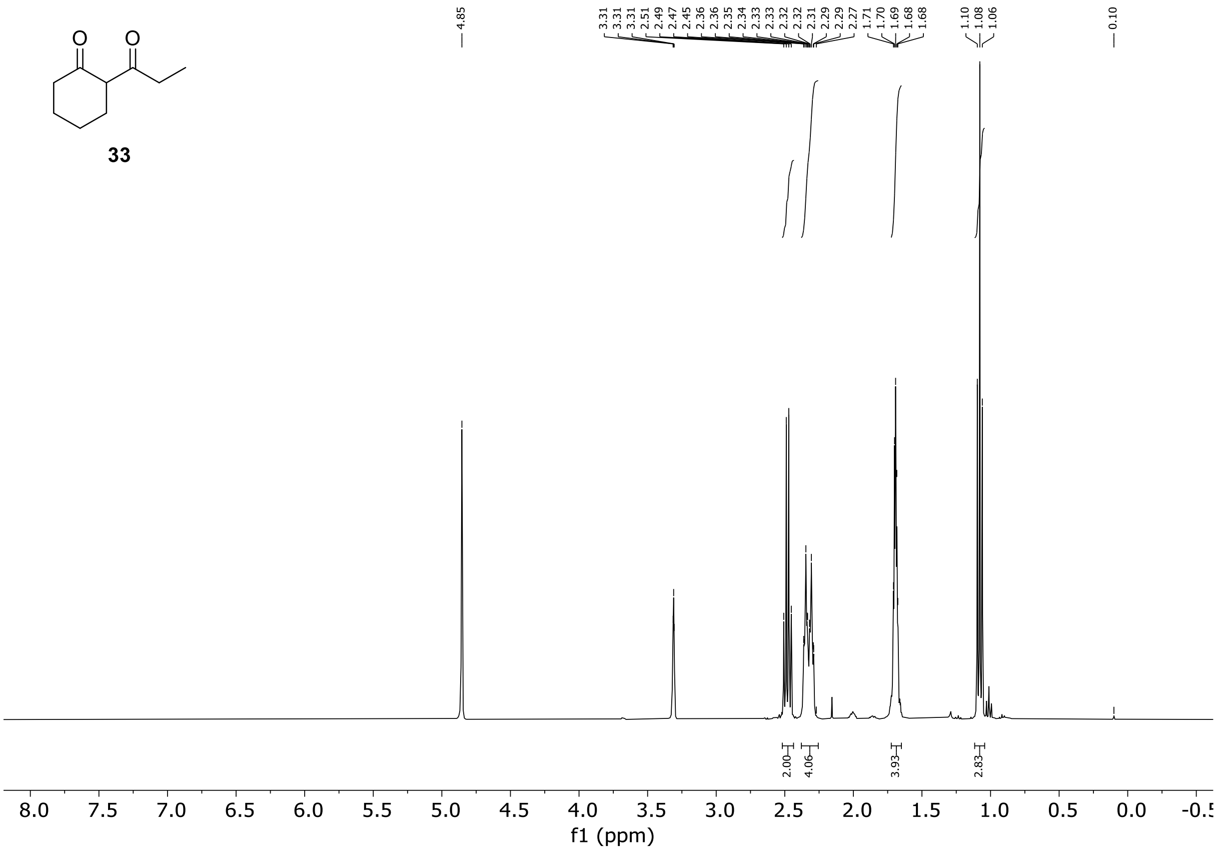
O
O
33
4.85
3.31
3.31
3.31
2.51
2.49
2.47
2.45
2.36
2.36
2.35
2.34
2.33
2.33
2.32
2.32
2.31
2.29
2.29
2.27
1.71
1.70
1.69
1.68
1.68
1.10
1.08
1.06
0.10
2.00
4.06
3.93
2.83
8.0 7.5 7.0 6.5 6.0 5.5 5.0 4.5 4.0 3.5 3.0 2.5 2.0 1.5 1.0 0.5 0.0
f1 (ppm)

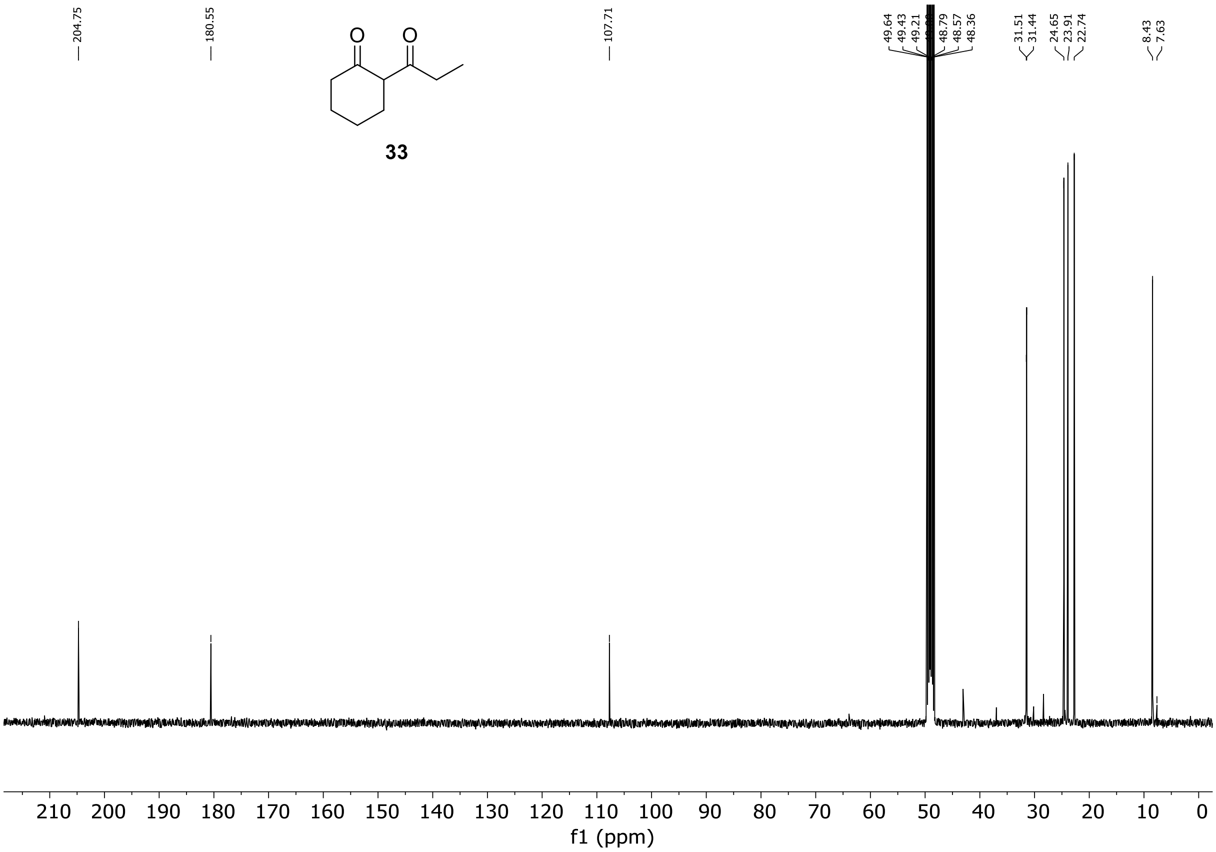
204.75
180.55
107.71
49.64
49.43
49.21
48.79
48.57
48.36
31.51
31.44
24.65
23.91
22.74
8.43
7.63
33
210 200 190 180 170 160 150 140 130 120 110 100 90 80 70 60 50 40 30 20 10 0
f1 (ppm)

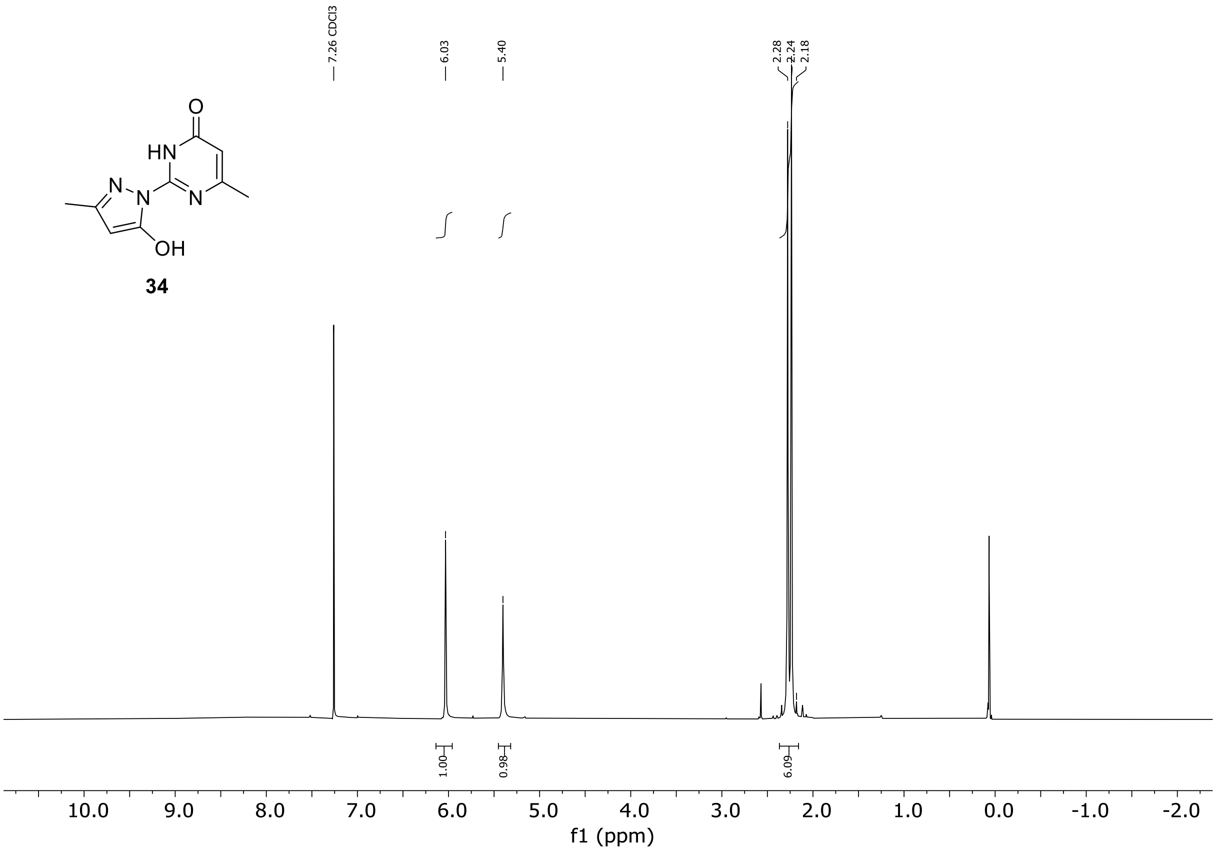

7.26 CDCl3
6.03
5.40
2.28
2.24
2.18
HN
N
O
OH
34
1.00
0.98
6.09
10.0 9.0 8.0 7.0 6.0 5.0 4.0 3.0 2.0 1.0 0.0 -1.0 -2.0
f1 (ppm)

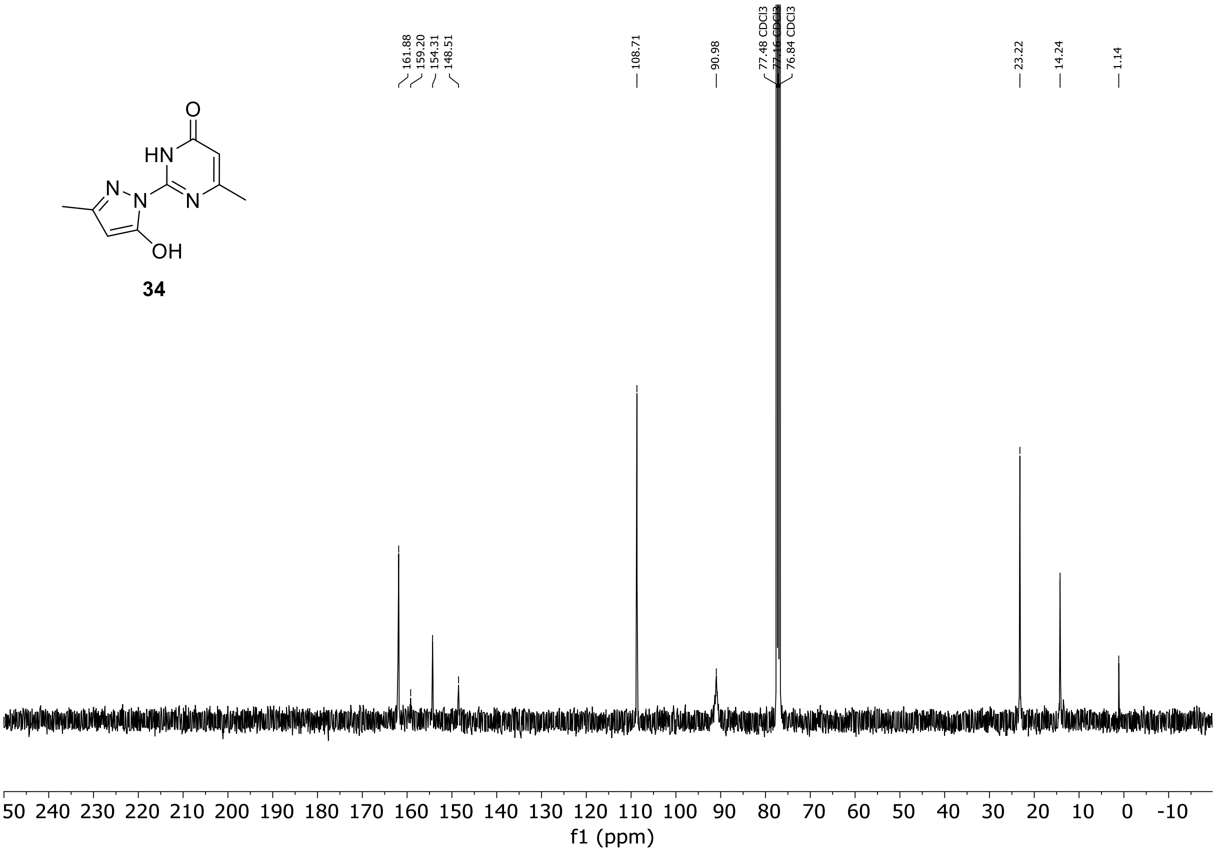

161.88
159.20
154.31
148.51
108.71
90.98
77.48 CDCl3
77.16 CDCl3
76.84 CDCl3
23.22
14.24
1.14
HN
N
O
OH
34
50 240 230 220 210 200 190 180 170 160 150 140 130 120 110 100 90 80 70 60 50 40 30 20 10 0 -10
f1 (ppm)

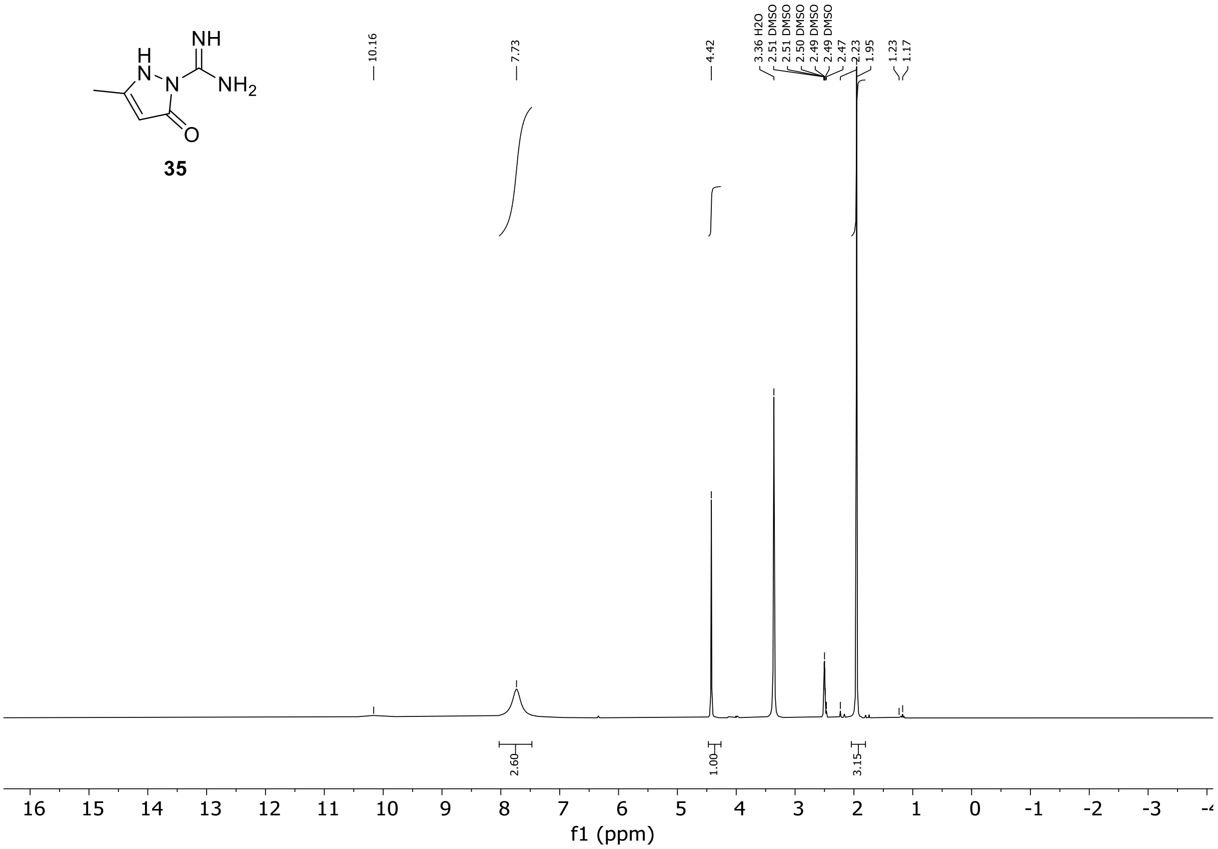

NH
H
N–N
NH2
O
35
10.16
7.73
4.42
3.36 H2O
2.51 DMSO
2.51 DMSO
2.50 DMSO
2.49 DMSO
2.49 DMSO
2.47
2.23
1.95
1.23
1.17
2.60
1.00
3.15
16 15 14 13 12 11 10 9 8 7 6 5 4 3 2 1 0 -1 -2 -3
f1 (ppm)

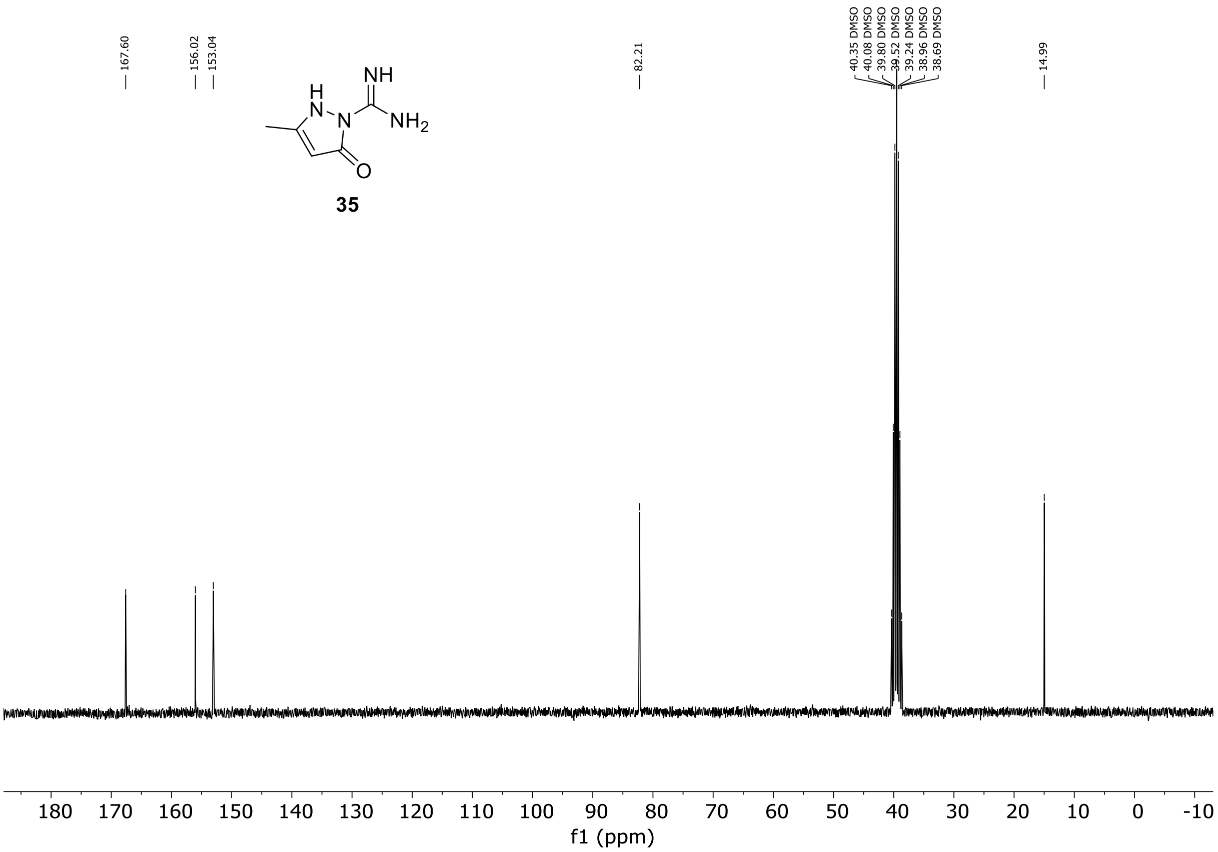

167.60
156.02
153.04
NH
H
N–N
NH2
O
35
82.21
40.35 DMSO
40.08 DMSO
39.80 DMSO
39.52 DMSO
39.24 DMSO
38.96 DMSO
38.69 DMSO
14.99
180 170 160 150 140 130 120 110 100 90 80 70 60 50 40 30 20 10 0 -10
f1 (ppm)
